# The Antioxidant and Antiproliferative Activities of 1,2,3-Triazolyl-L-Ascorbic Acid Derivatives

**DOI:** 10.3390/ijms20194735

**Published:** 2019-09-24

**Authors:** Anja Harej, Andrijana Meščić Macan, Višnja Stepanić, Marko Klobučar, Krešimir Pavelić, Sandra Kraljević Pavelić, Silvana Raić-Malić

**Affiliations:** 1 Centre for High-throughput Technologies, Department of Biotechnology, University of Rijeka, Radmile Matejčić 2, 51000 Rijeka, Croatia; aharej@biotech.uniri.hr (A.H.); mklobucar@biotech.uniri.hr (M.K.); 2 Department of Organic Chemistry, Faculty of Chemical Engineering and Technology, University of Zagreb, Marulićev trg 20, 10000 Zagreb, Croatia; sraic@fkit.hr; 3 Division of Electronics, Ruđer Bošković Institute, Bijenička cesta 54, 10000 Zagreb, Croatia; Visnja.Stepanic@irb.hr; 4 Faculty of medicine, Juraj Dobrila University of Pula, Zagrebačka 30, 52100 Pula, Croatia; kresimir.pavelic@unipu.hr

**Keywords:** vitamin C, 1,2,3-triazole, antioxidant, quantum-chemical modelling, antitumor, HIF-1

## Abstract

The novel 4-substituted 1,2,3-triazole L-ascorbic acid (L-ASA) conjugates with hydroxyethylene spacer as well as their conformationally restricted 4,5-unsaturated analogues were synthesized as potential antioxidant and antiproliferative agents. An evaluation of the antioxidant activity of novel compounds showed that the majority of the 4,5-unsaturated L-ASA derivatives showed a better antioxidant activity compared to their saturated counterparts. *m*-Hydroxyphenyl (**7j**), *p*-pentylphenyl (**7k**) and 2-hydroxyethyl (**7q**) substituted 4,5-unsaturated 1,2,3-triazole L-ASA derivatives exhibited very efficient and rapid (within 5 min) 2,2-diphenyl-1-picrylhydrazyl (DPPH^•^) radical scavenging activity (**7j**, **7k**: IC_50_ = 0.06 mM; **7q**: IC_50_ = 0.07 mM). In vitro scavenging activity data were supported by in silico quantum-chemical modelling. Thermodynamic parameters for hydrogen-atom transfer and electron-transfer radical scavenging pathways of anions deprotonated at C2-OH or C3-OH groups of L-ASA fragments were calculated. The structure activity analysis (SAR) through principal component analysis indicated radical scavenging activity by the participation of OH group with favorable reaction parameters: the C3-OH group of saturated C4-C5(OH) derivatives and the C2-OH group of their unsaturated C4=C5 analogues. The antiproliferative evaluation showed that *p-*bromophenyl (**4e**: IC_50_ = 6.72 μM) and *p-*pentylphenyl-substituted 1,2,3-triazole L-ASA conjugate (**4k**: IC_50_ = 26.91 μM) had a selective cytotoxic effect on breast adenocarcinoma MCF-7 cells. Moreover, compound **4e** did not inhibit the growth of foreskin fibroblasts (IC_50_ > 100 μM). In MCF-7 cells treated with **4e**, a significant increase of hydroxylated hypoxia-inducible transcription factor 1 alpha (HIF-1α) expression and decreased expression of nitric oxide synthase 2 (NOS2) were observed, suggesting the involvement of **4e** in the HIF-1α signaling pathway for its strong growth-inhibition effect on MCF-7 cells.

## 1. Introduction

Biochemical processes in our body, including aerobic metabolism and inflammatory responses, as well as exposure to the environment, result in the generation of unstable and highly reactive free radicals, such as reactive oxygen (ROS) and nitrogen (RNS) species [1,2,3,4]. While ROS have an important biological role in cell signaling, homeostasis and defense against micro-organisms, an imbalance between ROS production and ROS removal through the cell antioxidant protection system, comprised of endogenous antioxidants, enzymes, dietary antioxidants, and metal-binding proteins, can induce oxidative stress and various pathological conditions [2,5]. Free radicals may damage cellular components as they react with membrane lipids, nucleic acids, proteins, carbohydrates, and other small molecules [6,7]. Increasing evidence indeed, suggests that many chronic diseases, such as cancer, cardiovascular diseases, inflammation, neurodegenerative diseases, aging, diabetes, and atherosclerosis, are associated with high levels of ROS [8,9].

Vitamin C (L-ascorbic acid, L-ASA) or more accurately the ascorbate anion (A, Figure 2), is an excellent reducing agent and a powerful antioxidant co-factor required for key enzyme reactions [10,11,12,13,14]. Due to the low one electron reduction potential of the ascorbate radical (RA)/ascorbate (A) couple (Figure 2), almost every oxidizing radical formed in the biological system, including the hydroxyl (HO^•^), alkoxyl, peroxyl, thiol, and tocopheroxyl radicals cause one-electron oxidation of the ascorbate anion, resulting in the formation of the resonance stabilized ascorbate radical which can be recycled back to ascorbate [15,16]. Furthermore, structural modifications of hydroxyl groups of L-ASA by the introduction of lipophilic moieties have led to L-ASA derivatives with improved radical scavenging ability compared to L-ASA, especially in lipophilic parts of biological system [17,18,19,20,21,22]. While L-ASA has an antioxidant property and protects the cell from damage caused by free radicals, when used in pharmacological (millimolar) concentrations achieved through intravenous administration L-ASA shows pro-oxidant activity [13,23,24,25]. The pro-oxidant activity can be explained by the ability of ascorbate to reduce catalytic metals, for instance, Fe^3+^ to Fe^2+^, which than leads to the generation of the superoxide anion, hydrogen peroxide and HO^•^ by Fenton type reactions [26]. The higher concentration of ROS in this case, may lead to a cancer cell’s death.

Recent findings showed that high levels of vitamin C selectively kill colorectal cancer cells with *KRAS* and *BRAF* mutations by increasing ROS production, which blocks glucose metabolism and subsequently causes the energy crisis and cell death [27,28]. Additionally, high-doses of vitamin C showed a cytotoxic effect on breast adenocarcinoma (MCF7) and colon cancer (HT29) cells by inducing metabolic changes, which caused insufficient ATP formation and cell death [29]. Additionally, high concentrations of vitamin C showed cellular toxicity on proliferating neural stem/progenitor cells [30]. Furthermore, some lipophilic ASA derivatives with modified hydroxyl groups, as well as conjugates of ASA with pyrimidine, purine, triazole and imidazole bases, exhibited antitumor and antiviral activity [14].

Additionally, it was found that ascorbate decreases the activity of the hypoxia-inducible transcription factor 1 (HIF-1) [31,32,33,34]. As a response to hypoxic stress, HIF-1 and HIF-2 are overexpressed in human cancers, and upregulate the expression of genes involved in angiogenesis, glucose uptake, anaerobic metabolism, and cell proliferation, making HIF an important target for cancer therapy [13,35,36,37]. Under normoxic conditions, the HIF-1α subunit is continually synthesized and then degraded in the proteasome which requires HIF-hydroxylase enzymes to hydroxylate its proline and asparagine residues. It is presumed that ascorbate decreases the HIF-1α protein levels by acting as a co-factor for iron-dependent HIF-hydroxylase enzymes [38,39]. 

**Scheme 1:**
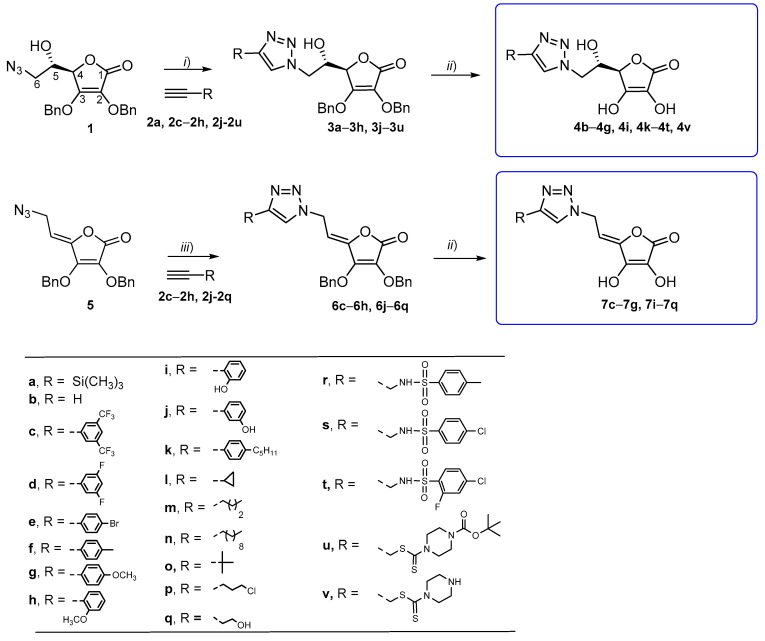
Reagents and conditions: (i) Cu, 1 M copper(II) sulphate (CuSO_4_), tert-butanol, DMF, water, and a microwave reactor; (ii) 1 M boron trichloride (BCl_3_) and dry dichloromethane; (iii) copper(II) acetate (Cu(OAc)_2_) and methanol.

Encouraged by the observed antitumor effect of vitamin C and its derivatives, in our previous work, we designed and synthesized 2,3-dibenzylated L-ASA derivatives and their C4=C5 unsaturated analogues with a 1,4-disubstituted 1,2,3-triazole core on the C-6 atom of the lactone ring that exhibited a pronounced and selective antitumoral and antiviral activity [40]. In continuation of our work and with the aim to enhance the antioxidant activity of L-ASA, we have prepared a library of 1,2,3-triazole L-ASA conjugates (Scheme 1) with free C-2 and C-3 hydroxyl groups, hypothesizing that diverse substituted triazole moiety and hydroxyethylene and ethylidene linkers will have an impact on free-radical-scavenging and antitumor properties. 

## 2. Results and Discussion

### 2.1. Synthesis

The target 1,2,3-triazole appended L-ASA derivatives were synthesized following Scheme 1. The 1,2,3-triazole ring was introduced at the C-6 position of L-ascorbic acid via copper(I)-catalyzed azide-alkyne (3 + 2) dipolar cycloaddition (CuAAC) [41] of the corresponding C-6 azido L-ascorbic acid derivatives **1** and **5** with versatile terminal alkynes (**2a**, **2c–2h**, **2j–2u**) and 6-(1,2,3-triazolyl)-6-deoxy (**3a–3h**, **3j–3u**). Then, 6-(1,2,3-triazolyl)-4,5-didehydro-5,6-dideoxy-L-ASA (**6c–6h**, **6j–6g**) derivatives were obtained [40]. 

The removal of the benzyl group was achieved via reaction of the 1,2,3-triazole L-ascorbic acid derivatives **3a–3h**, **3j–3u**, **6c–6h**, and **6j–6q** with boron trichloride (BCl_3_) in dry dichloromethane, affording compounds **4b–4q**, **4i**, **4k–4t**, **4v**, **7c–7g**, and **7i–7q** with yields from 42% to 86%. 

### 2.2. DPPH^•^ Radical Scavenging Activity in Vitro

Likewise, L-ASA, the 1,2,3-triazole L-ASA derivatives with free C2-OH and C3-OH groups were expected to be potent antioxidants. Therefore, they were tested as radical scavengers by reaction with the stable free radical 2,2-diphenyl-1-picrylhydrazyl (DPPH^•^). The scavenging activity data are expressed as IC_50_ values and are summarized in Table 1.

L-ASA was used as control and its experimentally assessed IC_50_ was 0.18 mM for the 5 min treatment. The scavenging activity data showed that conjugates having a hydroxyethylene spacer with a 4-substituted 1,2,3-triazole moiety at the C-6 position of L-ASA (**4b**−**4v**), in general, exhibited activity similar to that of L-ASA. Compounds **4b**, **4f**, **4k**, **4l**, **4n**, **4p**, and **4t** from the 6-(1,2,3-triazolyl)-6-deoxy-L-ASA and **7f**, **7i**−**7k**, **7n**, and **7q** from the 6-(1,2,3-triazolyl)-4,5-didehydro-5,6-dideoxy-L-ASA series had higher antioxidant potencies in comparison with L-ASA. When a halogen (**4c**, **4d**, or **4e**) and methoxy-substituted (**4g**) aryl moiety was introduced in the C-4 of 1,2,3-triazole, the antioxidant activity was slightly lower (IC_50_ = 0.19–0.27 mM) in comparison with L-ASA and the unsubstituted 1,2,3-triazole L-ASA derivative **4b** (IC_50_ = 0.13 mM at 5 and 10 min treatments). The introduction of an alkyl in the para position of phenyl in compounds **4f** and **4k**, improved the antioxidant activity after the 10 min treatment. Among 4-alkyl-substituted triazoles, cyclopropyl in **4l**, decyl in **4n**, and 3-chloropropyl substituents in **4p** also improved the antioxidant activity compared to L-ASA. L-ASA derivatives containing the benzenesulphonamide and dithiocarbamate moiety showed lower radical scavenging activity after the 5 min treatment then L-ASA and the unsubstituted triazole **4b**, but benzenesulphonamide-substituted 1,2,3-triazole L-ASA derivative **4t** had a better antioxidant activity after the 10 min treatment then L-ASA and **4b**, with an IC_50_ value of 0.12 mM. 

**Figure 1:**
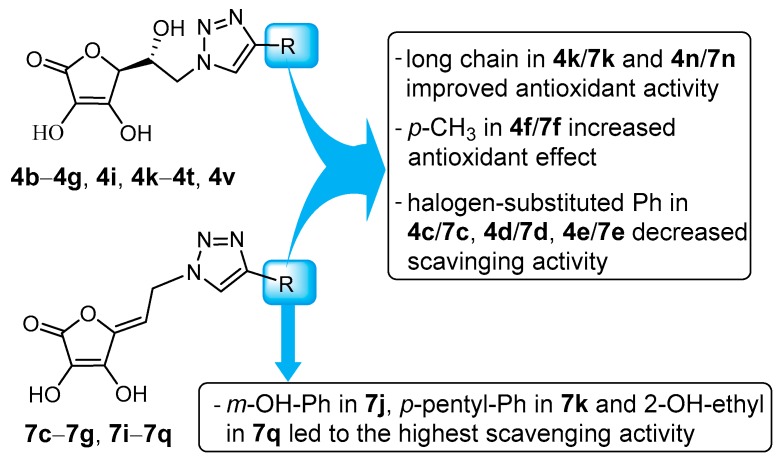
Summarized representation of the importance of some structural features in antioxidant/radical-scavenging activity. The arrows point to structure-activity observations.

It was observed that the majority of the 4,5-unsaturated L-ASA derivatives showed a better antioxidant activity compared to their saturated counterparts (Figure 1). Interestingly, the introduction of hydroxyl groups in 4,5-unsaturated L-ASA derivatives **7i**, **7j**, and **7q** improved the radical scavenging activities. Besides, the electron-donating *p*-methyl groups in **4f**/**7f**, the *p*-pentyl-substituted aryl groups in **4k**/**7k**, and the long aliphatic chains in **4n**/**7n** improved the antioxidant activity compared to L-ASA as well.

### 2.3. In Silico Analysis of Radical Scavenging Capacities

According to the pIC_50_ values, calculated as the negative logarithm of IC_50_ values (Table 1)—which are within the range of 3.55−4.22 and have the pIC_50_ medians of 3.7, regardless the incubation time of 5 min or 10 min—the majority of L-ASA derivatives have similar DPPH^•^ radical scavenging capacities. The pIC_50_ value for the well-known antioxidant L-ASA was 3.75, demonstrating that most of compounds have a similar or somewhat higher radical scavenging capacity compared to L-ASA.

For some pairs of L-ASA derivatives differing in the presence of C4-C5(OH) or C4=C5 fragments, somewhat greater difference in their IC_50_ values has been observed (Table 1). For these pairs of compounds—**4b**/**7b**, **4e**/**7e**, **4g**/**7g**, **4i**/**7i** and **4q**/**7q**—the extensive quantum-chemical calculations of radical scavenging capacities were performed by using an approach widely exploited for the estimation of the radical scavenging potential of polyphenols [42]. L-ASA is highly dissociated acidic molecule at physiological pH of 7.4 (the negative logarithms of experimental acid dissociation constants (pK_a_) are pK_a,1_ of 4.1 and pK_a,2_ of 11.4) [43]. 

**Figure 2:**
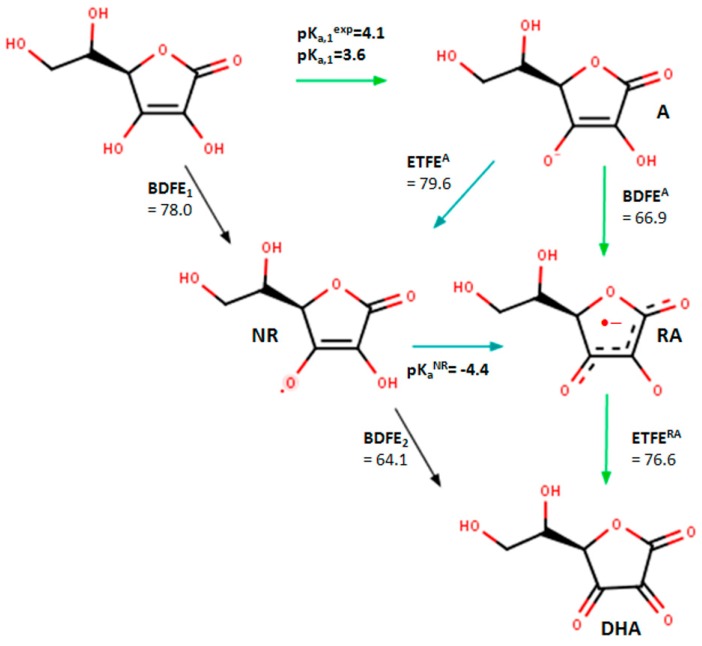
Radical scavenging mechanisms and associated calculated aqueous free energies (bond dissociation free energy (BDFE) and electron transfer free energy (ETFE) in kcal/mol) and negative logarithm of acid dissociation constant for the most acidic OH group, pKa,1, Table 2), of L-ascorbic acid (L-ASA) at physiological pH. The ascorbate anion deprotonated at the *O*-3 position may neutralize a free radical by the donation of an H-atom (green pathway) or an electron (cyan pathway). The intermediate radical anion transforms to the final product, dehydroascorbic acid (DHA), by further giving an electron. The synthesized L-ASA derivatives may follow the same radical scavenging mechanisms.

**Figure 3:**
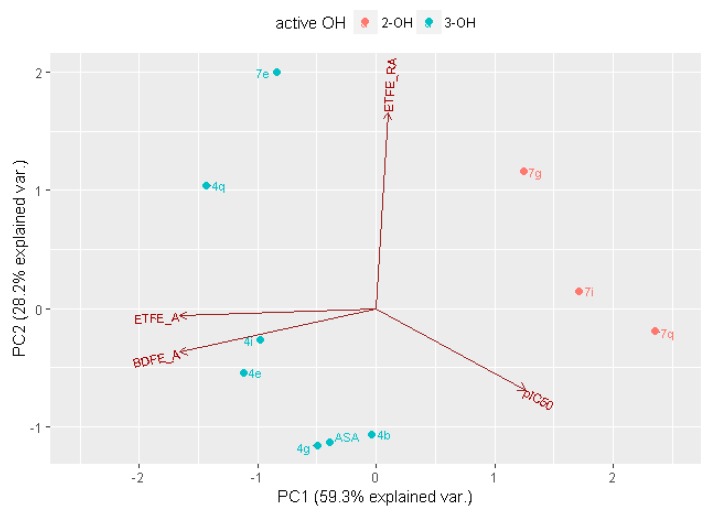
Principal component analysis (PCA) biplot for derivatives containing C4C5(OH) (series **4**) or C4=C5 (series **7**) fragments obtained by the PCA analysis performed in terms of the in vitro pIC_50_ values and three significant in silico radical scavenging parameters (Table 2). For saturated/unsaturated derivatives, the more favorable parameter values for C3-O^−^/C2-O^−^ anions have been used. The first two principal components explain 87.5% variance in the data set. Coloring is according to the active OH group in the 1st radical scavenging step. More active compounds have the higher pIC_50_ values and are more on the right side of the plot.

The novel 1,2,3-triazole L-ASA conjugates were shown to be acidic molecules with similar pK_a_ values (Table 2). Accordingly, L-ASA reacts as a radical scavenger by donating an electron or a hydrogen H-atom from its anion to a free radical (Figure 2). The first radical scavenging mechanism of L-ASA is described as a two-step sequential proton loss electron transfer (SPLET) process: after the first step of deprotonation of the most acidic OH group (described by its acidity constant), an anion (A) formed donates an electron with a capacity described by the electron transfer (free) energy (ETFE) and changes into neutral radical (NR) (cyan path in Figure 2). However, an anion may also donate a H-atom from the adjacent OH group through the hydrogen-atom transfer (HAT) mechanism which is described by the O-H bond dissociation free energy (BDFE) parameter. The product of this radical scavenging pathway is a radical anion (RA) which may release an additional electron and oxidize to a diketo form of L-ASA known as dehydroascorbic acid (DHA, green path in Figure 2). However, the neutral radical formed by SPLET, immediately releases a proton (pK_a_^NR^ in Table 2) and converts to a RA. Thus, both pathways have the same final dehydroascorbic acid product. The thermodynamic preference of one of these two pathways has been estimated by comparison of reaction free energies of underlying step processes (Table 2). The multivariate principal component analysis (PCA) analysis in terms of the experimental pIC_50_ values and the calculated aqueous parameters ETFE^A^, BDFE^A^, and ETFE^RA^ describing radical scavenging capacities of reducing anions and the intermediate radical anion, explain 87.5% variance in the DPPH^•^ activity of modelled compounds (Figure 3). 

The most significant difference between saturated C4-C5(OH) and unsaturated C4=C5 derivatives is in acidity of the C2-OH group. While in saturated C4-C5(OH) L-ASA derivatives, the C3-OH group is the only one deprotonated at pH 7.4, in derivatives with the C4=C5 bond, both C2-OH group and C3-OH groups are acidic and may participate in the described DPPH^•^ radical scavenging mechanisms (Table 2). However, in the C4=C5 derivatives, intermediate C2-O^–^ anion is generally characterized by more favorable, lower ETFE^A^ and BDFE^A^ values than the C3-O^–^ anion and it may be expected that it is a primary reactive locus for scavenging of the DPPH^•^ radical (Table 2). Thus, the best variance explanation in the PCA analysis of DPPH^•^ radical scavenging activity has been obtained by using the more favorable parameter values for C3-OH groups of saturated C4-C5(OH) derivatives and for the C2-OH group of the C4=C5 unsaturated derivatives, except for the compound **7e**, for which C3-OH parameters were used (Figure 3). 

By comparing ETFE^A^, BDFE^A^, and ETFE^RA^ values for saturated and unsaturated analogues, all three parameters are clearly more favorable for both groups C2-OH and C3-OH of **7q** than for the C3-OH group of its saturated analogue **4q**, and it has been observed as significantly more active in DPPH^•^ radical scavenging, having a higher pIC_50_ value (Table 1 and Table 2). ETFE^A^ and BDFE^A^ are also more favorable for the stronger scavenger **7i** in the **4i**/**7i** pair. While BDFE^A^ values for the **4e**/**7e** pair are comparable, the radical scavenging capacity of intermediate radical anion is somewhat better for **4e** than **7e**, which may explain the somewhat higher pIC_50_ value of the saturated analogue **4e**. However, according to the calculation, unsaturated **7g** should be a stronger scavenger than the saturated **4g**, which was not in agreement with the measured pIC_50_ values during 5 min and 10 min time periods of incubation.

### 2.4. Biological Evaluations

#### 2.4.1. Antiproliferative Activities in Vitro

The C-6 substituted 1,2,3-triazole derivatives of L-ASA within the C5-OH group (**4b**−**4q**, **4i**, **4k**−**4t**, and **4v**) and with the C4=C5 double bond (**7c**−**7g** and **7i**−**7q**) were evaluated against seven malignant tumor cell lines: lung adenocarcinoma (A549), ductal pancreatic adenocarcinoma (CFPAC-1), colorectal carcinoma (HCT-116), cervical carcinoma (HeLa), hepatocellular carcinoma (HepG2), breast adenocarcinoma (MCF-7), colorectal adenocarcinoma, and metastatic (SW620); as well as normal cell lines: lung fibroblasts (WI-38) and foreskin fibroblasts (HFF-1) (Table 3, Table S1, Supplementary Information). L-ASA was used as a positive control, and carboxyamidotriazole, a known antiproliferative agent that inhibits tumor cell growth, invasion, and metastasis, and 5-fluorouracil (5-FU), were used as reference compounds.

It can be observed that the majority of C-4 substituted 1,2,3-triazolyl-6-deoxy-L-ASA derivatives were not cytotoxic. While compound **4e** showed a selective antiproliferative effect on MCF-7 and A549 cells and **4k** on MCF-7 cells, compounds **4d**, **4o**−**4q**, and **4v** showed an only minor antiproliferative effect at the highest tested concentrations (Table 1). However, except for **4e**, **4q** and **4v**, other compounds exhibited cytotoxicity on foreskin fibroblasts HFF-1 as well. The compounds showed themselves to be more active than L-ASA, which showed no effects on the cell growth of the cells tested. Carboxyamidotriazole, that contains a 1,2,3-triazole ring like the other tested compounds, showed a non-selective, moderate antiproliferative effect on all evaluated cell lines.

The unsubstituted 1,2,3-triazolyl derivative **4b** was not cytotoxic toward any of the tested cell lines, indicating that C-4 substitution at 1,2,3-triazole moiety has an impact on growth-inhibition. Among the aryl-substituted triazole derivatives, the fluoro and bromo-substituted aromatic moieties in **4d** and **4e**, respectively, had influences on the antiproliferative effect. While **4d** exhibited a nonspecific cytostatic effect, **4e** displayed a strong and selective antiproliferative effect on breast adenocarcinoma MCF-7 (IC_50_ = 6.72 µM) and moderate activity on lung adenocarcinoma A549 cells (IC_50_ = 25.44 µM). Additionally, compound **4e** did not exhibit cytotoxicity against foreskin fibroblasts HFF-1 (IC_50_ > 100 µM) and had some cytotoxicity on fibroblasts WI-38 (IC_50_ = 73.93 µM; selectivity index, SI = 11). When comparing the activities of compounds **4e**, **4f**, and **4g** it can be concluded that the nature of the substituent in the *p*-position of the phenyl has an influence on the antiproliferative effect. While electron-withdrawing bromine in the structure of **4e** increased the activity, compounds **4f** and **4g** with electron-donating groups on the 4-aryl-1,2,3-triazole units, did not show any cytotoxic effects. The only exception to the observed impact of electron-donating groups on the inhibition effect was *p*-pentylphenyl-1,2,3-triazolyl-6-deoxy-L-ASA derivative **4k** that exerted a selective cytotoxic effect on MCF-7 cells (IC_50_ = 26.91 µM), albeit, it showed cytotoxicity on foreskin fibroblasts (IC_50_ = 0.21 µM). From the 4-alkyl substituted triazole derivatives, 4-*tert*-butyl in **4o**, 3-chloropropyl in **4p**, and 2-hydroxyethyl chains in **4g** caused moderate to marginal cytostatic effects on cervical carcinoma HeLa, colorectal carcinoma HCT-116, and breast adenocarcinoma MCF-7 cells. Compounds **4r**−**4t** with benzenesulphonamide substituents, did not show any cytotoxicity toward evaluated tumor cell lines. The dithiocarbamate derivative **4v** showed a selective but moderate antiproliferative effect toward MCF-7 cells (IC_50_ = 37.33 µM). Referring to the lipophilicity of the newly synthesized compounds, their clogP values are within the range of −3.00–2.67 (Table 3 and Table S1, Supplementary Information). Compounds **4e** and **4k** with the best inhibitory activity and clogP values of −0.42 and 0.98, respectively, showed themselves to be more lipophilic than L-ASA (clogP = −2.46).

The C-6 substituted 1,2,3-triazole 4,5-unsaturated L-ASA derivatives (**7c**−**7g** and **7i**−**7q**) showed no antiproliferative effect on the tested cell panel at concentrations lower than 100 µM (Table S1, Supplementary Information), which implies that the linker connecting the lactone ring and the 1,2,3-triazole moiety has an influence on the antitumor activity of tested compounds, which is in accordance with the previously shown antitumor effects of 2,3-*O,O*-dibenzylated L-ASA derivatives [40].

#### 2.4.2. The Detection of Apoptosis and the Validation of HIF-1α and NOS2 Protein Targets for **4e**

In order to examine whether the antitumor effect of *p*-bromophenyl-substituted L-ASA derivative **4e**, which showed a selective antiproliferative effect on MCF-7 tumor cell line, can be connected to the induction of apoptosis or any other kind of cell death, an Annexin V assay was performed following a procedure described previously [45].

The results obtained indicate that compound **4e** induced apoptosis in MCF-7, and showed a moderate increase in early apoptotic cell populations of 12.4% and late apoptotic cell populations, 2.0%, after 24 h-treatment at concentration 1 × IC_50_ (Table 4). After 48 h the cell death rate in the treated cells was similar to the untreated MCF-7 control cells for the higher tested concentration, while the percentage of cells in early apoptosis was higher by 35.2%, in treated cells at concentration 1 × IC_50_, compared with untreated cells.

**Figure 4:**
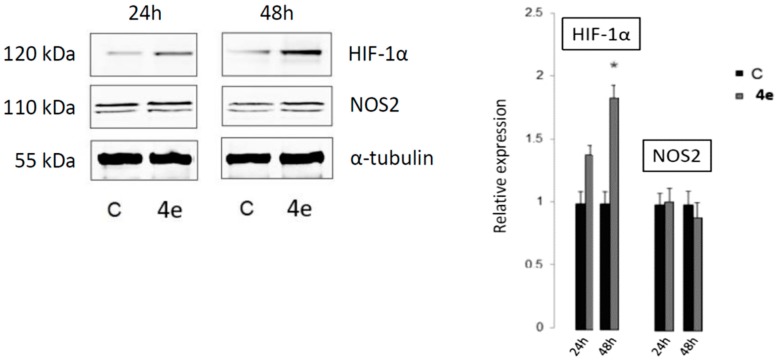
Western blot analysis of predicted protein targets of compounds **4e**. Representative western blots are shown on the left panel, where cellular levels of selected proteins before and after treatment of MCF-7 cells with compound **4e**, at IC50 = 6.72 µM for 24 and 48 h, can be observed. C denotes control (untreated) cells. Approximate molecular weights (kDa) of target proteins are presented. Relative signal intensities of target proteins were normalized to the alpha-tubulin loading control and are shown in the right panel. Data are presented as mean values ± SDs. Statistically significant (*p* < 0.5) differences in the expression levels are marked by an asterisk (*).

To further understand the observed antiproliferative and cell death effects on MCF-7 cells by *p*-bromophenyl 1,2,3-triazole L-ASA conjugate **4e**, the analysis of the relative expressions of Hypoxia-inducible factor I alpha (HIF-1α) and Nitric oxide synthase 2 (NOS2, iNOS) protein, as potential targets of **4e**, was performed by western blot (Figure 4). The results showed that in MCF-7 cells treated with **4e** for 48 h, a statistically significant increase of hydroxylated HIF-1α (Pro564) expression was observed. Increased levels of hydroxylated HIF-1α (Pro564) would imply an increased form of HIF-1α for proteasomal degradation. Furthermore, 48 h after incubation of MCF-7 cells with **4e**, a decreased expression of NOS2 was observed.

NOS2s are homodimer enzymes that catalyze the formation of nitric oxide radicals (NO) from L-arginine and oxygen. NO is an important signaling molecule and mediates various physiological and pathological processes, including systemic blood pressure, the upregulation of hypoxic genes, the regulation of stress response pathways, host–microbe interactions, immune signaling, and apoptosis [46,47,48]. The majority of reports indicate that NO induces HIF-1α accumulation but the molecular mechanisms of the regulation of HIF-1α activity by NO are not yet defined and are dependent on conditions being hypoxic or normoxic, and on the concentration of NO [47,48,49,50,51,52,53]. Additionally, it was reported that vitamin C reduces the NO-induced stabilization of HIF-1α in endothelial cells from umbilical cords (HUVECs) under normoxic conditions [54].

Based on the literature overview, we can conclude that the decreased expression of NOS2 is consistent with the enhanced expression of hydroxylated HIF-1α and the antiproliferative effects of **4e** on MCF-7 observed.

#### 2.4.3. Cell Metabolism Analysis—Mitochondrial Toxicity

**Figure 5:**
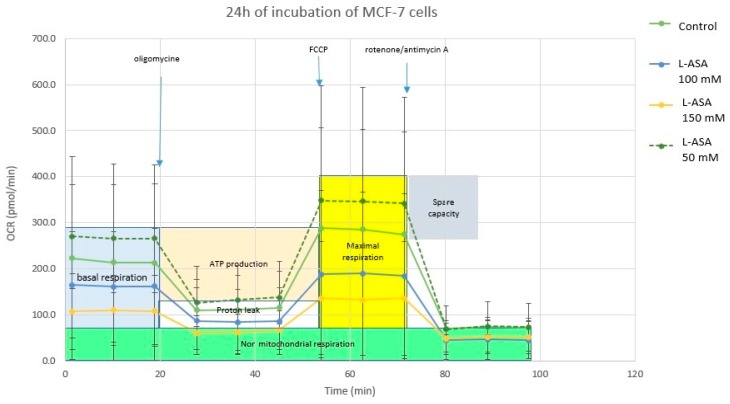

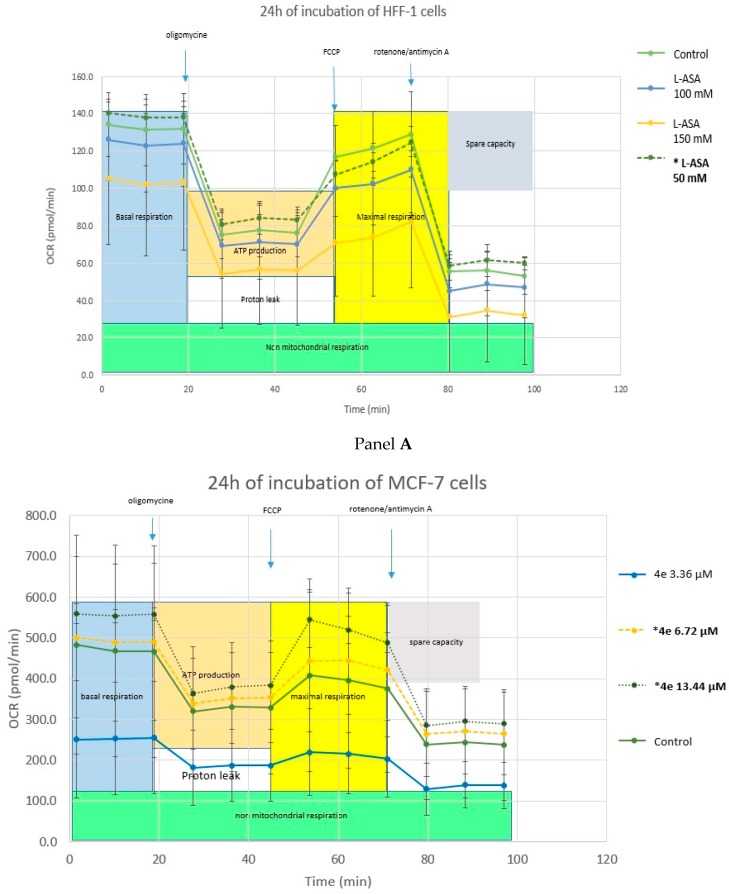

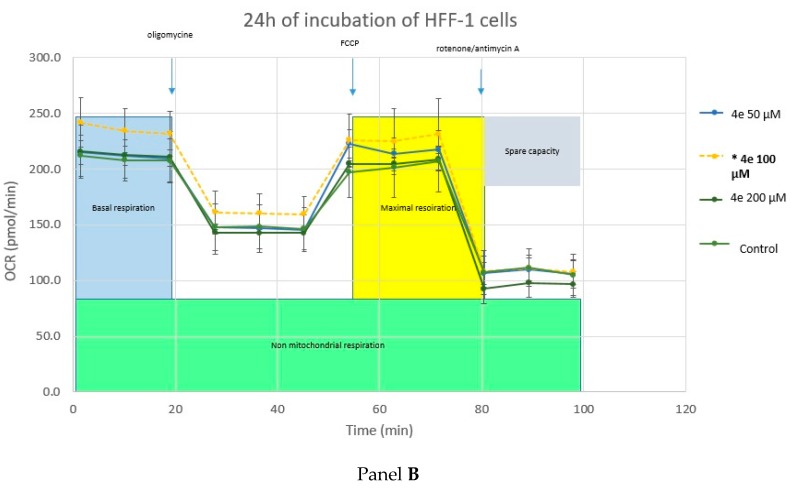
Results of the cell metabolism analysis for cells MCF-7 and HFF1 treated for 6 and 24 h with compound **4e** having a *p*-bromophenyl substituent at C-4 of triazole. *The zero (0) on the X axis denotes the starting point of measurment upon addition of oligomycine, mitochondrial oxidative phosphorylation uncoupler carbonyl cyanide-4-(trifluoromethoxy)phenylhydrazone (FCCP), and rotenone/antimycin A, respectively (marked with arrows), on cells incubated with L-ASA or **4e** for 24 h. Panel **A**—cells treated with L-ASA; Panel **B**—cells treated with compound **4e.**

The effects on the mitochondrial respiration after 24 h exposure of MCF-7 and HFF-1 cells to L-ASA and **4e** tested at concentrations comparable to the DPPH test (IC_50_ values and correspondingly higher or lower concentrations) were assessed by use of the Seahorse XF Cell Mito Stress Test kit (Figure 5). Even though no statistically relevant changes were observed, interesting trends were consistent. The basal respiration and ATP production of MCF-7 and HFF-1 cells after sequential injection of the standard assay compounds, as described in the Material and methods section, was affected by higher concentrations of L-ASA and compound **4e** that both induced a decrease in the cellular-metabolism rate. A lower L-ASA concentration (50 mM) did not act on the mitochondrial activity (dashed lines in Figure 5, panel A). In MCF-7 tumor cells, it was clearly visible that higher L-ASA concentrations decreased the basal cell respiration and ATP production in the way that the whole cell metabolism switched towards non-mitochondrial respiration while this effect was not observed in the normal HFF-1 fibroblasts. Compound **4e** increased the basal respiration and ATP production of MCF-7 cells at its IC_50_ concentration (6.72 µM) and 2 × IC_50_ concentration (13.44 µM), while it lowered cellular mitochondrial metabolism, switching it towards protein leaking at the lower tested concentration (3.36 µM). Compound **4e** did not affect the basal cell respiration or ATP production of HFF-1 cells which correlates with MTT-results where **4e** showed no cytotoxicity on this cell line at micromolar concentrations (Table 3; IC_50_ > 100 µM). Interestingly, **4e** showed a slightly positive effect on the cellular metabolism and mitochondrial activity of HFF-1 at a concentration of 100 µM (Figure 5, panel B). The observed differences in compound **4e** activity on MCF-7 tumor cells in comparison with normal fibroblasts HFF-1 may be due to the specific metabolic status of tumor cells, in which case an antioxidant compound with ROS scavenging properties, such as L-ASA, may have a cytotoxic effect; i.e., through a pro-oxidant effect on cancer cells associated with increase of ROS in tumor cells [55]. Particularly in MCF-7 cells, a small amount of an important cellular antioxidant enzyme manganese-dependent superoxide dismutase (MnSOD) was found in comparison with other cell lines [56]. It is therefore, possible that the compound **4e** exerts a pro-oxidant activity in MCF-7 cells, which elevates the levels of ROS and induces cell death. This hypothesis should be further studied, however, in more experimental detail.

## 3. Materials and Methods

### 3.1. Chemistry

#### 3.1.1. General

The melting points of novel compounds were determined using a Kofler micro hot-stage (Reichert, Wien, Austria) apparatus. The progress of all reactions was monitored by thin-layer chromatography (TLC) on silica gel 60F-254 plates (Merck, Darmstadt, Germany) and the spots were observed under UV light (254 nm). Purification of compounds using column chromatography was carried out with silica gel (0.063–0.2 mm) (Fluka, Buchs, Switzerland). All ^1^H and ^13^C NMR spectra were recorded in DMSO-d_6_ at 298 K on a Bruker 300 or 600 MHz NMR spectrometer (Bruker Biospin, Rheinstetten, Germany). Chemical shifts were referenced to the signal of DMSO at δ: 2.50 ppm (^1^H NMR) and δ: 39.50 ppm (^13^C NMR). High-resolution mass spectra (HRMS) of the final compounds were recorded on a 4800 Plus MALDI TOF/TOF mass spectrometar (Applied Biosystems, Foster City, CA, USA). Acquisitions were performed in positive ion reflectron mode. 

#### 3.1.2. Preparation of Compounds

General procedure for the removal of the benzyl groups: 1,2,3-triazole-L-ascorbic acid derivatives **3a**−**3h**, **3j**−**3u**, **6c**−**6h**, and **6j**−**6q** were dried using a vacuum pump. Dry dichloromethane (around 20 mL) was added to the compound under an argon atmosphere. The reaction mixture was cooled to −65 °C and 1 M boron trichloride solution (BCl_3_, 6 equivalents) was added dropwise to the reaction mixture. The reaction mixture was stirred for 3 h at −65 °C and was left in the freezer overnight. A mixture of dichloromethane and methanol (10 mL) was then added and the reaction mixture was stirred for 30 min at room temperature. The solvent was evaporated and Amberlite IRA 743 was added to neutralize the reaction mixture. The reaction mixture was filtered and the solvent was evaporated. The compounds were purified using a very short column filled with silica (CH_2_CH_2_:MeOH = 100:1; 50:1; 10:1). After trituration of the obtained product with n-hexane, diethyl ether, dichloromethane, acetone, and methanol, compounds **4b–4q**, **4i**, **4k–4t**, **4v**, **7c−7g**, and **7i−7q** were isolated as white powders.

*The preparation of 6-deoxy-6-(1,2,3-triazol-1-yl)-L-ascorbic acid* (**4b**): Compound **4b** (61.8 mg; 57%; m.p. = 219–221 °C) was synthesized according to the general procedure using compound **3a** (194.9 mg; 0.478 mmol); 1 M BCl_3_ (2.9 mL) and CH_2_Cl_2_ (20 mL). ^1^H NMR (600 MHz, DMSO) δ: 11.12 (bs, 1H, OH), 8.43 (bs, 1H, OH), 8.10 (s, 1H, H-8), 7.72 (s, 1H, H-7), 5.45 (bs, 1H, OH), 4.71 (d, *J* = 1.4 Hz, 1H, H-4), 4.65 (dd, *J* = 13.8 Hz, 3.9 Hz, 1H, H-6), 4.41 (dd, *J* = 13.8 Hz, 9.3 Hz, 1H, H-6), 4.19 (d, *J* = 6.4 Hz, 1H, H-5) ppm. ^13^C NMR (151 MHz, DMSO) δ: 170.3 (C-1), 152.0 (C-3), 133.0 (C-8), 125.8 (C-7), 118.3 (C-2), 75.6 (C-4), 67.2 (C-5), 52.4 (C-6) ppm. HRMS (ESI Q-TOF): calculated for C_8_H_9_N_3_O_5_ [M + H]^+^ = 228.0620; found = 228.0626.

*Preparation of 6-deoxy-6-[4-(3,5-bis(trifluoromethyl)phenyl)-1,2,3-triazol-1-yl]-L-ascorbic acid* (**4c**): Compound **4c** (119.0 mg; 86%; m.p. ≥ 250 °C) was synthesized according to the general procedure using compound **3c** (194.5 mg; 0.314 mmol); 1 M BCl_3_ (1.9 mL) and CH_2_Cl_2_ (20 mL). ^1^H NMR (600 MHz, DMSO) δ: 11.18 (s, 1H, OH), 8.99 (s, 1H, H-7), 8.53 (s, 2H, Ph-2, Ph-6), 8.46 (s, 1H, OH), 8.07 (s, 1H, Ph-4), 5.60 (d, *J* = 6.9 Hz, 1H, OH), 4.79 (d, *J* = 1.6 Hz, 1 H, H-4), 4.74 (dd, *J* = 13.8 Hz, 4.0 Hz, 1H, H-6), 4.46 (dd, *J* = 13.9 Hz, 9.3 Hz, 1H, H-6), 4.29–4.23 (m, 1H, H-5) ppm. ^13^C NMR (151 MHz, DMSO) δ: 170.2 (C-1), 151.9 (C-3), 143.4 (C-8), 133.4 (Ph-1), 131.4; 131.1; 130.9; 130.7 (q, ^2^*J_CF_* = 32.9 Hz, Ph-3, Ph-5), 126.0; 124.1; 122.3; 120.5 (q, ^1^*J*_CF_ = 272.8 Hz), 125.3 (Ph), 124.5 (Ph), 121.0 (C-7), 118.3 (C-2), 75.4 (C-4), 67.0 (C-5), 53.1 (C-6) ppm. HRMS (ESI Q-TOF): calculated for C_16_H_11_F_6_N_3_O_5_ [M + H]^+^ = 440.0681; found = 440.0671.

*Preparation of 6-deoxy-6-[4-(3,5-difluorophenyl)-1,2,3-triazol-1-yl]-L-ascorbic acid* (**4d**): Compound **4d** (44.4 mg; 46%; m.p. = 189–191 °C) was synthesized according to the general procedure using compound **3d** (148.6 mg; 0.286 mmol); 1 M BCl_3_ (1.7 mL) and CH_2_Cl_2_ (20 mL). ^1^H NMR (600 MHz, DMSO) δ: 11.16 (s, 1H, OH), 8.72 (s, 1H, H-7), 8.45 (s, 1H, OH), 7.59–7.56 (m, 2H, Ph-2, Ph-6), 7.35–7.10 (m, 1H, Ph-4), 5.57 (s, 1H, OH), 4.78 (d, *J* = 1.5 Hz, 1H, H-4), 4.71 (dd, *J* = 13.8 Hz, 3.8 Hz, 1H, H-6), 4.42 (dd, *J* = 13.8 Hz, 9.4 Hz, 1H, H-6), 4.26–4.22 (m, 1H, H-5) ppm. ^13^C NMR (151 MHz, DMSO) δ: 170.2 (C-1), 163.7; 163.6; 162.1; 162.0 (dd, ^1^*J*_CF_ = 245.6 Hz, ^3^*J*_CF_ = 13.7 Hz, Ph-3, Ph-5), 151.9 (C-3), 144.1 (C-8), 134.4; 134.3; 134.3 (t, ^3^*J*_CF_ = 10.7 Hz, Ph-1), 123.9 (C-7), 118.3 (C-2), 108.3–107.7 (m, Ph-2, Ph-6), 103.1; 103.0; 102.8 (t, ^2^*J*_CF_ = 25.9 Hz, Ph-4), 75.5 (C-4), 67.0 (C-5), 53.0 (C-6) ppm. HRMS (ESI Q-TOF): calculated for C_14_H_11_F_2_N_3_O_5_ [M + H]^+^ = 340.0745; found = 340.0750.

*Preparation of 6-[4-(4-bromophenyl)-1,2,3-triazol-1-yl]-6-deoxy-L-ascorbic acid* (**4e**): Compound **4e** (111.7 mg; 61%; m.p. = 159–160 °C) was synthesized according to the general procedure using compound **3e** (268.5 mg; 0.477 mmol); 1 M BCl_3_ (2.86 mL) and CH_2_Cl_2_ (20 mL). ^1^H NMR (300 MHz, DMSO) δ: 11.10 (s, 1H, OH), 8.62 (s, 1H, H-7), 8.48 (bs, 1H, OH), 7.81 (d, *J* = 8.5 Hz, 2H, Ph), 7.65 (d, *J* = 8.5 Hz, 2H, Ph), 5.55 (s, 1H, OH), 4.77 (d, *J* = 1.2 Hz, 1H, H-4), 4.69 (dd, *J* = 13.7 Hz, 3.7 Hz, 1H H-6), 4.42 (dd, *J* = 13.7 Hz, 9.3 Hz, 1H, H-6), 4.30–4.18 (m, 1H, H-5). ^13^C NMR (151 MHz, DMSO) δ: 170.2 (C-1), 157.3 (C-3), 145.0 (C-8), 131.8 (Ph), 130.1 (Ph-q), 127.0 (Ph), 122.8 (C-7), 120.7 (Ph-q), 118.2 (C-2), 75.5 (C-4), 67.1 (C-5), 52.9 (C-6) ppm. HRMS (ESI Q-TOF): calculated for C_14_H_12_BrN_3_O_5_ [M + H]^+^ = 382.0039; found = 382.0023. 

*Preparation of 6-deoxy-6-[4-tolyl-1,2,3-triazol-1-yl]-L-ascorbic acid* (**4f**): Compound **4f** (42.0 mg; 42%; m.p. = 152–154 °C) was synthesized according to the general procedure using compound **3f** (156.6 mg; 0.315 mmol); 1 M BCl_3_ (1.9 mL); CH_2_Cl_2_ (20 mL). ^1^H NMR (300 MHz, DMSO) δ: 11.17 (bs, 1H, OH), 8.50 (s, 1H, H-7), 8.46 (bs, 1H, OH), 7.73 (d, *J* = 8.0 Hz, 2H, Ph), 7.25 (d, *J* = 8.0 Hz, 2H, Ph), 5.54 (d, *J* = 6.7 Hz, 1H, OH), 4.77 (d, *J* = 1.0 Hz, 1H, H-4), 4.67 (dd, *J* = 13.6 Hz, 3.7 Hz, 1H, H-6), 4.41 (dd, *J* = 13.7 Hz, 9.3 Hz, 1H, H-6), 4.31–4.17 (m, 1H, H-5), 2.33 (s, 3H, CH_3_) ppm. ^13^C NMR (75 MHz, DMSO) δ: 170.3 (C-1), 152.1 (C-3), 146.1 (C-8), 137.0 (Ph-q), 129.4 (Ph), 128.1 (Ph-q), 125.0 (Ph), 122.1 (C-7), 118.3 (C-2), 75.6 (C-4), 67.1 (C-5), 52.8 (C-6), 20.7 (CH_3_) ppm. HRMS (ESI Q-TOF): calculated for C_15_H_15_N_3_O_5_ [M + H]^+^ = 318.1090; found = 318.1090.

*Preparation of 6-deoxy-6-[4-methoxyphenyl-1,2,3-triazol-1-yl]-L-ascorbic acid* (**4g**): Compound **4g** (81.8 mg; 54%; 185–187 °C) was synthesized according to the general procedure using compound **3g** (234 mg; 0.456 mmol); 1 M BCl_3_ (2.74 mL) and CH_2_Cl_2_ (20 mL). ^1^H NMR (300 MHz, DMSO) δ: 11.18 (bs, 1H, OH), 8.45 (s, 2H, H-7, OH), 7.77 (d, *J* = 8.8 Hz, 2H, Ph), 7.01 (d, *J* = 8.8 Hz, 2H, Ph), 5.53 (bs, 1H, OH), 4.76 (d, *J* = 1.5 Hz, 1H, H-4), 4.66 (dd, *J* = 13.7 Hz, 3.9 Hz, 1H, H-6), 4.40 (dd, *J* = 13.7 Hz, 9.3 Hz, 1H, H-6), 4.28–4.19 (m, 1H, H-5), 3.79 (s, 3H, OCH_3_) ppm. ^13^C NMR (75 MHz, DMSO) δ: 170.3 (C-1), 158.9 (Ph-q), 152.0 (C-3), 145.9 (C-8), 126.4 (Ph), 123.4 (Ph-q), 121.5 (C-7), 118.3 (C-2), 114.3 (Ph), 75.5 (C-4), 67.1 (C-5), 55.1 (OCH_3_), 52.7 (C-6) ppm. HRMS (ESI Q-TOF): calculated for C_34_H_45_NO_16_ [M + H]^+^ = 334.1039; found = 334.1025.

*Preparation of 6-deoxy-6-[2-hydroxyphenyl-1,2,3-triazol-1-yl]-L-ascorbic acid* (**4i**): Compound **4i** (56.5 mg; 63%; m.p. = 129–131 °C) was synthesized according to the general procedure using compound **3h** (145 mg; 0.282 mmol); 1 M BCl_3_ (1.69 mL) and CH_2_Cl_2_ (20 mL). ^1^H NMR (300 MHz, DMSO) δ: 11.13 (bs, 1H, OH), 10.20 (s, 1H, OH), 8.56–8.28 (m, 2H, H-7, OH), 8.01 (dd, *J* = 7.7 Hz, 1.5 Hz, 1H, Ph), 7.29–7.10 (m, 1H, Ph), 7.01–6.86 (m, 2H, Ph), 5.53 (bs, 1H, OH), 4.77 (d, *J* = 1.4 Hz, 1H, H-4), 4.70 (dd, *J* = 13.7 Hz, 3.7 Hz, 1H, H-6), 4.46 (dd, *J* = 13.7 Hz, 9.4 Hz, 1H, H-6), 4.25 (bs, 1H, H-5) ppm. ^13^C NMR (151 MHz, DMSO) δ: 170.4 (C-1), 153.8; 152.1 (C-3, Ph-q), 142.7 (C-8), 128.6 (Ph), 126.4 (Ph), 124.4 (C-7), 119.3 (Ph), 118.3 (C-2), 117.1 (Ph-q), 116.0 (Ph), 75.7 (C-4), 67.3 (C-5), 52.7 (C-6) ppm. HRMS (ESI Q-TOF): calculated for C_14_H_13_N_3_O_6_ [M + H]^+^ = 320.0883; found = 320.0869.

*Preparation of 6-deoxy-6-[4-(4-pentylphenyl)-1,2,3-triazol-1-yl]-L-ascorbic acid* (**4k**): Compound **4k** (62 mg; 57%; m.p. = 237–239 °C) was synthesized according to the general procedure using compound **3k** (162.0 mg; 0.293 mmol); 1 M BCl_3_ (1.8 mL) and CH_2_Cl_2_ (20 mL). ^1^H NMR (600 MHz, DMSO) δ: 11.14 (bs, 1H, OH), 8.49 (s, 1H, H-7), 8.43 (bs, 1H, OH), 7.74 (d, *J* = 8.2 Hz, 2H, Ph), 7.26 (d, *J* = 8.1 Hz, 2H, Ph), 5.53 (bs, 1H, OH), 4.76 (d, *J* = 1.6 Hz, 1H, H-4), 4.67 (dd, *J* = 13.8 Hz, 4.0 Hz, 1H, H-6), 4.41 (dd, *J* = 13.8 Hz, 9.3 Hz, 1H, H-6), 4.34–4.04 (m, 1H, H-5), 2.59 (t, *J* = 7.6 Hz, 2H, H-1′), 1.68–1.47 (m, 2H, CH_2_′), 1.44–1.18 (m, 4H, CH_2_’), 0.87 (t, *J* = 7.0 Hz, 3H, CH_3_) ppm. ^13^C NMR (151 MHz, DMSO) δ: 170.2 (C-1), 152.0 (C-3), 146.1 (C-8), 142.0 (Ph-q), 128.7 (Ph), 128.3 (Ph-q), 125.0 (Ph), 122.1 (C-7), 118.3 (C-2), 75.5 (C-4), 67.1 (C-5), 52.7 (C-6), 34.8 (C-1′), 30.8 (CH_2_’), 30.5 (CH_2_’), 21.9 (CH_2_’), 13.9 (CH_3_) ppm. HRMS (ESI Q-TOF): calculated for C_19_H_23_N_3_O_5_ [M + H]^+^ = 374.1716; found = 374.1700.

*Preparation of 6-[4-cyclopropyl-1,2,3-triazol-1-yl]-6-deoxy-L-ascorbic acid* (**4l**): Compound **4l** (80.4 mg; 78%; m.p. = 119–121 °C) was synthesized according to the general procedure using compound **3l** (172.2 mg; 0.385 mmol); 1 M BCl_3_ (2.3 mL) and CH_2_Cl_2_ (20 mL). ^1^H NMR (600 MHz, DMSO) δ: 11.13 (bs, 1H, OH), 8.43 (bs, 1H, OH), 7.80 (s, 1H, H-7), 5.44 (bs, 1H, OH), 4.68 (d, *J* = 1.2 Hz, 1H, H-4), 4.54 (dd, *J* = 13.8 Hz, 4.0 Hz, 1H, H-6), 4.29 (dd, *J* = 13.7 Hz, 9.2 Hz, 1H, H-6), 4.17–4.12 (m, 1 H, H-5), 2.11–1.44 (m, 1H, H-1′), 0.96–0.82 (m, 2H, CH_2_’), 0.78–0.59 (m, 2H, CH_2_’) ppm. ^13^C NMR (75 MHz, DMSO) δ: 170.3 (C-1), 152.1 (C-3), 148.5 (C-8), 121.8 (C-7), 118.3 (C-2), 75.5 (C-4), 67.1 (C-5), 52.5 (C-6), 7.6 (CH_2_’), 7.5 (CH_2_’), 6.5 (C-1′) ppm. HRMS (ESI Q-TOF): calculated for C_11_H_13_N_3_O_5_ [M + H]^+^ = 268.0933; found = 268.0938.

*Preparation of 6-(4-butyl-1,2,3-triazol-1-yl)-6-deoxy-L-ascorbic acid* (**4m**): Compound **4m** (36.5 mg; 47%; m.p. = 98–99 °C) was synthesized according to the general procedure using compound **3m** (127.9 mg; 0.276 mmol); 1 M BCl_3_ (1.7 mL) and CH_2_Cl_2_ (20 mL). ^1^H NMR (300 MHz, DMSO) δ: 11.14 (bs, 1H, OH), 8.45 (bs, 1H, OH), 7.82 (s, 1H, H-7), 5.44 (d, *J* = 7.0 Hz, 1H, OH), 4.68 (d, *J* = 1.5 Hz, 1H, H-4), 4.56 (dd, *J* = 13.7 Hz, 4.1 Hz, 1H, H-6), 4.32 (dd, *J* = 13.7 Hz, 9.0 Hz, 1H, H-6), 4.22–4.12 (m, 1H, H-5), 2.63–2.57 (m, 2H, H-1′), 1.65–1.49 (m, 2H, CH_2_’), 1.45–1.19 (m, 2H, CH_2_’), 0.89 (t, *J* = 7.3 Hz, 3H, CH_3_) ppm. ^13^C NMR (75 MHz, DMSO) δ: 170.3 (C-1), 152.1 (C-3), 146.5 (C-8), 122.8 (C-7), 118.3 (C-2), 75.6 (C-4), 67.2 (C-5), 52.4 (C-6), 31.1 (C-1′), 24.6 (CH_2_’), 21.7 (CH_2_’), 13.7 (CH_3_) ppm. HRMS (ESI Q-TOF): calculated for C_12_H_17_N_3_O_5_ [M + H]^+^ = 284.1246; found = 284.1250.

*Preparation of 6-(4-decyl-1,2,3-triazol-1-yl)-6-deoxy-L-ascorbic acid* (**4n**): Compound **4n** (59.1 mg; 42%; m.p. = 140–142 °C) was synthesized according to the general procedure using compound **3n** (209.1 mg; 0.382 mmol); 1 M BCl_3_ (1.6 mL) and CH_2_Cl_2_ (20 mL). ^1^H NMR (300 MHz, DMSO) δ: 11.14 (bs, 1H, OH), 8.43 (bs, 1H, OH), 7.81 (s, 1H, H-7), 5.44 (d, *J* = 5.9 Hz, OH), 4.68 (d, *J* = 1.5 Hz, 1H, H-4), 4.56 (dd, *J* = 13.7 Hz, 4.0 Hz, 1H, H-6), 4.32 (dd, *J* = 13.6 Hz, 9.1 Hz, 1H, H-6), 4.22–4.11 (m, 1H, H-5), 2.59 (t, *J* = 7.5 Hz, 2H, H-1′), 1.67–1.43 (m, 2H, H-2′), 1.36–1.17 (m, 14H, CH_2_’), 0.85 (t, *J* = 6.6 Hz, 3H, CH_3_) ppm. ^13^C NMR (75 MHz, DMSO) δ: 170.3 (C-1), 152.0 (C-3), 146.6 (C-8), 122.8 (C-7), 118.3 (C-2), 75.6 (C-4), 67.2 (C-5), 52.4 (C-6), 31.3 (C-1′), 29.0 (CH_2_’), 28.8 (CH_2_’), 28.7 (CH_2_’), 28.6 (CH_2_’), 25.0 (CH_2_’), 22.1 (CH_2_’), 143.0 (CH_3_) ppm. HRMS (ESI Q-TOF): calculated for C_18_H_29_N_3_O_5_ [M + H]^+^ = 368.2185; found = 368.2175.

*Preparation of 6-[4-(tert-butyl)-1,2,3-triazol-1-yl]-6-deoxy-L-ascorbic acid* (**4o**): Compound **4o** (64.5 mg; 48%; m.p. ≥ 250 °C) was synthesized according to the general procedure using compound **3o** (220.0 mg; 0.475 mmol); 1 M BCl_3_ (2.9 mL) and CH_2_Cl_2_ (20 mL). ^1^H NMR (300 MHz, DMSO) δ: 11.17 (bs, 1H, OH), 8.45 (bs, 1H, OH), 7.83 (s, 1H, H-7), 5.45 (bs, 1H, OH), 4.70 (d, *J* = 1.2 Hz, 1H, H-4), 4.56 (dd, *J* = 13.6 Hz, 3.8 Hz, 1H, H-6), 4.32 (dd, *J* = 13.5 Hz, 9.1 Hz, 1H, H-6), 4.25–4.12 (m, 1H, H-5), 1.27 (s, 9H, 3 × CH_3_) ppm. ^13^C NMR (75 MHz, DMSO) δ: 170.3 (C-1), 155.9 (C-8), 152.1 (C-3), 120.8 (C-7), 118.3 (C-2), 75.6 (C-4), 67.2 (C-5), 52.4 (C-6), 30.3 (3 × CH_3_), 28.9 (C-1′) ppm. HRMS (ESI Q-TOF): calculated for C_12_H_17_N_3_O_5_ [M + H]^+^ = 284.1246; found = 284.1247.

*Preparation of 6-[4-(3-chloropropyl)-1,2,3-triazol-1-yl]-6-deoxy-L-ascorbic acid* (**4p**): Compound **4p** (104.0 mg; 83%; m.p. = 86–88 °C) was synthesized according to the general procedure using compound **3p** (200.0 mg; 0.413 mmol); 1 M BCl_3_ (2.5 mL) and CH_2_Cl_2_ (20 mL). ^1^H NMR (300 MHz, DMSO) δ: 11.17 (s, 1H, OH), 8.47 (s, 1H, OH), 7.88 (s, 1H, H-7), 5.47 (d, *J* = 7.2 Hz, 1H, OH), 4.71 (d, *J* = 1.4 Hz, 1H, H-4), 4.58 (dd, *J* = 13.7 Hz, 3.9 Hz, 1H, H-6), 4.33 (dd, *J* = 13.7 Hz, 9.2 Hz, 1H, H-6), 4.26–4.11 (m, 1H, H-5), 3.68 (t, *J* = 6.5 Hz, 2H, H-3′), 2.75 (t, *J* = 7.4 Hz, 2H, H-1′), 2.08–2.01 (m, 2H, H-2′) ppm. ^13^C NMR (151 MHz, DMSO) δ: 170.3 (C-1), 152.1 (C-3), 145.2 (C-8), 123.2 (C-7), 118.3 (C-2), 75.6 (C-4), 67.2 (C-5), 52.5 (C-6), 44.7 (C-3′), 31.8 (C.-1′), 22.3 (C-2′) ppm. HRMS (ESI Q-TOF): calculated for C_11_H_14_ClN_3_O_5_ [M + H]^+^ = 304.0700; found = 304.0697.

*Preparation of 6-deoxy-6-(4-(2-hydroxyethyl)-1,2,3-triazol-1-yl)-L-ascorbic acid* (**4q**): Compound **4q** (59.5 mg; 70%; m.p. = 184–186 °C) was synthesized according to the general procedure using compound **3q** (140.3 mg; 0.311 mmol); 1 M BCl_3_ (1.9 mL) and CH_2_Cl_2_ (20 mL). ^1^H NMR (600 MHz, DMSO) δ: 11.17 (bs, 1H, OH), 8.46 (bs, 1H, OH), 7.85 (s, 1H, H-7), 5.46 (bs, 1H, OH), 4.73–4.66 (m, 2H, H-4, OH), 4.57 (dd, *J* = 13.8 Hz, 4.0 Hz, 1H, H-6), 4.33 (dd, *J* = 13.8 Hz, 9.2 Hz, 2H, H-6), 4.25–4.10 (m, 2H, H-5), 3.64–3.60 (m, 2H, H-2′), 2.76 (t, *J* = 7.0 Hz, 2H, H-1′) ppm. ^13^C NMR (151 MHz, DMSO) δ: 170.4 (C-1), 152.1 (C-3), 144.1 (C-8), 123.5 (C-7), 118.3 (C-2), 75.6 (C-4), 67.2 (C-5), 60.4 (C-2′), 52.4 (C-6), 29.2 (C-1′) ppm. HRMS (ESI Q-TOF): calculated For C_10_H_13_N_3_O_6_ [M + H]^+^ = 272.0883; found = 272.0895.

*Preparation of 6-deoxy-6-[(4-(4-methylbenzenesulphonamide)methyl-1,2,3-triazol-1-yl]-L-ascorbic acid* (**4r**): Compound **4r** (115.9 mg; 85%; m.p. = 125–127 °C) was synthesized according to the general procedure using compound **3r** (196.0 mg; 0.330 mmol); 1 M BCl_3_ (2.0 mL) and CH_2_Cl_2_ (20 mL). ^1^H NMR (600 MHz, DMSO) δ: 11.15 (bs, 1H, OH), 8.44 (bs, 1H, OH), 8.03 (t, *J* = 6.0 Hz, 1H, NH), 7.83 (s, 1H, H-7), 7.68 (d, *J* = 8.2 Hz, 2H, Ph), 7.38 (d, *J* = 8.0 Hz, 2H, Ph), 5.44 (d, *J* = 7.4 Hz, 1H, OH), 4.64 (d, *J* = 1.5 Hz, 1H, H-4), 4.55 (dd, *J* = 13.8 Hz, 4.2 Hz, 1H, H-6), 4.32 (dd, *J* = 13.8 Hz, 9.1 Hz, 1H, H-6), 4.16–4.11 (m, 1H, H-5), 4.01 (d, *J* = 6.0 Hz, 2H, CH_2_NH), 2.38 (s, 3H, CH_3_) ppm. ^13^C NMR (75 MHz, DMSO) δ: 170.3 (C-1), 152.0 (C-8), 143.13 (C-3), 142.65 (Ph-q), 137.5 (Ph-q), 129.6 (Ph), 126.6 (Ph), 124.2 (C-7), 118.3 (C-2), 75.5 (C-4), 67.2 (C-5), 52.4 (C-6), 38.1 (CH_2_NH), 21.0 (CH_3_) ppm. HRMS (ESI Q-TOF): calculated for C_16_H_18_N_4_O_7_S [M + H]^+^ = 411.0974; found = 411.0961.

*Preparation of 6-[(4-(4-chlorobenzenesulphonamide)methyl-1,2,3-triazol-1-yl]-6-deoxy-L-ascorbic acid* (**4s**): Compound **4s** (87.0 mg; 80%; m.p. = 132–134 °C) was synthesized according to the general procedure using compound **3s** (154.0 mg; 0.252 mmol); 1 M BCl_3_ (1.5 mL) and CH_2_Cl_2_ (20 mL). ^1^H NMR (300 MHz, DMSO) δ: 11.17 (bs, 1H, OH), 8.45 (bs, 1H, OH), 8.26 (t, *J* = 5.9 Hz, 1H, NH), 7.88 (s, 1H, H-7), 7.81–7.71 (m, 2H, Ph), 7.67–7.61 (m, 2H, Ph), 5.46 (d, *J* = 7.4 Hz, 1H, OH), 4.67 (d, *J* = 1.5 Hz, 1H, H-4), 4.56 (dd, *J* = 13.7 Hz, 4.0 Hz, 1H, H-6), 4.32 (dd, *J* = 13.6 Hz, 9.1 Hz, 1H, H-6), 4.18–4.09 (m, 1H, H-5), 4.06 (d, *J* = 6.0 Hz, 2H, CH_2_NH) ppm. ^13^C NMR (75 MHz, DMSO) δ: 170.1 (C-1), 152.0 (C-3), 142.9 (C-8), 139.3 (Ph-q), 137.2 (Ph-q), 129.2 (Ph), 128.5 (Ph), 124.3 (C-7), 118.4 (C-2), 75.5 (C-4), 67.2 (C-5), 52.5 (C-6), 38.0 (CH_2_NH) ppm. HRMS (ESI Q-TOF): calculated for C_15_H_5_ClN_4_O_7_S [M + H]^+^ = 431.0428; found = 431.0412.

*Preparation of 6-[(4-(2-chloro-4-fluorobenzenesulphonamide)methyl-1,2,3-triazol-1-yl]-6-deoxy-L-ascorbic acid* (**4t**): Compound **4t** (88.7 mg; 59%; m.p. = 198–200 °C) was synthesized according to the general procedure using compound **3t** (210.0 mg; 0.334 mmol); 1 M BCl_3_ (2.0 mL) and CH_2_Cl_2_ (20 mL). ^1^H NMR (300 MHz, DMSO) δ: 11.18 (bs, 1H), 8.45 (t, *J* = 5.9 Hz, 1H, NH), 7.96 (dd, *J* = 8.9, 6.0 Hz, 1H, Ph), 7.83 (s, 1H, H-7), 7.62 (dd, *J* = 8.7, 2.5 Hz, 1H, Ph), 7.36 (td, *J* = 8.6, 2.6 Hz, 1H, Ph), 4.65 (d, *J* = 1.3 Hz, 1H, H-4), 4.54 (dd, *J* = 13.8 Hz, 4.0 Hz, 1H, H-6), 4.30 (dd, *J* = 13.7 Hz, 9.1 Hz, 1H, H-6), 4.17 (d, *J* = 5.9 Hz, 2H, CH_2_NH), 4.14–4.06 (m, 1H, H-5) ppm. ^13^C NMR (75 MHz, DMSO) δ: 170.2 (C-1), 165.3; 161.9 (d, ^1^*J_CF_* = 254.3 Hz, Ph-4), 151.9 (C-3), 142.9 (C-8), 134.8; 137.7 (d, ^4^*J_CF_* = 3.6 Hz, Ph-1), 132.8; 132.6 (d, ^3^*J_CF_* = 10.1 Hz, Ph-6), 132.6; 132.4 (d, ^3^*J_CF_* = 10.1 Hz, Ph-2), 124.2 (C-7), 119.2; 118.9 (d, ^2^*J_CF_* = 25.9 Hz, Ph), 118.3 (C-2), 114.8; 114.5 (d, ^2^*J_CF_* = 21.8 Hz, Ph), 75.5 (C-4), 67.2 (C-5), 52.4 (C-6), 37.8 (CH_2_NH) ppm. HRMS (ESI Q-TOF): calculated for C_15_H_14_ClFN_4_O_7_S [M + H]^+^ = 449.0334; found = 449.0326.

*Preparation of 6-[4-((tert-butyl 1-carboxylate)-4-(methylthiocarbothionyl)piperazine))-1,2,3-triazol-1-yl]-6-deoxy-L-ascorbic acid* (**4v**): Compound **4v** (31.2 mg; 35%; m.p. ≥ 250 °C) was synthesized according to the general procedure using compound **3u** (147.7 mg; 0.221 mmol); 1 M BCl_3_ (1.3 mL) and CH_2_Cl_2_ (15 mL). ^1^H NMR (600 MHz, DMSO) δ: 9.74 (bs, 1H, NH), 8.52 (bs, 1H, OH), 8.08 (s, 1H, H-7), 5.46 (bs, 1H, OH), 4.71 (d, *J* = 1.5 Hz, 1H, H-4), 4.67–4.54 (m, 3H, H-6, CH_2_S), 4.39–4.11 (m, 4H, H-6, H-5, 2 × CH_2_), 3.2–3.1 (m, 4H, 2 × CH_2_) ppm. ^13^C NMR (151 MHz, DMSO) δ: 195.8 (CS), 170.1 (C-1), 151.7 (C-3), 141.2 (C-8), 124.8 (C-7), 118.3 (C-2), 75.6 (C-4), 67.2 (C-5), 52.7 (C-6), 42.3 (CH_2_’), 31.9 (CH_2_S) ppm. HRMS (ESI Q-TOF): calculated for C_14_H_19_N_5_O_5_S_2_ [M + H]^+^ = 402.0906; found = 402.0891.

*Preparation of 4,5-didehydro-5,6-dideoxy-(4-(3,5-di-(trifluoromethyl)phenyl)-1,2,3-triazol-1-yl)-L-ascorbic acid* (**7c**): Compound **7c** (72.1 mg; 52%; m.p. ≥ 200 °C) was synthesized according to the general procedure using compound **6c** (200 mg; 0.33 mmol); 1 M BCl_3_ (1.98 mL) and CH_2_Cl_2_ (20 mL). ^1^H-NMR (300 MHz, DMSO) δ: 11.21 (s, 1H, OH), 9.95 (s, 1H, OH), 9.01 (s, 1H, H-7), 8.54 (s, 2H, Ph-2, Ph-6), 8.07 (s, 1H, Ph-4), 5.61 (t, *J* = 7.6 Hz, 1H, H-5), 5.30 (d, *J* = 7.7 Hz, 2H, H-6) ppm. ^13^C-NMR (151 MHz, DMSO) δ: 164.6 (C-1), 145.8 (C-3), 143.8 (C-4), 142.9 (C-8), 133.2 (Ph-1), 131.3; 131.1; 130.9; 130.7 (q, ^2^*J_CF_* = 33.0 Hz, Ph-3, Ph-5), 125.9; 124.1; 122.3 (m, *^1^J_CF_* = 272.8 Hz, CF_3_), 125.3 (Ph-2, Ph-6), 123.3 (C-7), 122.1 (C-2), 121.1 (Ph-4), 98.2 (C-5), 44.5 (C-6) ppm. HRMS (ESI Q-TOF): calculated for C_16_H_9_F_6_N_3_O_4_ [M + H]^+^ = 422.0576; found = 422.0580.

*Preparation of 4,5-didehydro-5,6-dideoxy-(4-(3,5-difluoromethyl)phenyl)-1,2,3-triazol-1-yl)-L-ascorbic acid* (**7d**): Compound **7d** (61.1 mg; 48%; m.p. ≥ 200 °C) was synthesized according to the general procedure using compound **6d** (200 mg; 0,39 mmol); 1 M BCl_3_ (2.27 mL) and CH_2_Cl_2_ (20 mL): Compound **7d** was isolated as white powder. ^1^H-NMR (600 MHz, DMSO) δ: 11.31 (s, 1H, OH), 9.83 (s, 1H, OH), 8.75 (s, 1H, H-7), 7.59 (d, *J* = 6.6 Hz, 2H, Ph-2, Ph-6), 7.31–7.03 (m, 1H, Ph-4), 5.58 (t, *J* = 7.7 Hz, 1H, H-5), 5.26 (d, *J* = 7.7 Hz, 2H, H-6) ppm. ^13^C-NMR (151 MHz, DMSO) δ: 164.6 (C-1), 163.7; 163.6; 162.1; 162.0 (dd, ^1^*J_CF_* = 245.6 Hz, ^3^*J_CF_* = 13.7 Hz, Ph-3, Ph-5), 145.7 (C-3), 144.6 (C-8), 142.9 (C-4), 134.2; 134.2; 134.1 (t, *J* = 10.5 Hz, Ph-1), 122.7 (C-7), 122.1 (C-2), 108.2; 108.1; 108.0; 108.0 (dd, ^2^*J_CF_* = 21.1 Hz, ^4^*J_CF_* = 5.5 Hz, Ph-2, Ph-6), 103.2; 103.1; 102.9 (t, ^2^*J* = 26.0 Hz, Ph-4), 98.4 (C-5), 44.5 (C-6) ppm. HRMS (ESI Q-TOF): calculated for C_14_H_9_F_2_N_3_O_4_ [M + H]^+^ = 322.0639; found = 322.0642.

*Preparation of 6-[4-(4-bromophenyl)-1,2,3-triazole-1-yl]-4,5-didehydro-5,6-dideoxy-L-ascorbic acid* (**7e**): Compound **7e** (24 mg; 21%; m.p. = 219–222 °C) was synthesized according to the general procedure using compound **6e** (177 mg; 0.324 mmol); 1 M BCl_3_ (1.94 mL) and CH_2_Cl_2_ (20 mL). ^1^H NMR (300 MHz, DMSO) δ: 10.48 (s, OH), 8.67 (s, 1H, H-7), 7.82 (d, *J* = 8.5 Hz, 2H, Ph), 7.64 (d, *J* = 8.6 Hz, 2H, Ph), 5.58 (t, *J* = 7.5 Hz, 1H, H-5), 5.25 (d, *J* = 7.5 Hz, 2H, H-6). ^13^C NMR (75 MHz, DMSO-d6) δ: 164.7 (C-1), 145.5 (C-3), 143.0 (C-8, C-4), 131.8 (Ph), 129.9 (Ph-q), 127.1 (Ph), 122.0 (C-7), 121.8 (C-2), 120.8 (Ph-q), 98.7 (C-5), 44.4 (C-6) ppm. HRMS (ESI Q-TOF): calculated for C_14_H_10_BrN_3_O_4_ [M + H]^+^ = 363.9933; found = 363.9915.

*Preparation of 6-[4-tolyl-1,2,3-triazole-1-yl]-4,5-didehydro-5,6-dideoxy-L-ascorbic acid* (**7f**): Compound **7f** (20 mg; 15%; m.p. = 188–192 °C) was synthesized according to the general procedure using compound **6f** (203 mg; 0.407 mmol); 1 M BCl_3_ (2.45 mL) and CH_2_Cl_2_ (20 mL). ^1^H NMR (600 MHz, DMSO) δ: 11.40 (s, 1H, OH), 9.78 (s, 1H, OH), 8.56 (s, 1H, H-7), 7.74 (d, *J* = 8.0 Hz, 2H, Ph), 7.25 (d, *J* = 7.9 Hz, 2H, Ph), 5.59 (t, *J* = 7.6 Hz, 1H, H-5), 5.24 (d, *J* = 7.6 Hz, 2H, H-6), 2.33 (s, 3H, CH_3_) ppm. ^13^C NMR (151 MHz, DMSO) δ: 164.7 (C-1), 146.5 (C-3), 145.3 (C-4), 143.0 (C-8), 137.2 (Ph-q), 129.4 (Ph), 127.9 (Ph-q), 125.0 (Ph), 121.9 (C-7), 120.9 (C-2), 98.8 (C-5), 44.3 (C-6), 20.7 (CH_3_) ppm. HRMS (ESI Q-TOF): calculated for C_15_H_13_N_3_O_4_ [M + H]^+^ = 300.0984; found 300.0974.

*Preparation of 4,5-didehydro-5,6-dideoxy-6-[4-(4-methoxyphenyl)-1,2,3-triazole-1-yl]-L-ascorbic acid* (**7g**): Compound **7g** (58.1 mg; 28%; m.p. = 198–199 °C) was synthesized according to the general procedure using compound **6g** (328 mg; 0.662 mmol); 1 M BCl_3_ (3.96 mL) and CH_2_Cl_2_ (25 mL). ^1^H NMR (600 MHz, DMSO) δ: 11.05 (bs, 1H, OH), 9.95 (bs, 1H, OH), 8.49 (s, 1H, H-7), 7.78 (d, *J* = 8.7 Hz, 2H, Ph), 7.01 (d, *J* = 8.7 Hz, 2H, Ph), 5.58 (t, *J* = 7.6 Hz, 1H, H-5), 5.23 (d, *J* = 7.6 Hz, 2H, H-6) ppm. ^13^C NMR (151 MHz, DMSO) δ: 164.7 (C-1), 159.0 (Ph-q), 146.5 (C-3), 145.3 (C-8), 126.5 (Ph), 123.3 (Ph-q), 121.9 (C-2), 120.4 (C-7), 114.3 (Ph), 99.0 (C-5), 55.1 (OCH_3_), 44.3 (C-6) ppm. HRMS (ESI Q-TOF): calculated for C_15_H_13_N_3_O_5_ [M + H]^+^ = 316.0933; found = 316.0930.

*Preparation of 4,5-didehydro-5,6-dideoxy-6-[4-(2-hydroxyphenyl)-1,2,3-triazole-1-yl]-L-ascorbic acid* (**7i**): Compound **7i** (64.2 mg; 56%; m.p. = 167–169 °C) was synthesized according to the general procedure using compound **6h** (188 mg; 0.379 mmol); 1 M BCl_3_ (2.28 mL) and CH_2_Cl_2_ (20 mL). ^1^H NMR (300 MHz, DMSO) δ: 11.13 (bs, 1H, OH), 10.15 (s, 1H, OH), 9.74 (bs, 1H, OH), 8.41 (s, 1H, H-7), 7.97 (dd, *J* = 7.7 Hz, 1.7 Hz, 1H, Ph), 7.43–7.05 (m, 1H, Ph), 6.99–6.78 (m, 2H, Ph), 5.58 (t, *J* = 7.5 Hz, 1H, H-5), 5.25 (d, *J* = 7.5 Hz, 2H, H-6) ppm. 13C NMR (75 MHz, DMSO) δ: 164.6 (C-1), 153.9 (Ph-q), 145.1 (C-3), 143.1 (C-8), 143.0 (C-4), 128.7 (Ph), 126.5 (Ph), 123.1 (C-7), 121.9 (C-2), 119.2 (Ph), 116.9 (Ph-q), 115.9 (Ph), 99.3 (C-5), 44.2 (C-6) ppm. HRMS (ESI Q-TOF): calculated for C_14_H_11_N_3_O_5_ [M + H]^+^ = 302.0777; found = 302.0782.

*Preparation of 4,5-didehydro-5,6-dideoxy-6-[4-(3-hydroxyphenyl)-1,2,3-triazole-1-yl]-L-ascorbic acid* (**7j**): Compound **7j** (45.3 mg; 41%; m.p. = 155–157 °C) was synthesized according to the general procedure using compound **6j** (175 mg; 0.363 mmol); 1 M BCl_3_ (2.18 mL) and CH_2_Cl_2_ (20 mL). ^1^H NMR (600 MHz, DMSO) δ: 11.33 (s, 1H, OH), 9.90 (s, 1H, OH), 9.55 (s, 1H, OH), 8.55 (s, 1H, H-7), 7.54–7.02 (m, 3H, Ph), 6.72 (d, *J* = 7.0 Hz, 1H, Ph), 5.59 (t, *J* = 7.6 Hz, 1H, H-5), 5.23 (d, *J* = 7.6 Hz, 2H, H-6) ppm. ^13^C NMR (151 MHz, DMSO) δ: 164.7 (C-1), 157.8 (Ph-q), 146.7 (C-3), 145.3 (C-4), 143.0 (C-8), 131.9 (Ph-q), 130.0 (Ph), 122.0 (C-7), 121.4 (C-2), 116.1 (Ph), 114.9 (Ph), 111.9 (Ph), 99.0 (C-5), 44.4 (C-6) ppm. HRMS (ESI Q-TOF): calculated for C_14_H_11_N_3_O_5_ [M + H]^+^ = 302.0777; found = 302.0765.

*Preparation of 4,5-didehydro-5,6-dideoxy-6-[4-(4-pentylphenyl)-1,2,3-triazole-1-yl]-L-ascorbic acid* (**7k**): Compound **7k** (31 mg; 24%; m.p. = 230–232 °C) was synthesized according to the general procedure using compound **6k** (195 mg; 0.364 mmol); 1 M BCl_3_ (2.18 mL) and CH_2_Cl_2_ (20 mL). ^1^H NMR (300 MHz, DMSO) δ: 11.10 (bs, 1 H, OH), 9.98 (bs, 1H, OH), 8.55 (s, 1H, H-7), 7.75 (d, *J* = 8.1 Hz, 2H, Ph), 7.26 (d, *J* = 8.1 Hz, 2H, Ph), 5.59 (t, *J* = 7.6 Hz, 1H, H-5), 5.24 (d, *J* = 7.6 Hz, 1H, H-6), 2.59 (t, *J* = 7.6 Hz, 2H, H-1′), 1.65–1.51 (m, 2H, CH_2_), 1.38–1.17 (m, 4H, CH_2_), 0.86 (t, *J* = 6.8 Hz, 3H, CH_3_) ppm. ^13^C NMR (75 MHz, DMSO) δ: 164.7 (C-1), 146.6 (C-3), 145.3 (C-4), 143.0 (C-8), 142.1 (Ph-q), 128.8 (Ph), 128.1 (Ph-q), 125.1 (Ph), 122.0 (C-2), 121.0 (C-7), 99.0 (C-5), 44.3 (C-6), 34.8 (C-1′), 30.9 (C-2′), 30.5 (CH_2_), 21.9 (CH_2_), 13.9 (CH_3_) ppm. HRMS (ESI Q-TOF): calculated for C_19_H_21_N_3_O_4_ [M + H]^+^ = 356.1610; found = 356.1598.

*Preparation of 6-[4-cyclopropyl-1,2,3-triazole-1-yl]-4,5-didehydro-5,6-dideoxy-L-ascorbic acid* (**7l**): Compound **7l** (52.5 mg; 53%; m.p. = 176–178 °C) was synthesized according to the general procedure using compound **6l** (170 mg; 0.396 mmol); 1 M BCl_3_ (2.38 mL) and CH_2_Cl_2_ (20 mL). ^1^H NMR (300 MHz, DMSO) δ: 11.10 (bs, 1H, OH), 9.88 (bs, 1H, OH), 7.83 (s, 1H, H-7), 5.50 (t, *J* = 7.5 Hz, 1H, H-5), 5.11 (d, *J* = 7.6 Hz, 2H, H-6), 1.98–1.86 (m, 1H, CH), 0.95–0.84 (m, 2H, CH_2_), 0.73–0.65 (m, 2H, CH_2_) ppm. ^13^C NMR (75 MHz, DMSO) δ: 164.6 (C-1), 149.2 (C-8), 145.0 (C-3), 143.0 (C-4), 121.9 (C-2), 120.6 (C-7), 99.2 (c-5), 44.0 (C-6), 7.5 (CH_2_), 6.4 (CH) ppm. HRMS (ESI Q-TOF): calculated for C_11_H_11_N_3_O_4_ [M + H] ^+^ = 250.0828; found = 250.0826.

*Preparation of 6-[4-butyl-1,2,3-triazole-1-yl]-4,5-didehydro-5,6-dideoxy-L-ascorbic acid* (**7m**): Compound **7m** (66.8 mg; 47%; m.p. = 180–183 °C) was synthesized according to the general procedure using compound **6m** (237 mg; 0.532 mmol); 1 M BCl_3_ (3.19 mL) and CH_2_Cl_2_ (20 mL). ^1^H NMR (600 MHz, DMSO) δ: 11.32 (s, 1H, OH), 9.76 (s, 1H, OH), 7.88 (s, 1H, H-7), 5.52 (t, *J* = 7.6 Hz, 1H, H-5), 5.14 (d, *J* = 7.6 Hz, 2H, H-6), 2.60 (t, *J* = 7.6 Hz, 2H, H-1′), 1.68–1.42 (m, 2H, CH_2_), 1.41–1.26 (m, 2H, CH_2_), 0.89 (t, *J* = 7.4 Hz, 3H, CH_3_) ppm. ^13^C NMR (151 MHz, DMSO) δ: 164.7 (C-1), 147.2; 145.0; 143.1 (C-3, C-4, C-8), 121.9 (C-2), 121.8 (C-7), 99.4 (C-5), 44.0 (C-6), 31.1 (C-1′), 24.7 (CH_2_), 21.7 (CH_2_), 13.7 (CH_3_) ppm.

*Preparation of 6-(4-decyl-1,2,3-triazol-1-il)-4,5-didehydro-5,6-dideoxy-L-ascorbic acid* (**7n**): Compound **7n** (55 mg; 42%; m.p. = 169–171 °C) was synthesized according to the general procedure using compound **6n** (200 mg; 0.38 mmol); 1 M BCl_3_ (2.28 mL) and CH_2_Cl_2_ (20 mL). ^1^H-NMR (600 MHz, DMSO) δ: 11.28 (s, 1H, OH), 9.73 (s, 1H, OH), 7.86 (s, 1H, H-7), 5.52 (t, 1H, *J* = 7.5 Hz, H-5), 5.14 (s, 2H, H-6), 2.60–2.57 (m, 2H, H-1′), 1.58–1.56 (m, 2H, H-2′), 1.33–1.20 (m, 14H, CH_2_), 0.84 (t, *J* = 7.0 Hz, 3H, CH_3_) ppm. ^13^C-NMR (151 MHz, DMSO) δ: 164.6 (C-1), 147.2 (C-3), 144.9 (C-4), 143.0 (C-8), 121.9 (C-2), 121.7 (C-7), 99.3 (C-5), 43.9 (C-6), 31.2 (C-1′), 28.9 (CH_2_’), 28.7 (CH_2_’), 28.6 (CH_2_’), 28.5 (CH_2_’), 24.9 (CH_2_’), 22.1 (CH_2_’), 13.9 (CH_3_) ppm. HRMS (ESI Q-TOF): calculated for C_18_H_27_N_3_O_4_ [M + H] ^+^ = 350.2080; found = 350.2086.

*Preparation of 6-[4-tert-butyl-1,2,3-triazole-1-yl]-4,5-didehydro-5,6-dideoxy-L-ascorbic acid* (**7o**): Compound **7o** (30 mg; 24%; m.p. = 182–184 °C) was synthesized according to the general procedure using compound **6o** (203 mg; 0.455 mmol); 1 M BCl_3_ (2.72 mL) and CH_2_Cl_2_ (20 mL). ^1^H NMR (300 MHz, DMSO) δ: 11.31–9.54 (bs, 2H, OH × 2), 7.88 (s, 1H, H-7), 5.53 (t, *J* = 7.5 Hz, 1H, H-5), 5.14 (d, *J* = 7.5 Hz, 2H, H-6), 1.27 (s, 9H, CH_3_ × 3). ^13^C NMR (75 MHz, DMSO) δ: 164.6 (C-1), 144.9 (C-3), 143.0 (C-4), 121.9 (C-2), 119.7 (C-7), 99.3 (C-5), 43.9 (C-6), 30.2 (C-1′), 30.2 (CH_3_) ppm. HRMS (ESI Q-TOF): calculated for C_12_H_15_N_3_O_4_ [M + H] ^+^ = 266.1141; found = 266.1129.

*Preparation of 6-[4-chloropropyl-1,2,3-triazole-1-yl]-4,5-didehydro-5,6-dideoxy-L-ascorbic acid* (**7p**): Compound **7p** (53.2 mg; 38%; m.p. = 160–162 °C) was synthesized according to the general procedure using compound **6p** (230 mg; 0.494 mmol); 1 M BCl_3_ (2.96 mL); CH_2_Cl_2_ (20 mL). ^1^H NMR (300 MHz, DMSO) δ: 11.18–9.67 (bs, 2H, OH × 2), 7.93 (s, 1H, H-7), 5.53 (t, *J* = 7.5 Hz, 1H, H-5), 5.15 (d, *J* = 7.5 Hz, 2H, H-6), 3.67 (t, *J* = 6.5 Hz, 2H, H-3′), 2.82–2.66 (m, 2H, H-1′), 2.10–1.98 (m, 2H, H-2′) ppm. ^13^C NMR (75 MHz, DMSO) δ: 164.6 (C-1), 145.7; 145.0; 143.0 (C-3, C-4, C-8), 122.1 (C-7), 121.9 (C-2), 99.2 (C-5), 44.6 (C-1′), 44.0 (C-6), 31.7 (C-1′), 22.3 (C-3′) ppm. HRMS (ESI Q-TOF): calculated for C_11_H_12_ClN_3_O_4_ [M + H] ^+^ = 286.0595; found = 286.0599.

*Preparation of 6-[4-(2-hydroxyethyl)-1,2,3-triazole-1-yl]-4,5-didehydro-5,6-dideoxy-L-ascorbic cid* (**7q**): Compound **7q** (81.2 mg; 70%; m.p. = 172–173 °C) was synthesized according to the general procedure using compound **6q** (200 mg; 0.461 mmol); 1 M BCl_3_ (2.77 mL); CH_2_Cl_2_ (20 mL). ^1^H NMR (300 MHz, DMSO) δ: 11.21 (bs, 1H, OH), 9.87 (bs, 1H, OH), 7.87 (s, 1H, H-7), 5.52 (t, *J* = 7.6 Hz, 1H, H-5), 5.15 (d, *J* = 7.6 Hz, 2H, H-6), 4.65 (bs, 1H, OH), 3.62 (t, *J* = 6.9 Hz, 2H, H-2′), 2.76 (t, *J* = 6.9 Hz, H-1′) ppm. ^13^C NMR (75 MHz, DMSO) δ: 164.6 (C-1), 145.0 (C-3), 144.7 (C-8), 143.0 (C-4), 122.3 (C-7), 121.9 (C-2), 99.3 (C-5), 60.3 (C-2′), 43.9 (C-6), 29.1 (C-1′) ppm. HRMS (ESI Q-TOF): calculated for C_10_H_11_N_3_O_5_ [M + H] ^+^ = 254.0777; found = 254.0770.

### 3.2. Biological Evaluation

#### 3.2.1. DPPH Assay

Antioxidant capacity of selected compounds was assessed by 2,2-diphenyl-1-picrylhydrazyl assay (DPPH, Sigma Aldrich, Darmstadt, Germany), in which the antioxidant activity of compounds is measured by the reaction between a stable radical of 1,1-diphenyl-2-picylhydrazil (DPPH^•^) and a selected compound according to the manufacturer recommendations. Briefly, compounds **4b–4e**, **4f–4h**, **4k**, **4l**, **4o**, **4p–4s**, **4t**, **4v**, **7c–7f**, **7i–7n**, **7q**, **7p**, and **7o** were tested at 0.06, 0.12, 0.18, 0.23, 0.25, and 0.28 mM. L-ascorbic acid was used as the reference compound. Absorbance was measured 5 and 10 min after incubation of the compounds with the DPPH reagent. The reduction of DPPH with selected compounds was monitored by measuring the absorbance at a wavelength of 517 nm on the monochromator (Infinite M200 PRO, TECAN, Männedorf, Switzerland).

#### 3.2.2. In Silico Approaches—DFT Calculations and PCA Analysis

The gas-phase (g) reaction parameters for radical scavenging models: bond dissociation enthalpy (BDE), basicity, and electron transfer enthalpy (ETE), and their aqueous free energy (FE) counterparts BDFE, pK_a_, and ETFE, respectively, were calculated by using thermochemical cycles [42] in the density functional theory (DFT) model (U)M052X/6-311++G** implemented in Gaussian 09 [57,58]. The gas-phase equilibrium geometries were optimized for species in neutral and anion closed-shell singlet and neutral monoradical and radical anion open-shell doublet ground electronic states. The minima were confirmed by absence of imaginary vibrational frequencies. Calculations were done for the compounds with a free C5OH group in the 4R,5S-configuration as a starting L-ASA (series **4**). The compounds with a C4=C5 double bond were determined in Z-configuration (series **7**) (Scheme 1) [59]. The free energies of hydratation ΔG*_hyd_ were determined at the gas phase geometries by using the SMD method [60]. The gas-phase thermodynamic reaction parameters were calculated in terms of the sums of electronic and thermal enthalpies or free energies estimated at temperature of 298.15 K and pressure of 1 atm. For the electron and the proton, values reported in the reference [61] were used. Regarding free energies of hydratation ΔG°_hyd_(H^•^), ΔG°_hyd_ (H^+^), and ΔG°_hyd_ (e^−^), the experimental values of 27.8 kJ/mol [62], −1104.62 kJ/mol [63] and −148.5 kJ/mol [64] were used, respectively.

The PCA analyses were done by R package princomp [65]. The values of partition n-octanol/water coefficients—clogP were calculated by DataWarrior by using SMILES as inputs [44].

#### 3.2.3. Cell Culturing

Human tumor cell lines HeLa (cervical carcinoma), SW620 (colorectal adenocarcinoma, metastatic), A549 (lung adenocarcinoma), MCF−7 (breast adenocarcinoma), CFPAC-1 (pancreatic adenocarcinoma, metastatic), HCT-116 (colorectal carcinoma), and HepG2 (hepatocellular carcinoma), and normal cell lines HFF-1 (foreskin fibroblasts) and WI-38 (immortalized human lung fibroblast), were from the US American Type Culture Collection (ATCC, Manassas, VA, USA). Cells were cultured in a liquid nutrient medium (Dulbecco’s Modified Eagle Medium, LONZA, Basel, Switzerland) supplemented with 10% fetal bovine serum (FBS, Gibco, Grand Island, NY, USA), 2 mM L-glutamine (GIBCO, Grand Island, NY, USA), 100 μg/mL penicillin, and 100 μg/mL streptomycin (LONZA, Basel, Switzerland), in an incubator (NÜVE, Ankara, Turkey), at a humid atmosphere with 5% CO_2_ at 37 °C. Trypsin/EDTA Solution (Lonza, Basel, Switzerland), was used for cell passaging and cell detachment.

#### 3.2.4. Proliferation Assay- MTT Assay

Adherent cells were seeded on 96 well microtiter plates (Falcon, New York, NY, USA) at a seeding density of 5000 cells per well, added in 150 μL of cell suspension in a nutrient medium. 24 h after seeding, cells were treated with compound **4b–4g**, **4i**, **4k–4t**, **4v**, **7c–7g**, and **7i–7q** dilutions in the range of 0.01 μM to 100 μM. L-ascoric acid (Sigma Aldrich, Darmstadt, Germany) and Carboxyamidotriazole (Sigma Aldrich, Darmstadt, Germany) were used as control compounds. Incubation of the cells with the compounds were performed for 72 h in a 5% CO_2_, in humid atmosphere at 37 °C. After incubation, nutrient medium was removed and 40 μL of the MTT reagent (3-(4-dimethylthiazol-2-yl)-2,5-diphenyltetrazole bromide (Invitrogen, Carlsbad, CA, USA) was added to each well. 3 h after incubation, 160 μL of DMSO (VWR CHEMICALS, Radnor, PA, USA) were added to each well to dissolve the crystals of purple formazane. The absorbance was measured at 570 nm (TECAN SUNRISE Microtiter plate reader (Männedorf, Switzerland). The experimentally measured optical densities (OD) were converted to the PG (percentage of growth), using the formulas according to the protocol assumed by NIH (National Institute of Health). The IC_50_ values (concentration causing 50% cell growth inhibition) for each compound were calculated by regression analysis of the survival count curve of the concentrations of the tested compounds. The results were processed in Excel 2010 (Microsoft, Redmond, WA, USA).

#### 3.2.5. Apoptosis Detection—Annexin V Assay

A total of 2 × 10^4^ MCF-7 cells were seeded into 8-well Nunc Lab-Tek II Chamber Slide system (Thermo Fischer Scientific, Waltham, MA, USA), and treated with compound **4e** at 1 × IC_50_ and 2 × IC_50_ for 24 and 48 h. The assay (Annexin-V-FLUOS Staining kit, Roche Applied Science, Penzberg, Germany) was preformed according to the manufacturer protocol. Slides were analyzed by fluorescence microscopy (Zeiss Axio Observer Z1 Inverted Phase Contrast Fluorescent Microscope, Jena, Germany)).

#### 3.2.6. Western Blot Analysis

MCF-7 cells were seeded in Petri dishes, at 1 × 10^6^ cells, and treated with compound **4e** at IC_50_ 6.72 µM for 24 and 48 h. Protein lysates were prepared using RIPA buffer containing cocktail of protease and phosphatase inhibitors (Roche, Basel, Switzerland). Amounts of 50 μg of proteins were resolved on 12% SDS polyacrylamide gels using the Mini-protean cell (Bio-Rad, Hercules, CA, USA). The membranes were incubated with primary antibodies raised against hydroxy-HIF-1α (Pro564) (HIF-1α, 1:1000, rabbit mAb, Cell Signaling Technology, NL) and NOS2 (1:500, mouse mAb, SantaCruzBiotechnology, Dallas, TX, USA) at 4 °C overnight. A secondary antibody linked to anti-mouse (1:1000, Dako, Santa Clara, CA, USA) was used. The signal was visualized by the Western Lightening Chemiluminescence Reagent Plus Kit (Perkin Elmer, Massachusetts, MA, USA) on the ImageQuant LAS500 (GE Healthcare, Chicago, IL, USA), and as a loading control, α-tubulin (1:1000, mouse mAb, Sigma, Sigma Aldrich, Darmstadt, Germany) was used. The signal intensities of bands were normalized and compared in Quantity One software (Bio-Rad, Hercules, CA, USA). Differences in protein relative expression status obtained by western blot analysis were analyzed by two-tailed, paired *t*-tests (*p* < 0.05) in the Statistica software package (v.12.0).

#### 3.2.7. Seahorse XF Cell Myto Stress Test

The Seahorse XF Cell Mito Stress Test was used for measurements of mitochondrial respiration by direct assessment of the oxygen consumption rate (OCR), of treated and untreated cells on the Seahorse XFe24 Analyzer, (Agilent, Santa Clara, CA, USA). Key parameters of the mitochondrial function were measured by direct assessment of the OCR, by use of the Mito Stress Test (Agilent, Agilent, Santa Clara, CA, USA). The Mito Stress Test uses modulators of respiration that target components of the electron transport chain in the mitochondria. The compounds added to the reaction were: oligomycin, mitochondrial oxidative phosphorylation uncoupler carbonyl cyanide-4-(trifluoromethoxy)phenylhydrazone (FCCP), and the mix of rotenone and antymicin A. These were directly injected to the cell growth medium containing cells treated with selected L-ASA derivatives to determine the production of ATP, respiration at maximal level, and the non-mitochondrial respiration. Measured parameters were used for calculation of the proton leakage, spare respiratory capacity, and basal respiration. The assay was performed according to the manufacturer’s instructions (SeaHorse—Mito Stress Test: Agilent, Santa Clara, CA, USA). Briefly, selected compound **4e** was tested due to its selective antiproliferative effect on MCF-7 cells. MCF-7 cells were treated for 24 h with compound **4e** at 3 different concentrations (at 12.72 µM, 25.44 µM and 50.88 µM) while the cells HFF-1 were treated for 24 h with compound **4e** at concentrations 50 µM, 100 µM, and 200 µM. L-ascorbic acid, was used as control compound and tested at concentrations 50, 100, and 150 mM (same as those used for DPPH assay) on MCF-7 and HFF-1 cells. The experiment was performed with biological duplicates.

## 4. Conclusions

With the aim to find compounds with superior antioxidant and antiproliferative activity compared to L-ASA, two novel classes of L-ASA derivatives, 6-(1,2,3-triazolyl)-6-deoxy-L-ASA (series **4**) with the C4-C5(OH) group, and their analogues (series **7**) with the C4=C5 double bond were synthesized. The assessment of the antioxidant activity by DPPH^•^ radical scavenging assay showed that the majority of the 4,5-unsaturated L-ASA derivatives (series **7**) showed better antioxidant activity compared to their saturated analogues (series **4**). Moreover, the introduction of an additional hydroxyl group at 1,2,3-triazole of the 4,5-unsaturated L-ASA derivatives (in **7i**, **7j**, and **7q**) resulted in improvement of the radical scavenging activity. An explanation of the observed differences in the DPPH^•^ radical scavenging activities of the L-ASA−triazole conjugates has been obtained using in silico analysis, which showed that the triazole conjugates have an analogous radical scavenging mechanism to L-ASA and that the most significant difference between saturated C4-C5(OH) and unsaturated C4=C5 derivatives is in acidity of C2-OH group. The active radical scavenging center in L-ASA derivatives with a C4-C5(OH) fragment is the C3-OH group, while for majority unsaturated C4=C5 derivatives, the radical scavenging center can be ascribed to C2-OH group. Furthermore, the antiproliferative evaluation on seven malignant tumor cell lines proved that the linker connecting the lactone ring and the 1,2,3-triazole moiety has an influence on the antitumor activity. Hence, 1,2,3-triazole L-ASA derivatives with a hydroxyethylene C4-C5(OH) unit showed better growth-inhibition effects on tumor cells compared to their 4,5-unsaturated C4=C5 analogues. Additionally, unsubstituted 1,2,3-triazolyl derivative **4b** was not cytotoxic toward any of the tested cell lines, which shows that C-4 substitution at the 1,2,3-triazole moiety has an impact on growth-inhibition related effects. Compound **4e** with a *p*-bromophenyl and **4k** with a *p*-pentylphenyl-substituted 1,2,3-triazole L-ASA both exerted a selective cytostatic effect on MCF-7 cells (IC_50_ = 6.72 μM; IC_50_ = 26.91 μM, respectively). Western blot analysis of the relative expression of HIF-1α and NOS2 proteins showed that compound **4e** strongly increased the expression of hydroxylated HIF-1α and somewhat decreased the expression of NOS2, indicating its role in the HIF-1-triggered response of MCF-7 cells to hypoxia. Cellular-metabolism analysis showed that compound **4e** increased the basal respiration and ATP production of MCF-7 cells, while it did not affect the basal cell respiration and ATP production of HFF-1 cells, which is in agreement with MTT-results, in which **4e** showed no cytotoxicity on HFF-1 cells. Overall, 4-substituted 1,2,3-triazole L-ASA conjugates were identified as a promising chemical entity that affected the HIF-1α signaling pathway, and further structure optimization of the saturated C4-C5(OH) derivatives could lead to a more potent and selective growth-inhibition effect on breast adenocarcinoma (MCF-7) cells.

## Figures and Tables

**Table 1:** The scavenging activity of L-ascorbic acid derivatives assessed by DPPH test. Results are presented as IC_50_ values (mM) of DPPH^•^ inhibition after 5 and 10 min treatments.

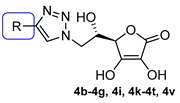				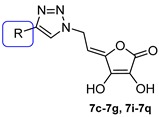			
Comp.	R	IC_50_ (mM)^a^		Comp.	R	IC_50_ (mM)^a^	
		Treatm. 5 min	Treatm. 10 min			Treatm. 5 min	Treatm. 10 min
L-ASA		0.18 ± 0.06	0.17 ± 0.05	**4t**	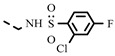	0.22 ± 0.08	0.12 ± 0.02
**4b**	H	0.13 ± 0.05	0.13 ± 0.05	**4v**	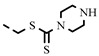	0.27 ± 0.01	0.27
**4c**	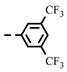	0.26 ± 0.02	0.25	**7c**	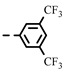	0.28	0.28
**4d**	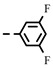	0.27 ± 0.01	0.27	**7d**	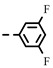	0.23	0.23
**4e**	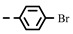	0.20 ± 0.01	0.19 ± 0.01	**7e**	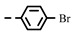	0.28	0.28
**4f**	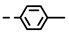	0.20 ± 0.02	0.14 ± 0.01	**7f**	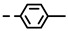	0.10 ± 0.02	0.10 ± 0.02
**4g**	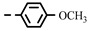	0.19 ± 0.01	0.19 ± 0.01	**7g**	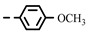	> 0.28	0.26
**4i**	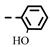	>0.28	>0.28	**7i**	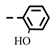	0.10 ± 0.05	0.10 ± 0.04
**4k**	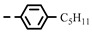	0.21 ± 0.07	0.13 ± 0.04	**7j**	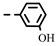	0.06	0.06
**4l**	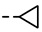	0.17 ± 0.16	0.16 ± 0.14	**7k**	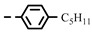	0.06	0.06
**4m**	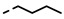	>0.28	>0.28	**7l**	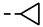	0.20 ± 0.01	0.20
**4n**	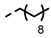	0.15 ± 0.06	0.10 ± 0.03	**7m**	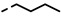	0.20	0.19
**4o**	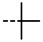	0.24 ± 0.028	0.24 ± 0.04	**7n**	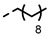	0.14	0.10
**4p**	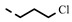	0.15 ± 0.06	0.15 ± 0.06	**7o**	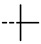	0.20	0.20
**4q**	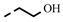	0.24 ± 0.02	0.23	**7p**	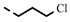	0.20	0.20
**4r**	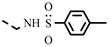	0.25	0.27 ± 0.02	**7q**	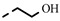	0.07	0.07
**4s**	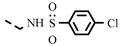	0.27 ± 0.02	0.25				

^a^ IC_50_ —concentration of compound required to scavenge 50% of DPPH^•^ free radicals.

**Table 2:** Calculated aqueous reaction free energy parameters (kcal/mol): acid dissociation constant of neutral specie (pK_a_) and its neutral radical (pK_a_^NR^), electron transfer free energies, and bond dissociation free energy for an anion (ETFE^A^ and BDFE^A^), and the ETFE of an intermediate radical anion (ETFE^RA^) (Figure 2) for C2-OH and C3-OH groups of selected compound pairs (saturated C4-C5(OH) analogues from series **4** and unsaturated C4=C5 analogues from series **7**).

Comp.	Active OH	pK_a_	ETFE^A^	pK_a_^NR^	BDFE^A^	ETFE^RA^
**L-ASA**	2-OH	10.3	72.6	−6.3	57.3	
	3-OH	3.6	79.6	−4.4	66.9	76.6
**L-ASA_C4=C5^a^**	2-OH	7.8	73.9	−4.4	61.3	
	3-OH	3.6	81.3	−5.6	66.9	77.9
						
**4b**	2-OH	10.4	72.7	−6.7	56.9	
	3-OH	3.7	79.3	−5.0	66.6	76.9
**7b**	2-OH	7.4	75.1	−5.0	61.6	
	3-OH	3.5	82.0	−5.8	67.0	79.0
						
**4e**	2-OH	10.4	71.0	−5.3	60.4	
	3-OH	3.5	82.0	−4.1	67.9	77.3
**7e**	2-OH	9.5	72.7	−4.2	60.4	
	3-OH	5.1	80.6	−5.4	66.5	79.7
						
**4g**	2-OH	10.2	79.8	−11.0	58.2	
	3-OH	3.4	79.2	−3.8	67.3	76.5
**7g**	2-OH	7.6	74.7	−4.6	61.8	
	3-OH	4.3	80.3	−5.4	66.3	78.3
						
**4i**	2-OH	10.4	72.5	−6.1	57.6	
	3-OH	3.3	79.8	−4.2	67.3	77.1
**7i**	2-OH	7.4	76.0	−5.1	62.3	
	3-OH	4.0	80.5	−5.0	66.9	78.1
						
**4q**	2-OH	10.7	72.8	−6.5	60.4	
	3-OH	2.4	82.3	−5.1	68.6	79.0
**7q**	2-OH	7.3	74.5	−4.2	62.0	
	3-OH	3.5	80.8	−5.1	67.1	78.0

*^a^*Not synthetized.

**Table 3:** The IC_50_ values (µM) for compounds **4b–4g**, **4i**, **4k–4t**, and **4v** for tested cell lines.

		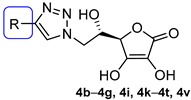									
Comp.	R	IC_50_^a^ (µM)									clogP^b^
		A549	CFPAC-1	HCT-116	HeLa	HepG2	MCF-7	SW620	WI-38	HFF-1	
**4b**	H	>100	>100	>100	>100	>100	>100	>100	>100	NA	−2.89
**4c**	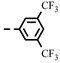	>100	>100	>100	>100	>100	>100	>100	>100	>100	0.56
**4d**	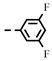	81.65	75.82	64.79	56.76	95.34	39.81	92.04	>100	0.12	−0.94
**4e**	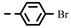	25.44	>100	>100	>100	>100	6.72	>100	73.93	>100	−0.42
**4f**	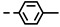	>100	>100	>100	>100	>100	>100	>100	>100	69.32	−0.80
**4g**	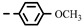	>100	>100	>100	>100	>100	>100	>100	>100	>100	−1.21
**4i**	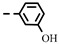	>100	>100	>100	>100	>100	>100	>100	>100	0.92	−1.49
**4k**	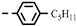	>100	>100	>100	>100	>100	26.91	>100	>100	0.21	0.98
**4l**	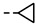	>100	>100	>100	>100	>100	>100	>100	>100	4.61	−1.73
**4m**	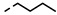	>100	>100	>100	>100	>100	>100	>100	>100	0.12	−1.17
**4n**	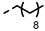	>100	>100	>100	>100	>100	>100	>100	>100	0.06	1.56
**4o**	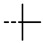	>100	>100	>100	93.04	>100	>100	>100	94.95	<0.01	−1.26
**4p**	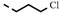	>100	>100	>100	48.01	>100	99.59	>100	52.65	6.05	−1.39
**4q**	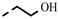	>100	>100	63.34	>100	>100	>100	>100	>100	>100	−3.00
**4r**	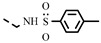	>100	>100	>100	>100	>100	>100	>100	>100	>100	−1.95
**4s**	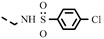	>100	>100	>100	>100	>100	>100	>100	>100	0.40	−1.69
**4t**	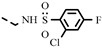	>100	>100	>100	>100	>100	>100	>100	>100	>100	−1.59
**4v**	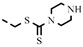	97.98	>100	>100	>100	>100	37.33	>100	>100	NA	−2.40
L-ASA		>100	>100	>100	>100	>100	>100	>100	NA	>100	−2.46
*C*arboxyamidotriazole		51.61	41.55	19.88	31.84	40.09	14.69	41.33	/	32.61	
5-FU		2.80	0.14	/	8.81	9.04	0.096	0.08	0.94	/	

^a^ Concentration of compounds required to inhibit human tumor cell lines and normal cell line growth by 50%; ^b^ values of n-octanol/water partition coefficients; clogP, were calculated by DataWarrior [44].

**Table 4:** Results of the Annexin V assay *^a^*.

Cell Line	Duration of Treatment	Comp.Concentration	Early Apoptosis (%)	Late Apoptosis (%)	Necrosis (%)
MCF-7	24 h	Control	18.2	4.4	0
		**4e** (6.72 µM)	30.6	6.4	0.8
		**4e** (13.44 µM)	5.3	38.1	15.9
	48 h	Control	11.4	10.6	0
		**4e** (6.72 µM)	46.6	4.2	0.8
		**4e** (13.44 µM)	9.1	2.3	0

^a^ Results are presented as percentages of early apoptotic cells, late apoptotic/primary necrotic cells, and necrotic cells after 24 h and 48 h treatment. Cells were treated with **4e** at 6.72 µM (1 × IC_50_) and at 13.44 µM (2 × IC_50_). Control—untreated MCF-7 cells.

## Supplementary Materials

Supplementary materials can be found at https://www.mdpi.com/1422-0067/20/19/4735/s1.
