# Supplementary material for: The Antioxidant and Antiproliferative Activities of 1,2,3-Triazolyl-L-Ascorbic Acid Derivatives
Source: Int J Mol Sci. 2019 Sep 24;20(19):4735. doi: 10.3390/ijms20194735 (PMC6801448; doi:10.3390/ijms20194735)

Supporting Information for:

Antioxidant and antiproliferative activities of 1,2,3-triazolyl-L-ascorbic acid derivatives

Anja Harej [1], Andrijana Meščić Macan [2,*], Višnja Stepanić [3], Krešimir Pavelić [4], Sandra Kraljević Pavelić [1,*], Silvana Raić-Malić [2]

[1] University of Rijeka, Department of Biotechnology, Centre for High-throughput Technologies, Radmile Matejčić 2, 51000 Rijeka, Croatia

[2] University of Zagreb, Department of Organic Chemistry, Faculty of Chemical Engineering and Technology, Marulićev trg 20, 10000 Zagreb, Croatia

[3] Ruđer Bošković Institute, Division of Electronics, Bijenička cesta 54, 10 000 Zagreb

[4] Juraj Dobrila University of Pula, Zagrebačka 30, 52100 Pula, Croatia

Contents:

Table S1.	**S2**
^{1}H and ^{13}C NMR spectra of compd.	**S3–S34**

Table S1. The growth-inhibition effects *in vitro* presented as IC50[a] (μM) for tested compounds **7c-7g** and **7i-7q** on selected tumour cell lines and normal fibroblasts.

Comp	R/X	IC50[a] (μM)								clogP[b]
		A549	CFPAC-1	HCT-116	HeLa	HepG2	MCF-7	SW620	WI-38	
**7c**	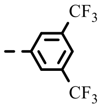	>100	>100	>100	>100	>100	>100	>100	>100	1.67
**7d**	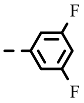	>100	>100	>100	>100	>100	>100	>100	>100	0.17
**7e**	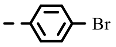	>100	>100	>100	>100	>100	>100	>100	>100	0.69
**7f**	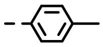	>100	>100	>100	>100	>100	>100	>100	>100	0.31
**7g**	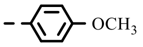	>100	>100	>100	>100	>100	>100	>100	>100	-0.10
**7i**	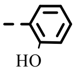	>100	>100	>100	>100	>100	>100	>100	>100	-0.38
**7j**	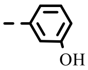	>100	>100	>100	>100	>100	>100	>100	>100	-0.38
**7k**	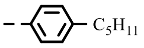	>100	>100	>100	>100	>100	>100	>100	>100	2.09
**7l**	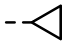	>100	>100	>100	>100	>100	>100	>100	>100	-0.62
**7m**	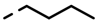	>100	>100	>100	>100	>100	>100	>100	>100	-0.06
**7n**	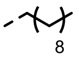	>100	>100	>100	>100	>100	>100	>100	>100	2.67
**7o**	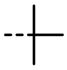	>100	>100	>100	>100	>100	>100	>100	>100	-0.15
**7p**	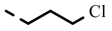	>100	>100	>100	>100	>100	>100	>100	>100	-0.28
**7q**	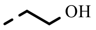	>100	>100	>100	>100	>100	>100	>100	>100	-1.89

[a]IC50: inhibitory concentration of compounds on human tumor cell lines and normal cell lines required inhibiting cells by 50%.
[b]Values of n-octanol/water partition coefficients clog P were calculated by DataWarrior [44].

Figure S1. a) ^{1}H NMR and b) ^{13}C NMR of compd. **4b.**

a)

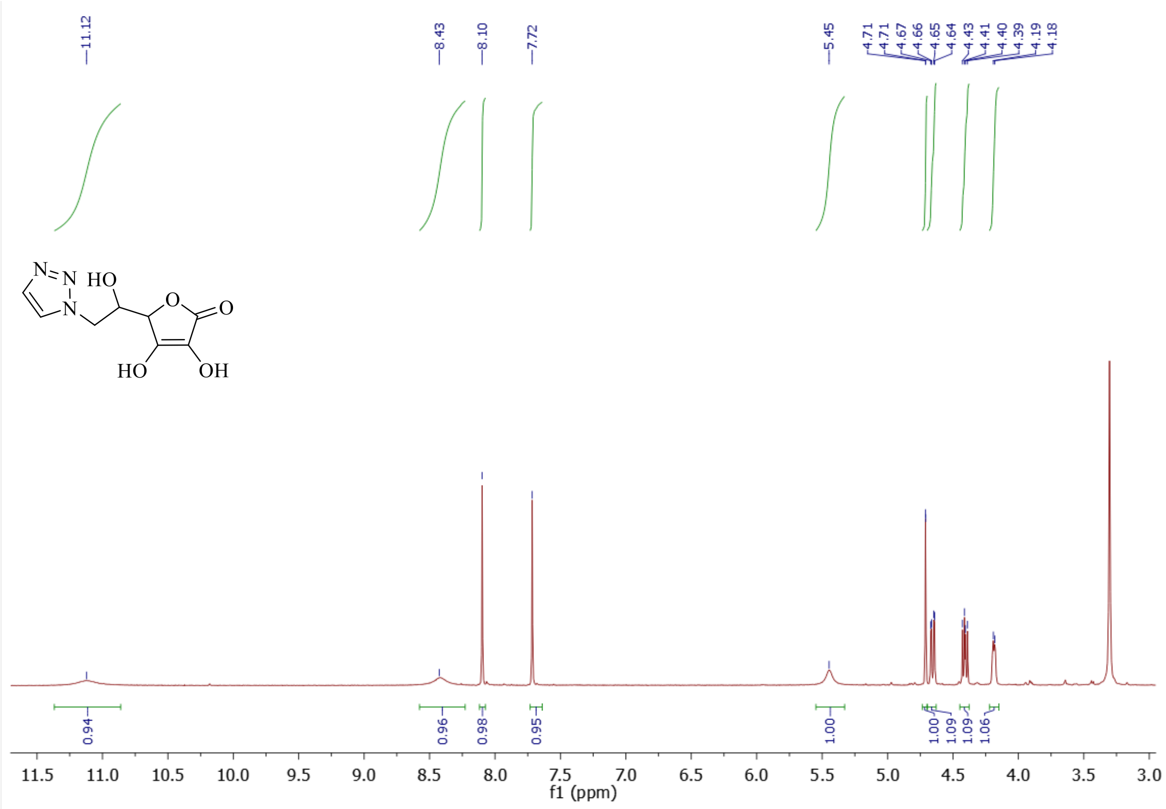

b)

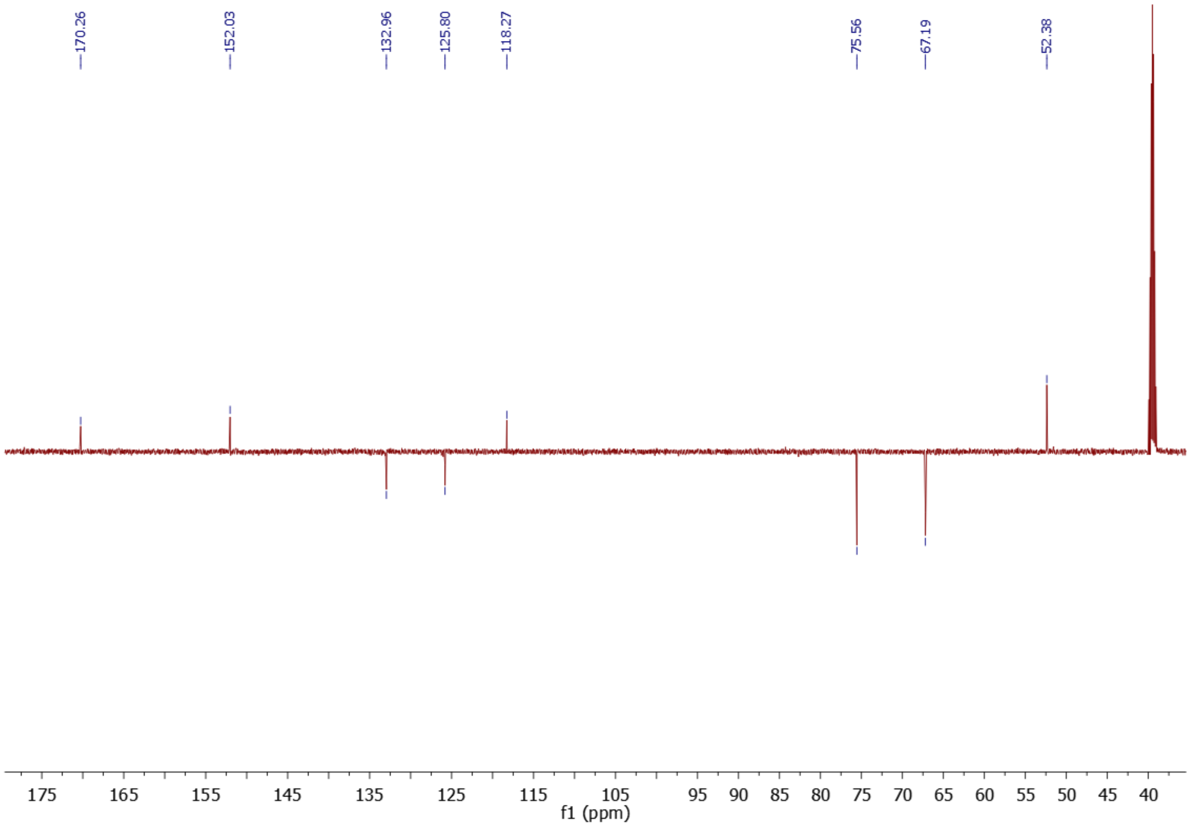

Figure S2. a) ^{1}H NMR and b) ^{13}C NMR of compd. **4c**.

a)

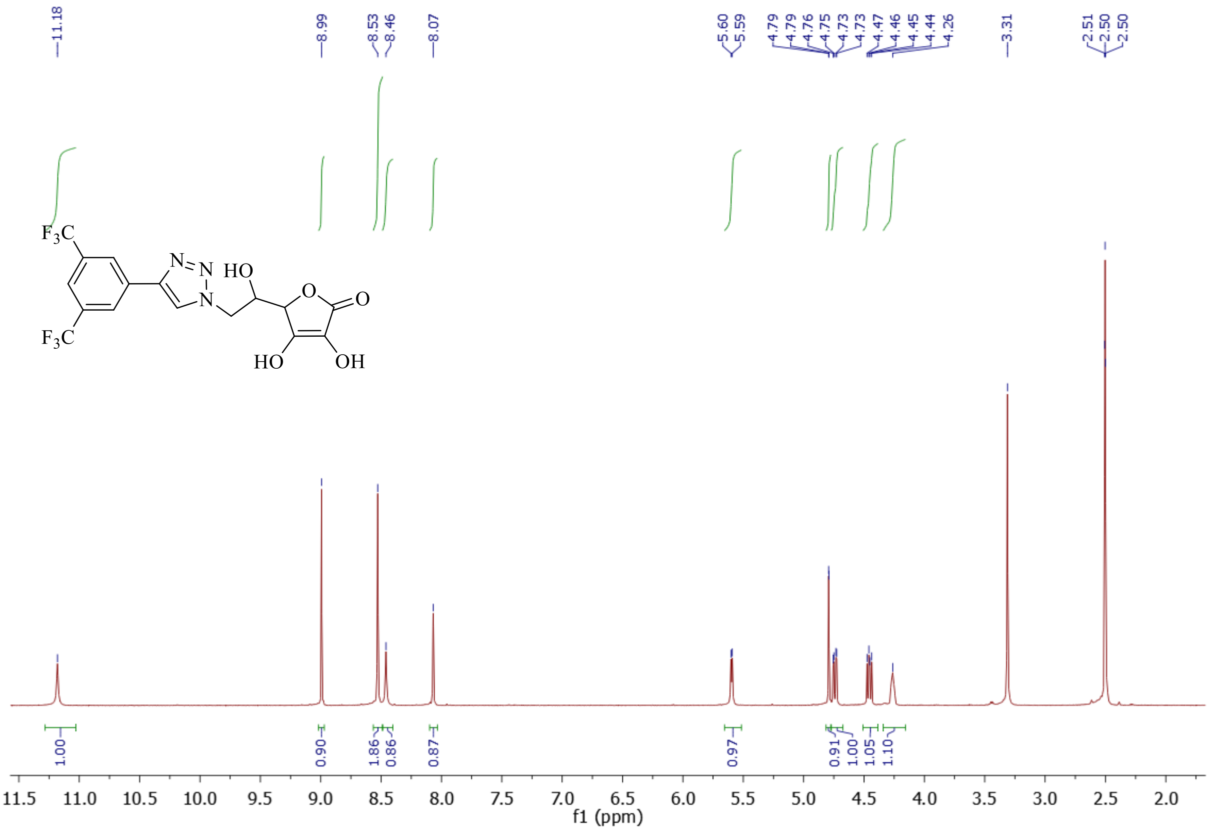

b)

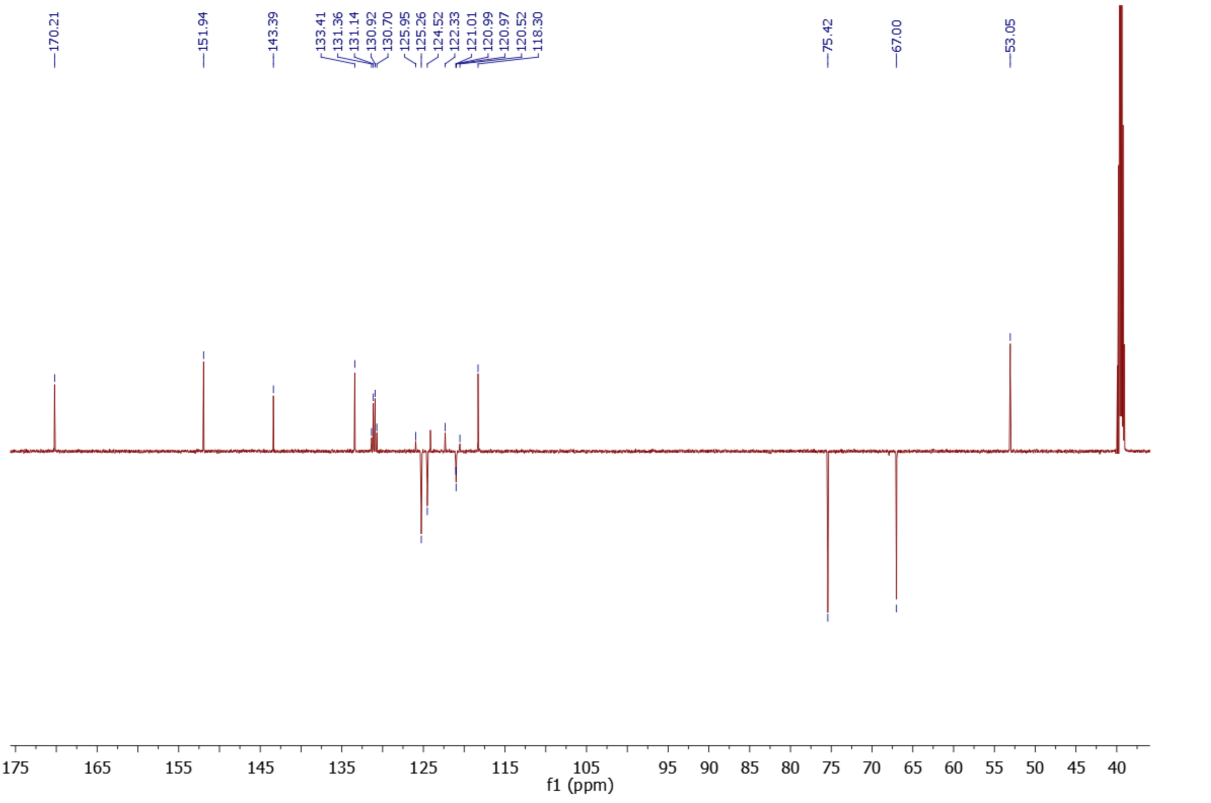

Figure S3. a) ^{1}H NMR and b) ^{13}C NMR of compd. **4d.**

a)

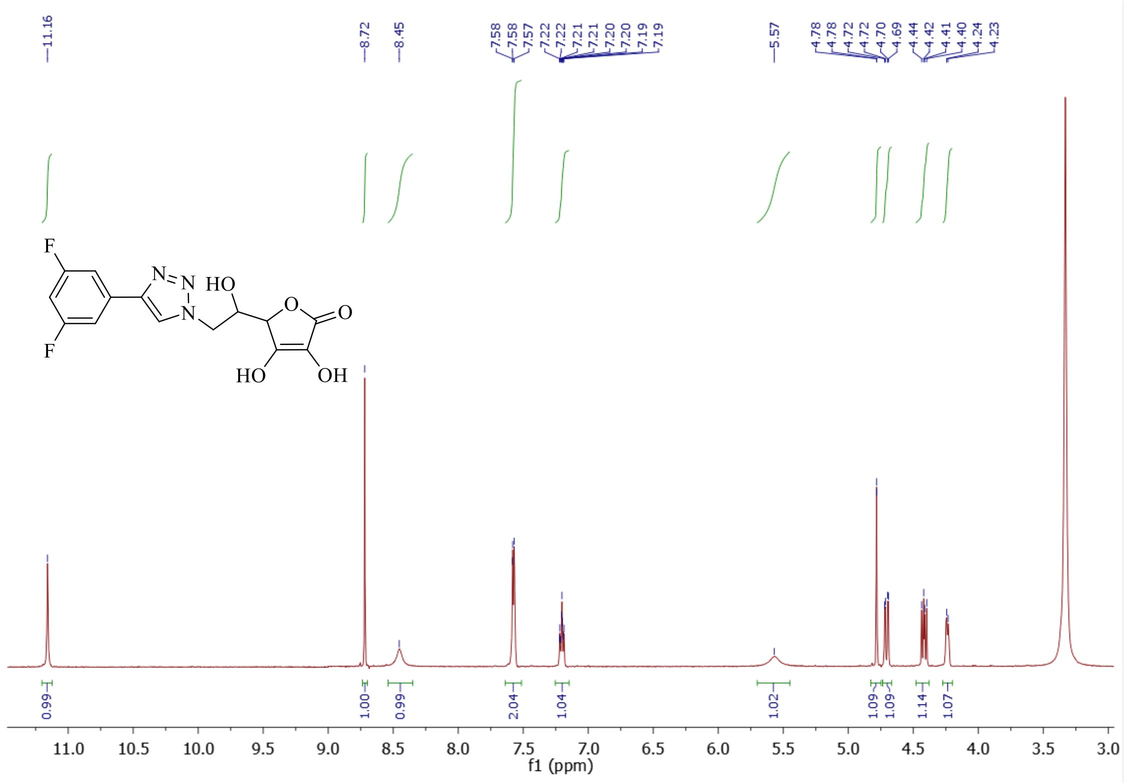

b)

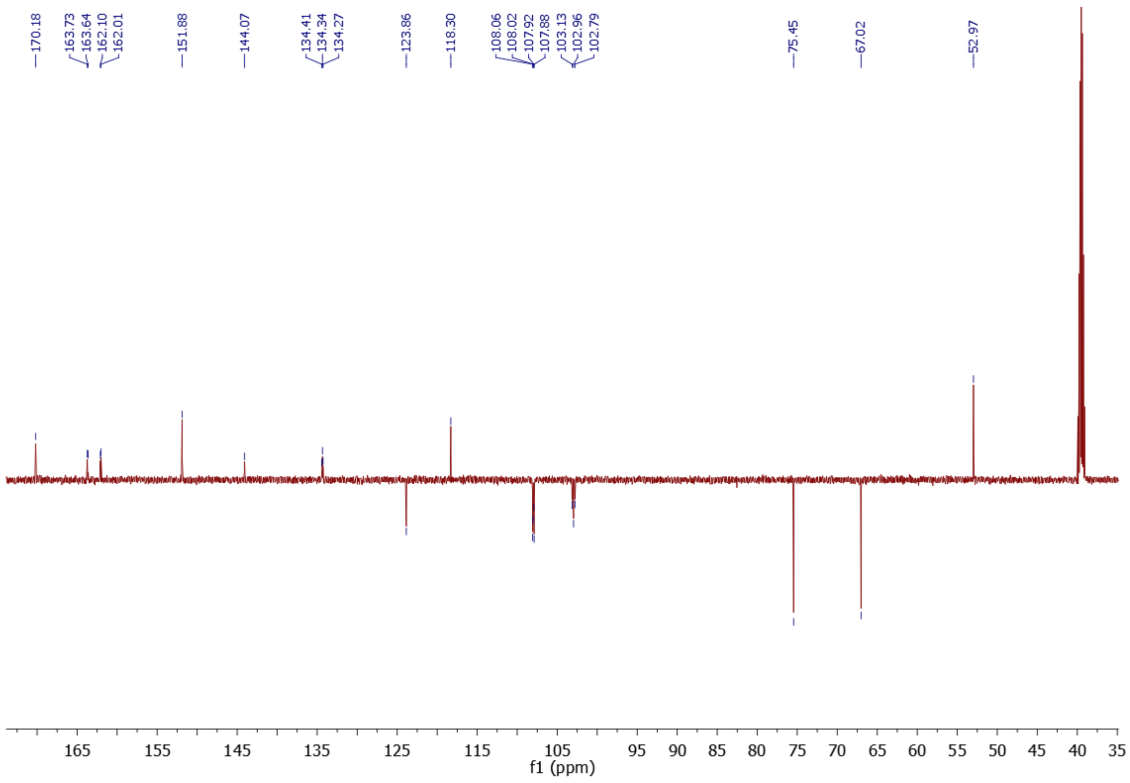

Figure S4. a) ^{1}H NMR and b) ^{13}C NMR of compd. **4e**.

a)

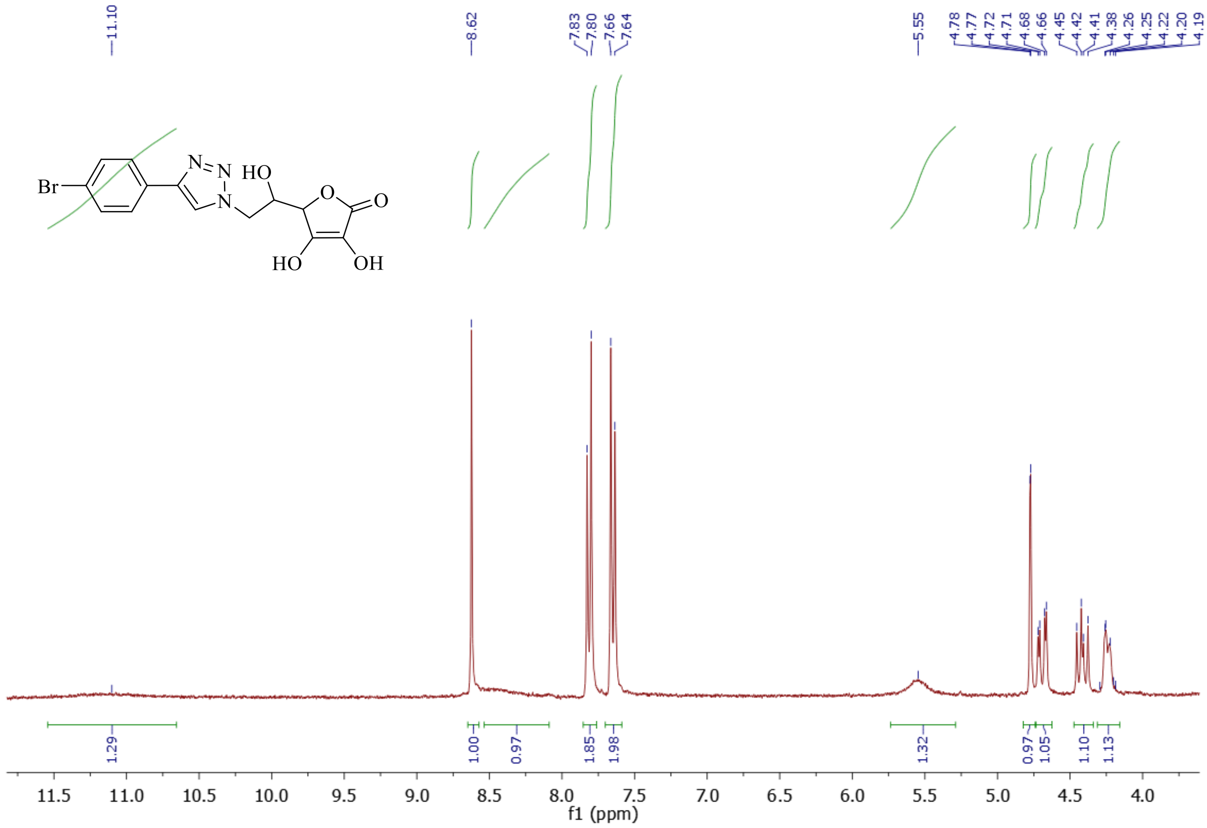
11.10
8.62
7.83
7.80
7.66
7.64
5.55
4.78
4.77
4.72
4.71
4.68
4.66
4.45
4.42
4.41
4.38
4.26
4.25
4.22
4.20
4.19
Br
N
N
N
HO
O
O
HO
OH
1.29
1.00
0.97
1.85
1.98
1.32
0.97
1.05
1.10
1.13
11.5
11.0
10.5
10.0
9.5
9.0
8.5
8.0
7.5
7.0
6.5
6.0
5.5
5.0
4.5
4.0
f1 (ppm)

b)

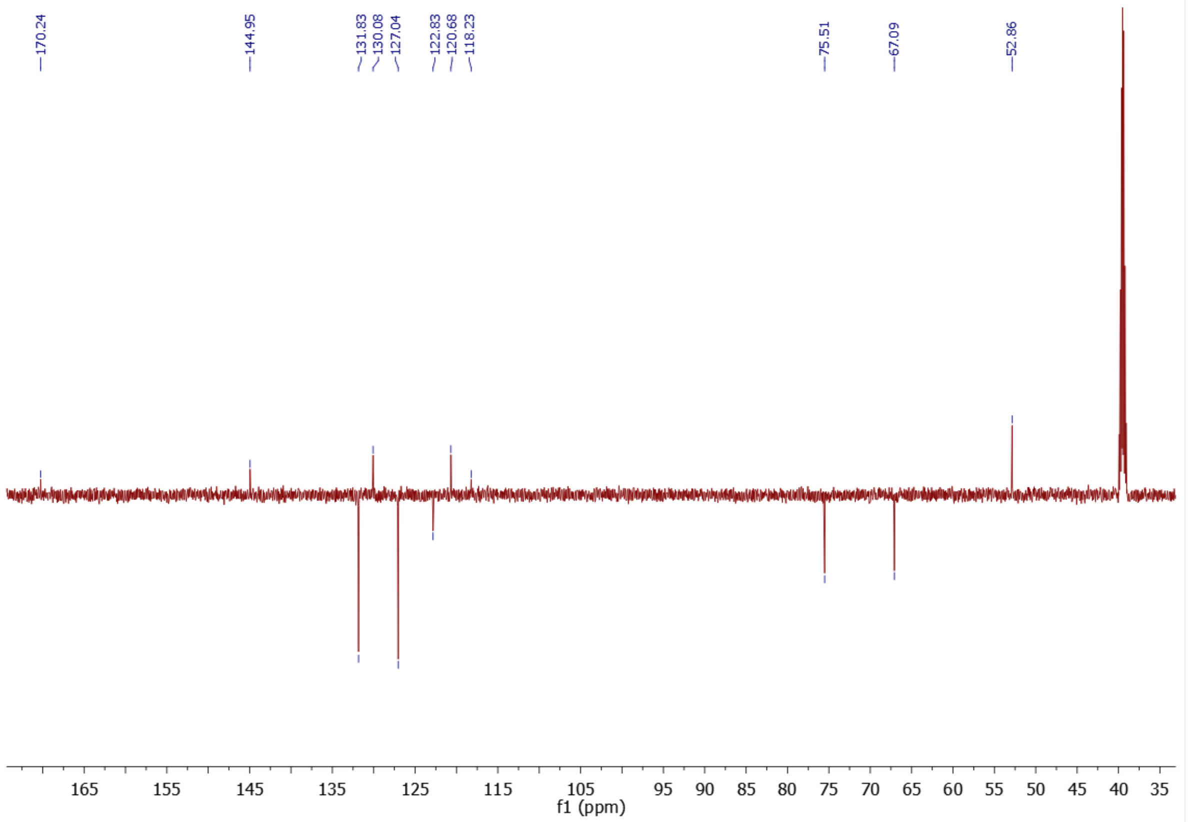
170.24
144.95
131.83
130.08
127.04
122.83
120.68
118.23
75.51
67.09
52.86
165
155
145
135
125
115
105
95
90
85
80
75
70
65
60
55
50
45
40
35
f1 (ppm)

Figure S5. a) ¹H NMR and b) ¹³C NMR of compd. **4f**.

a)

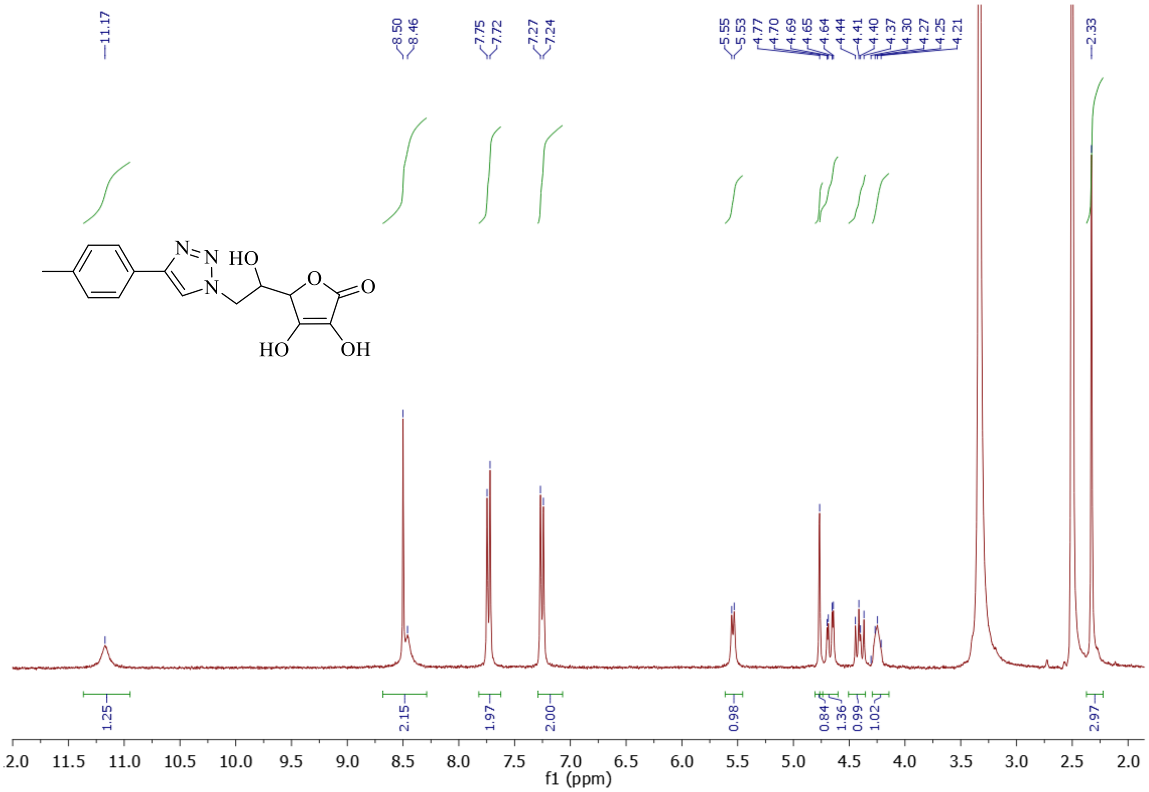

b)

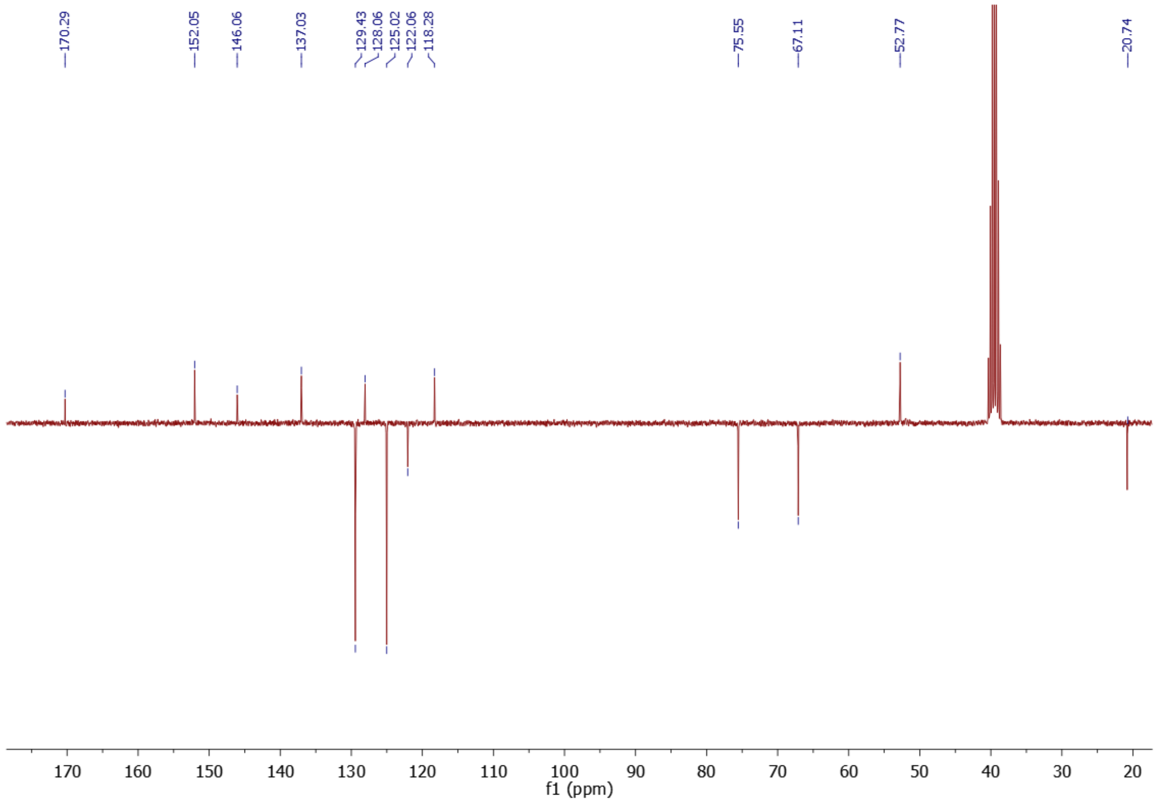

Figure S6. a) ^{1}H NMR and b) ^{13}C NMR of compd. **4g**.

a)

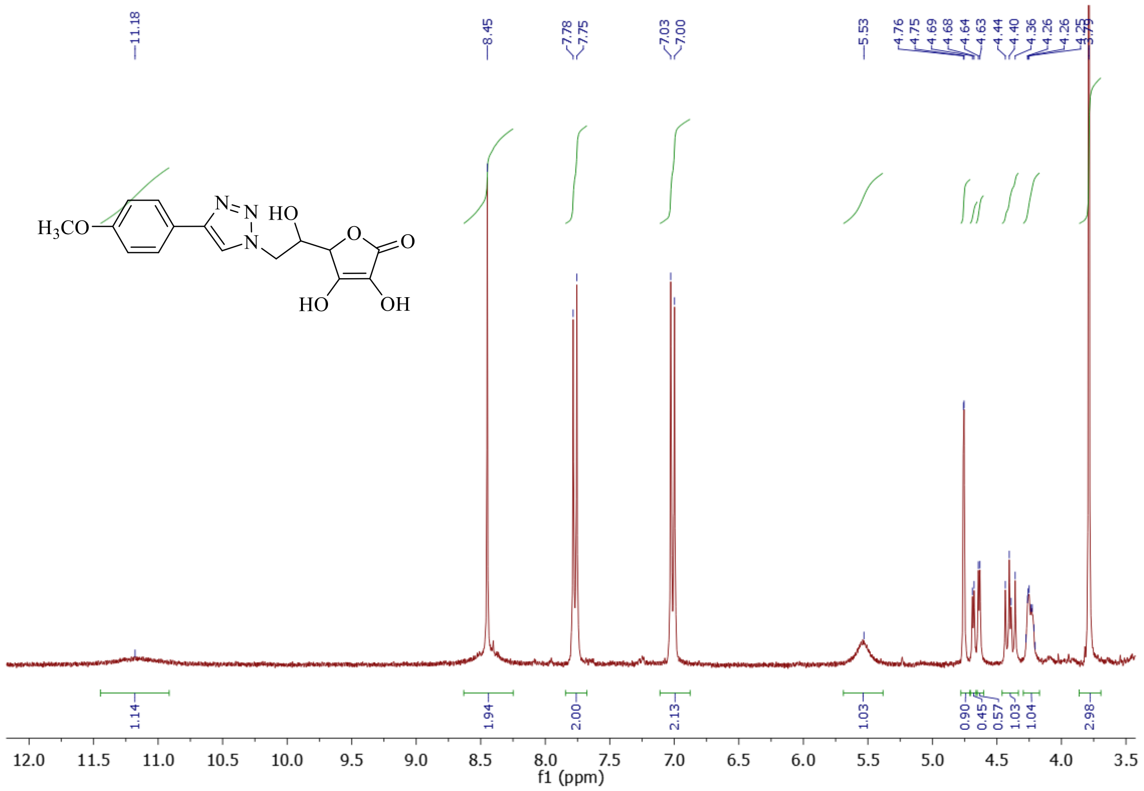

b)

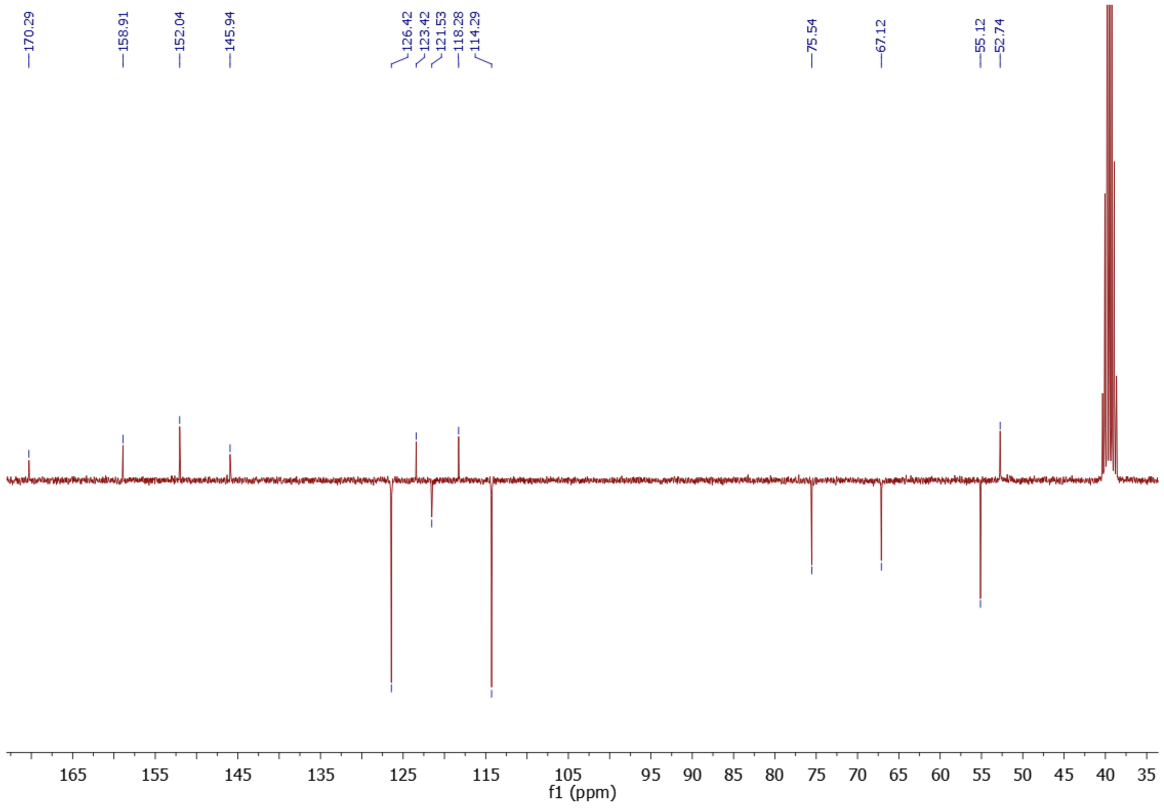

Figure S7. a) ^{1}H NMR and b) ^{13}C NMR of compd. **4i**.

a)

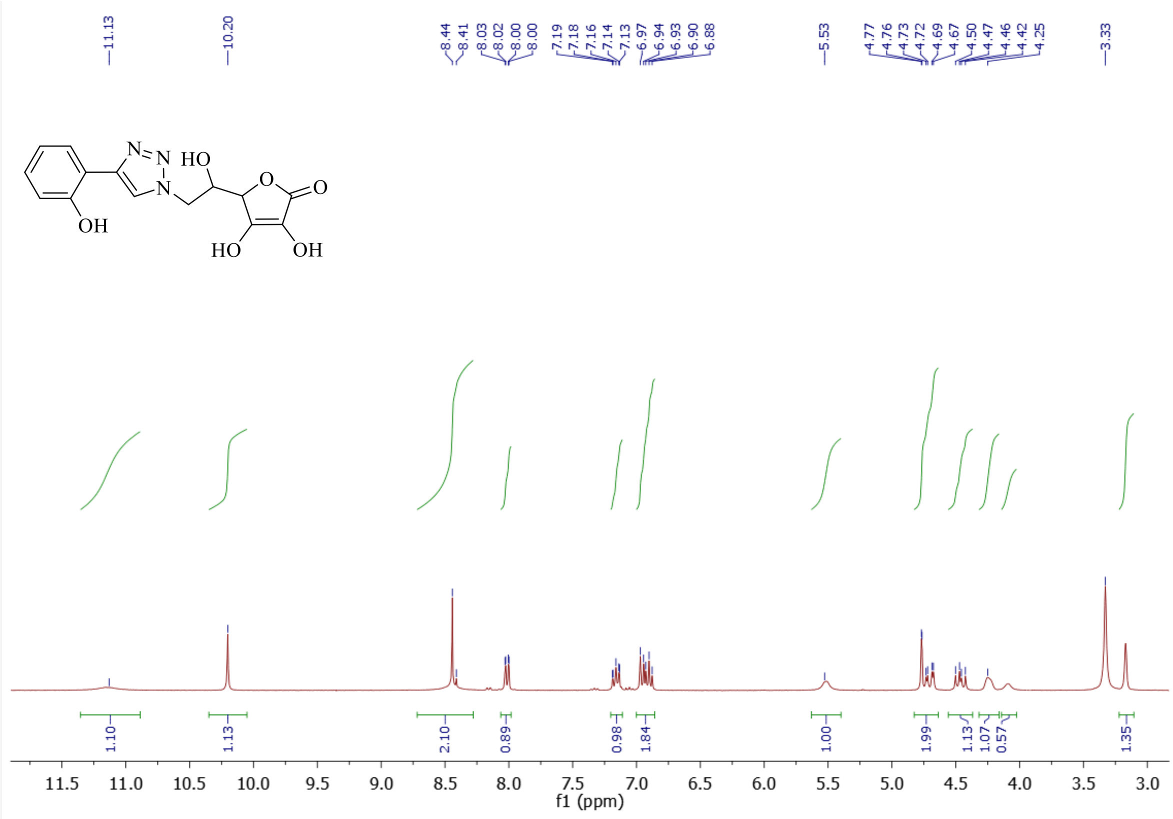

b)

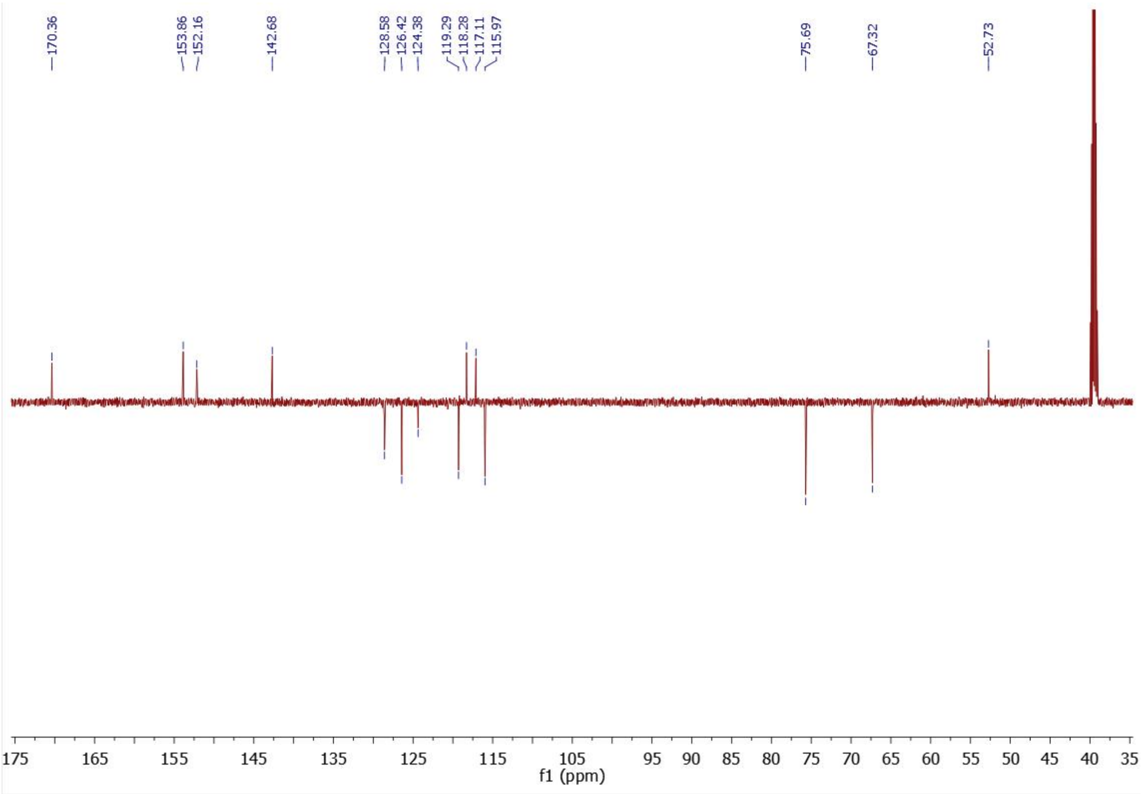

Figure S8. a) ^{1}H NMR and b) ^{13}C NMR of compd. **4k**.

a)

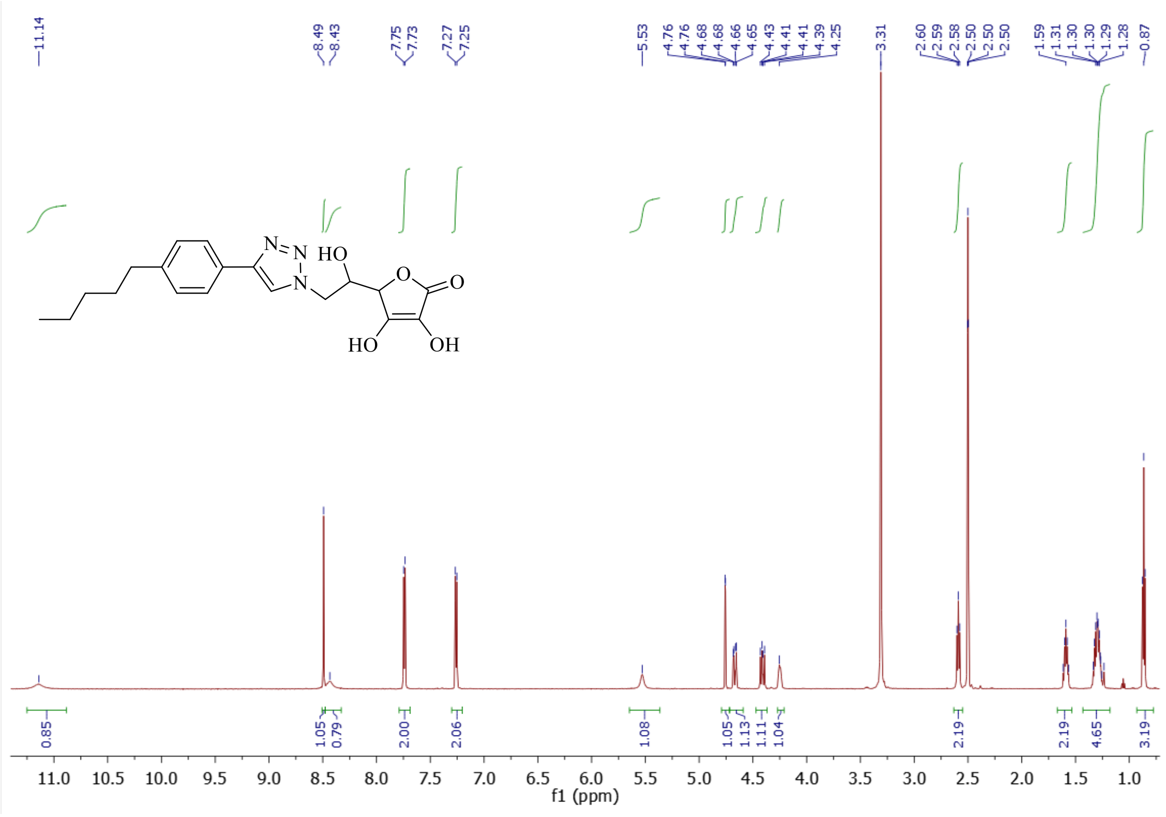

b)

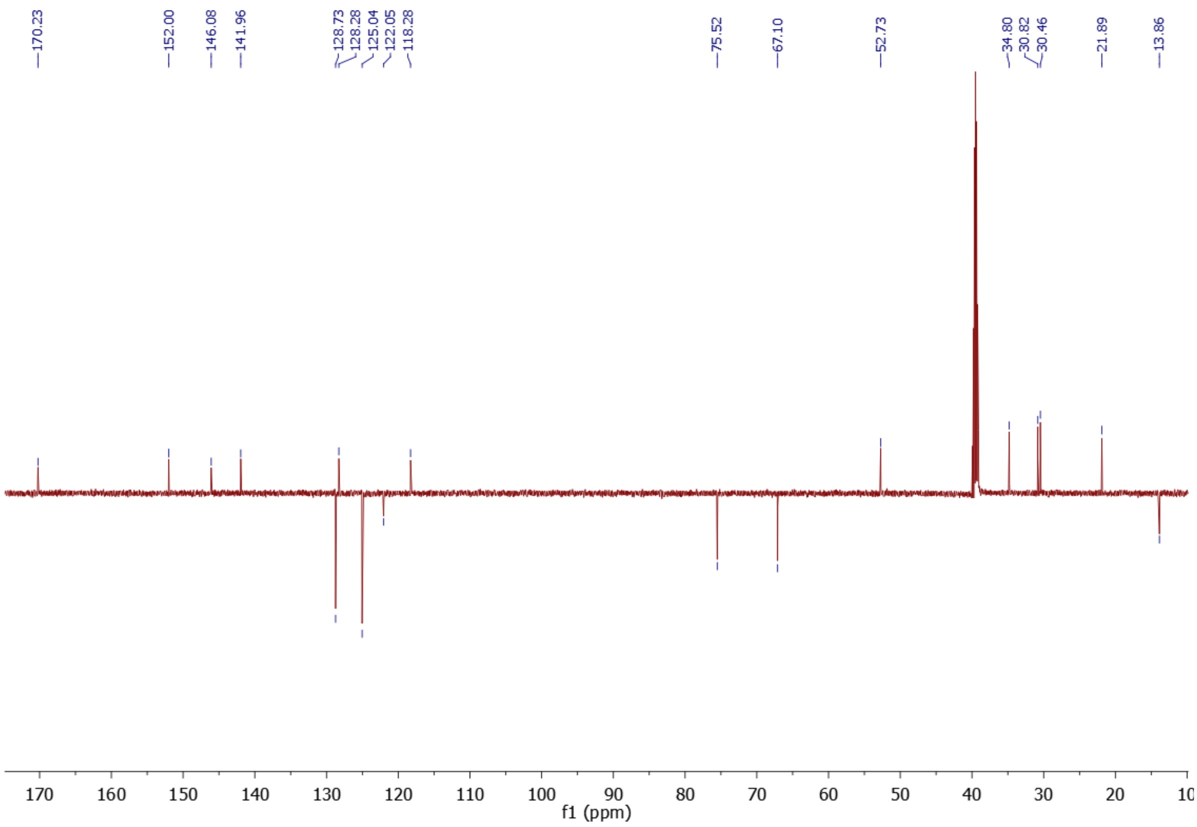

Figure S9. a) ^{1}H NMR and b) ^{13}C NMR of compd. **4l**.

a)

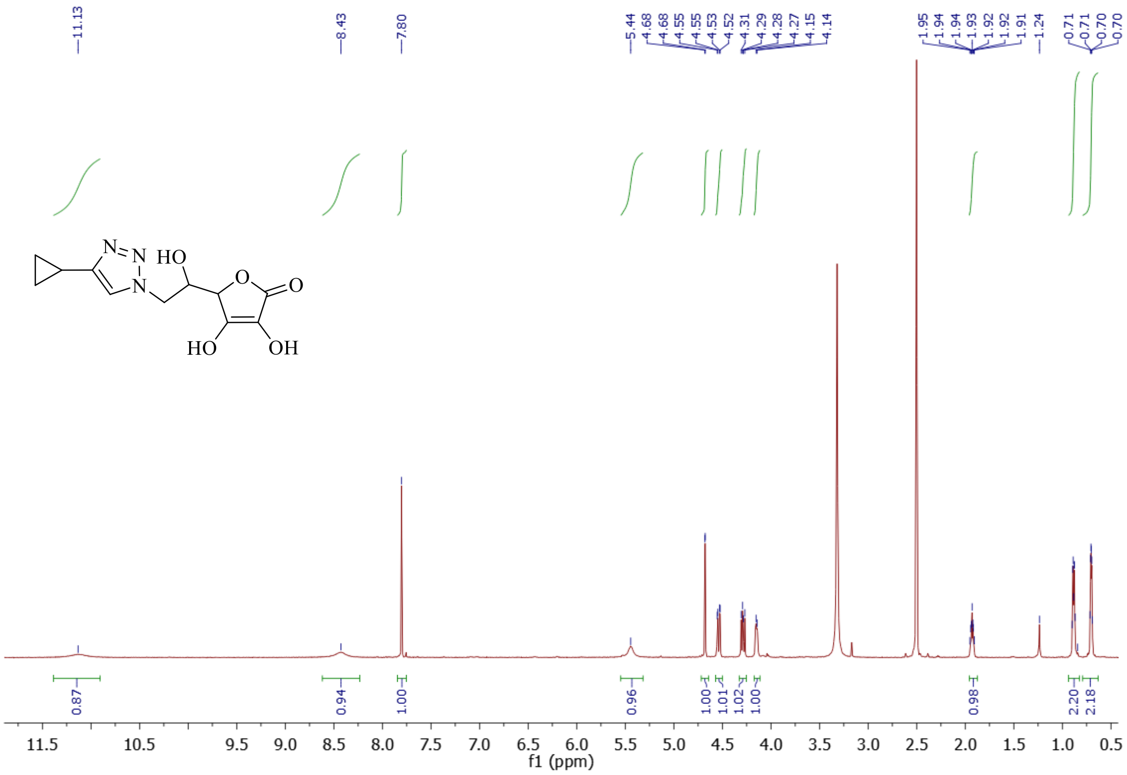

b)

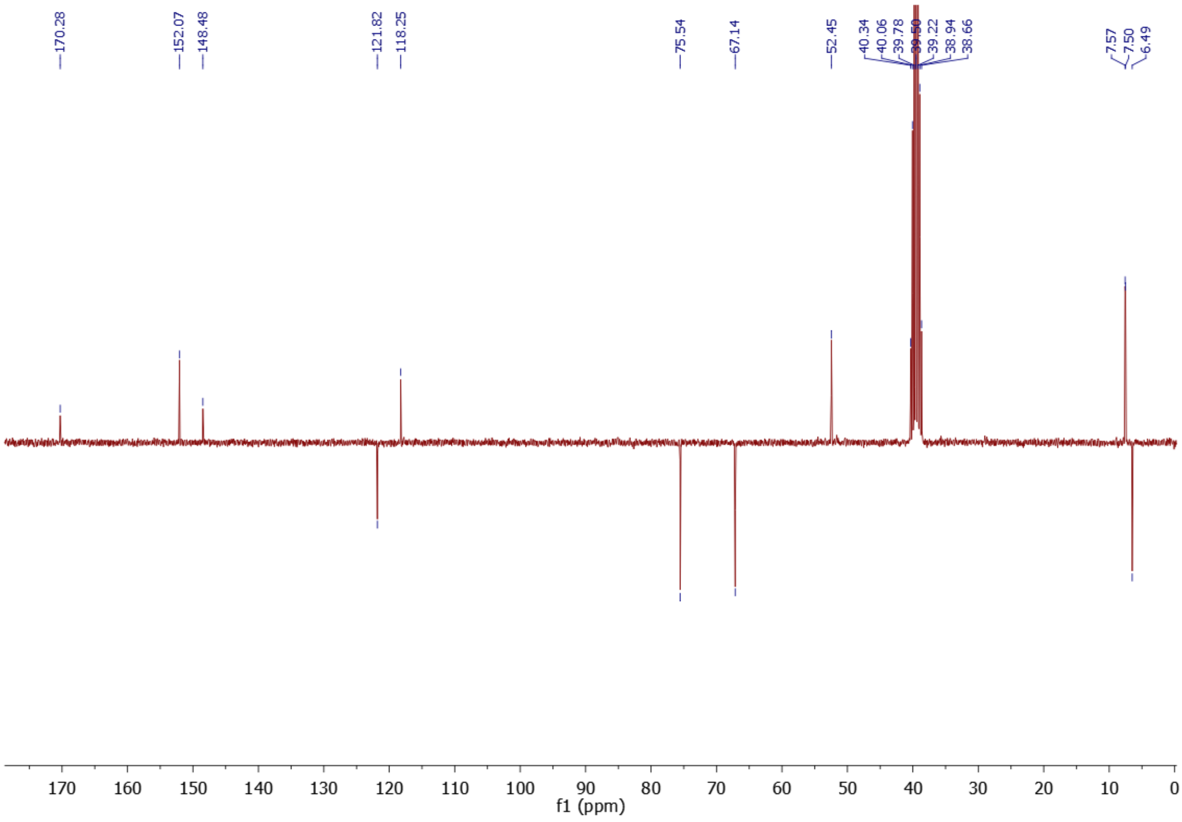

Figure S10. a) ^{1}H NMR and b) ^{13}C NMR of compd. **4m.**

a)

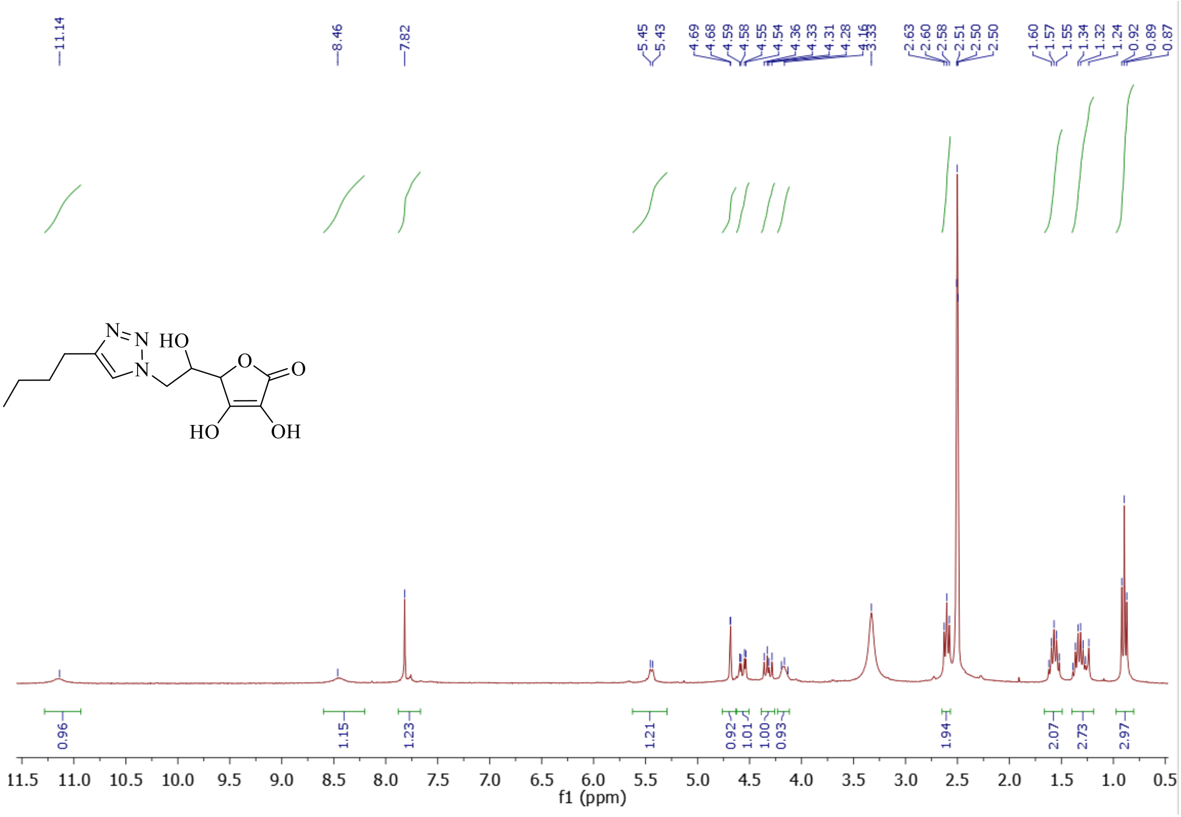

b)

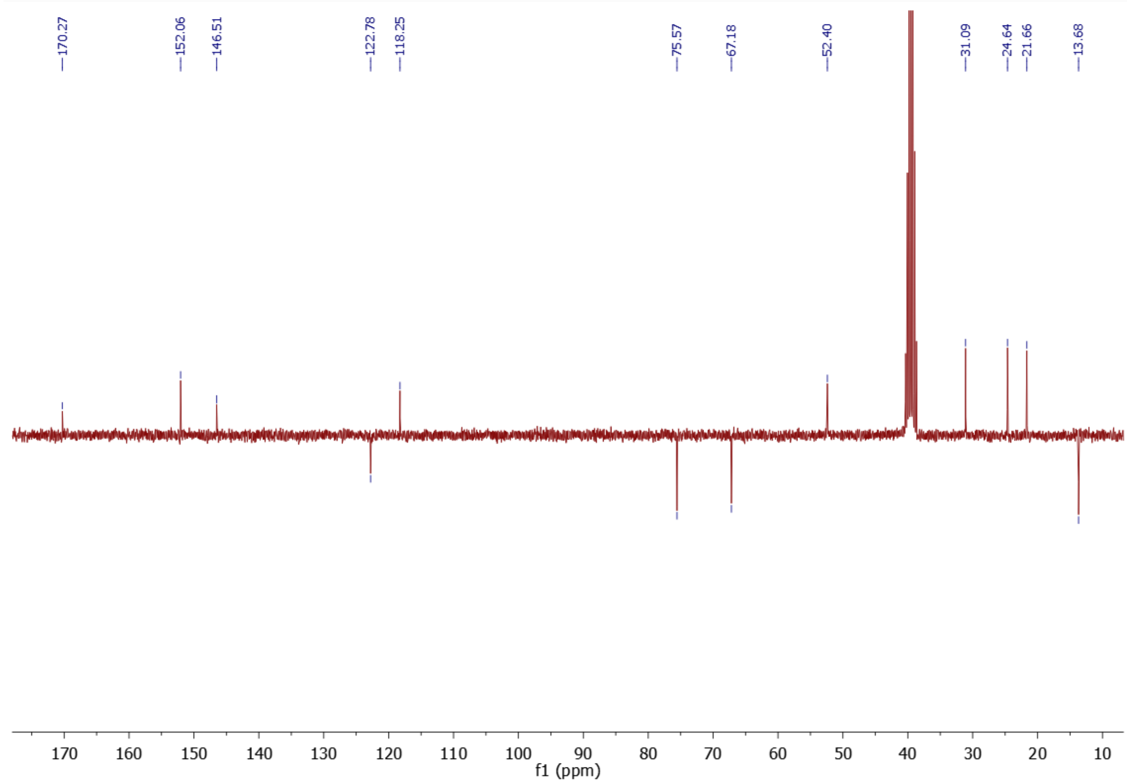

Figure S11. a) ^{1}H NMR and b) ^{13}C NMR of compd. **4n**

a)

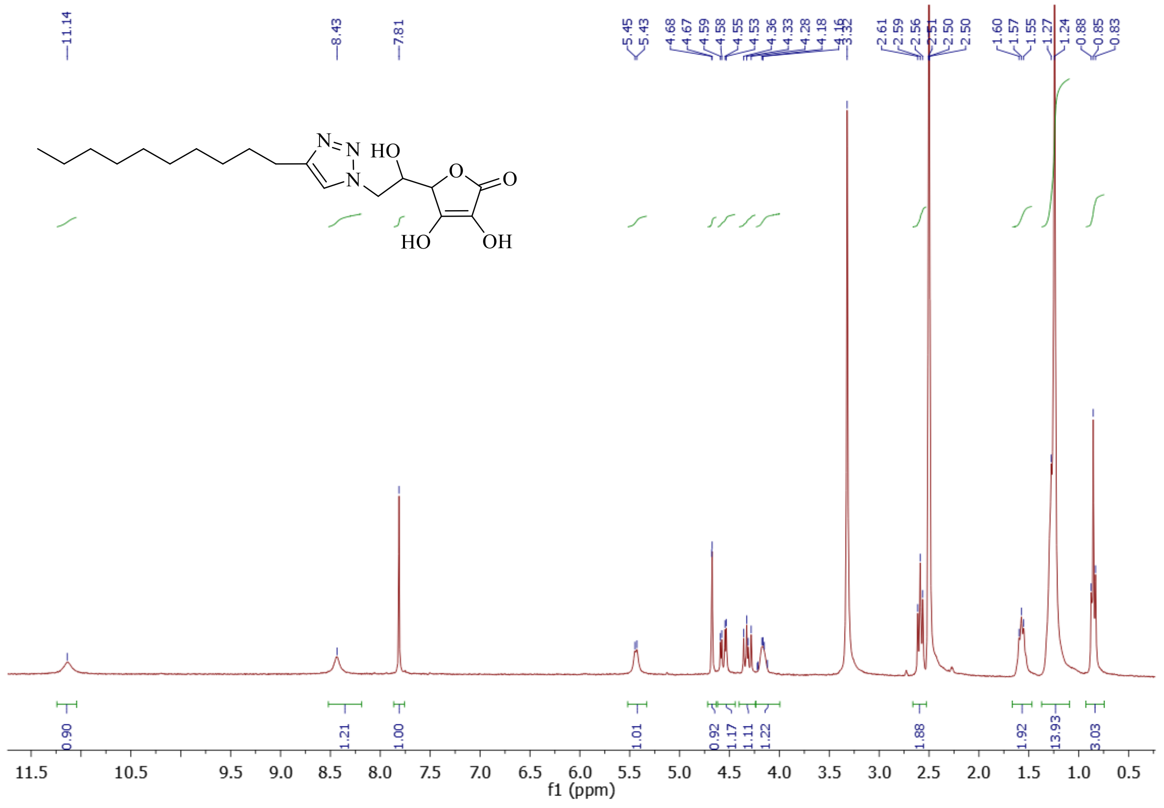

b)

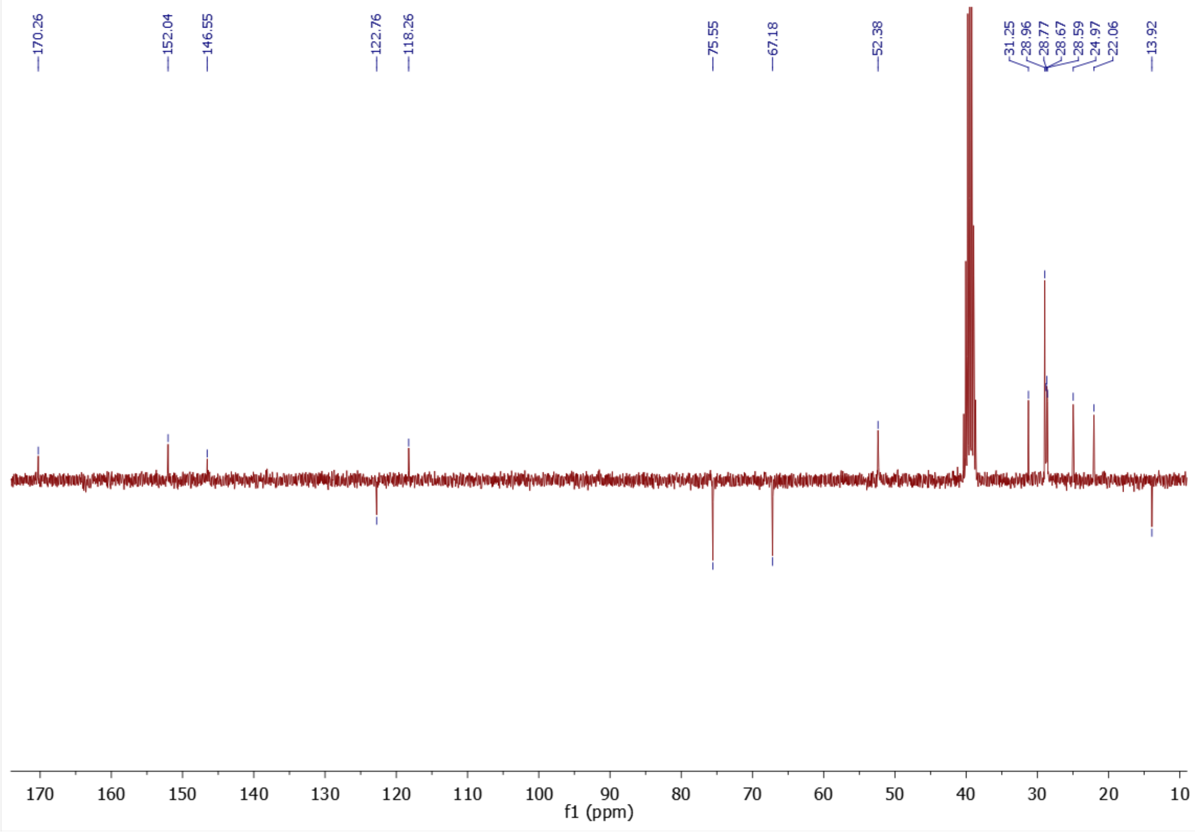

Figure S12. a) ^{1}H NMR and b) ^{13}C NMR of compd. **4o**.

a)

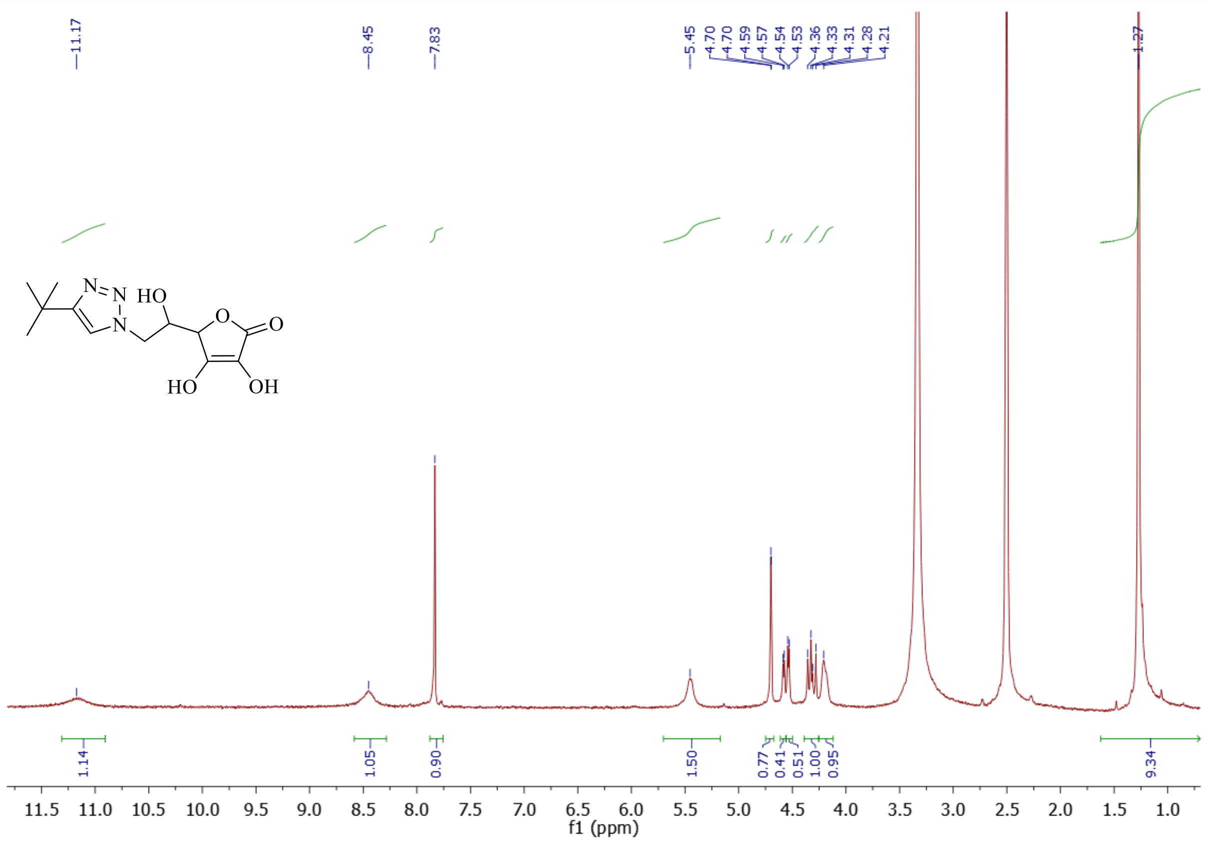

b)

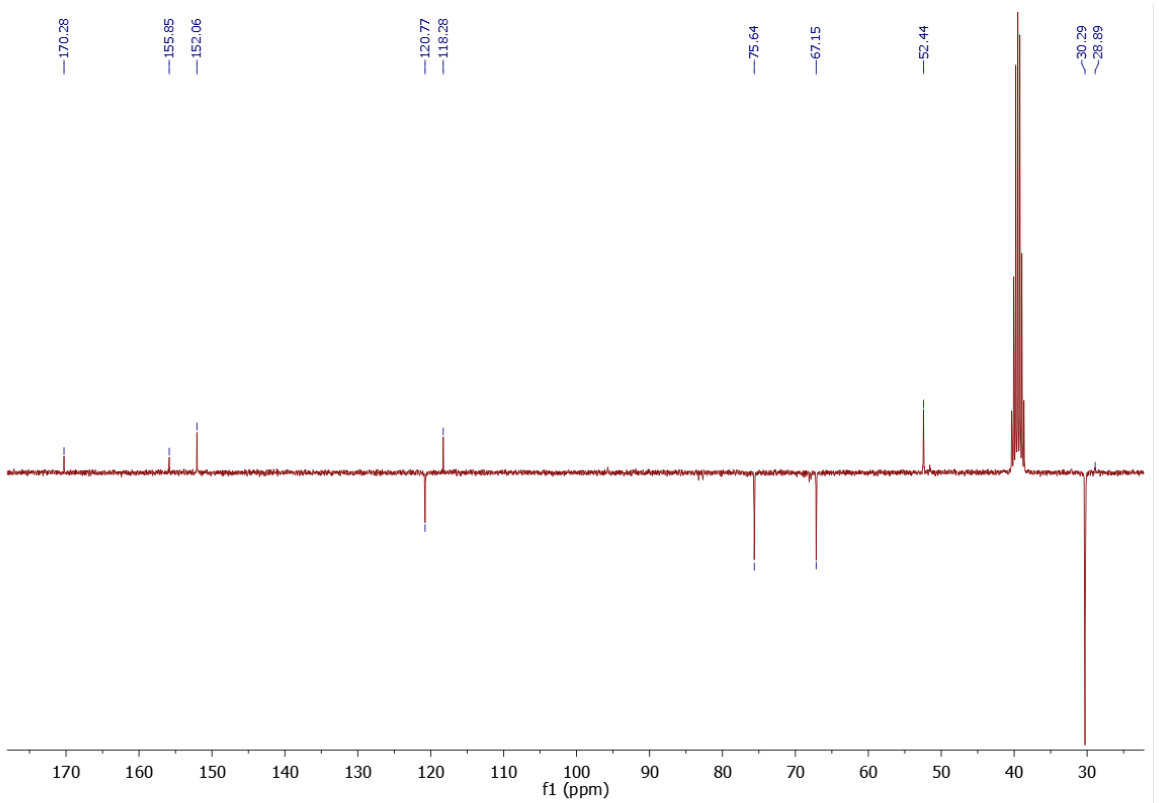

Figure S13. a) ^{1}H NMR and b) ^{13}C NMR of compd. **4p**.

a)

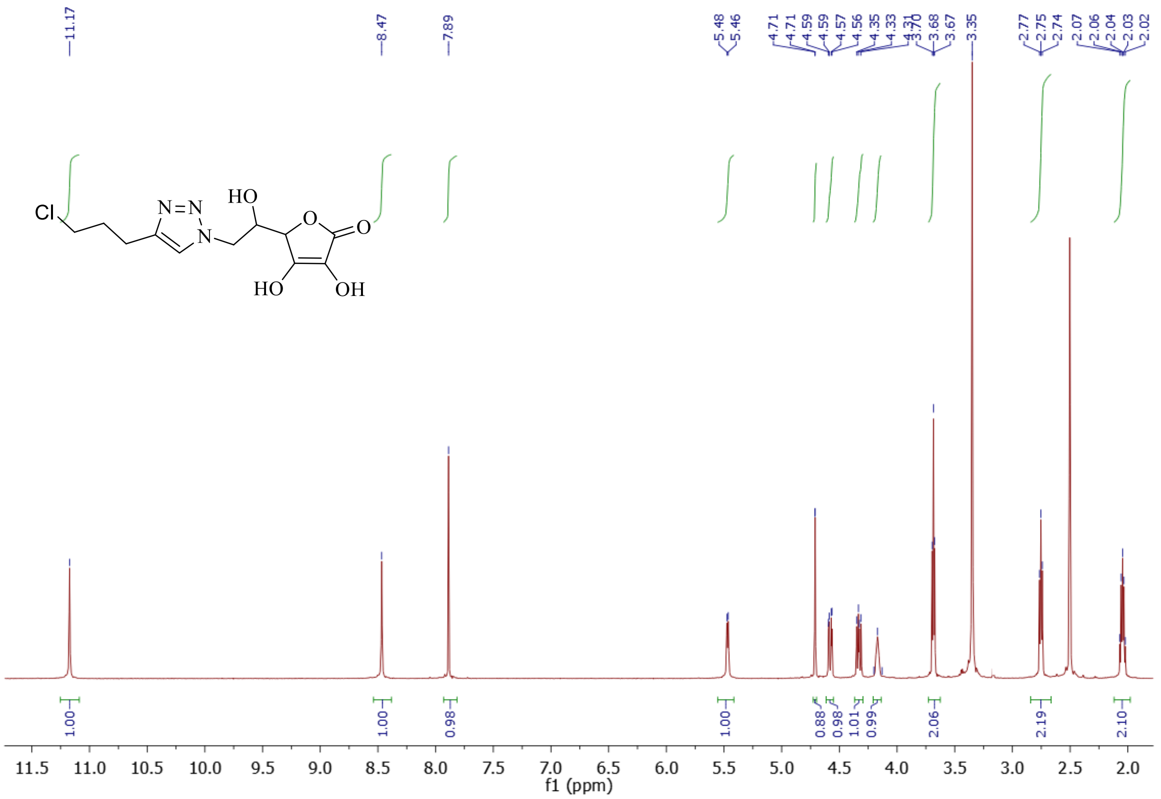

b)

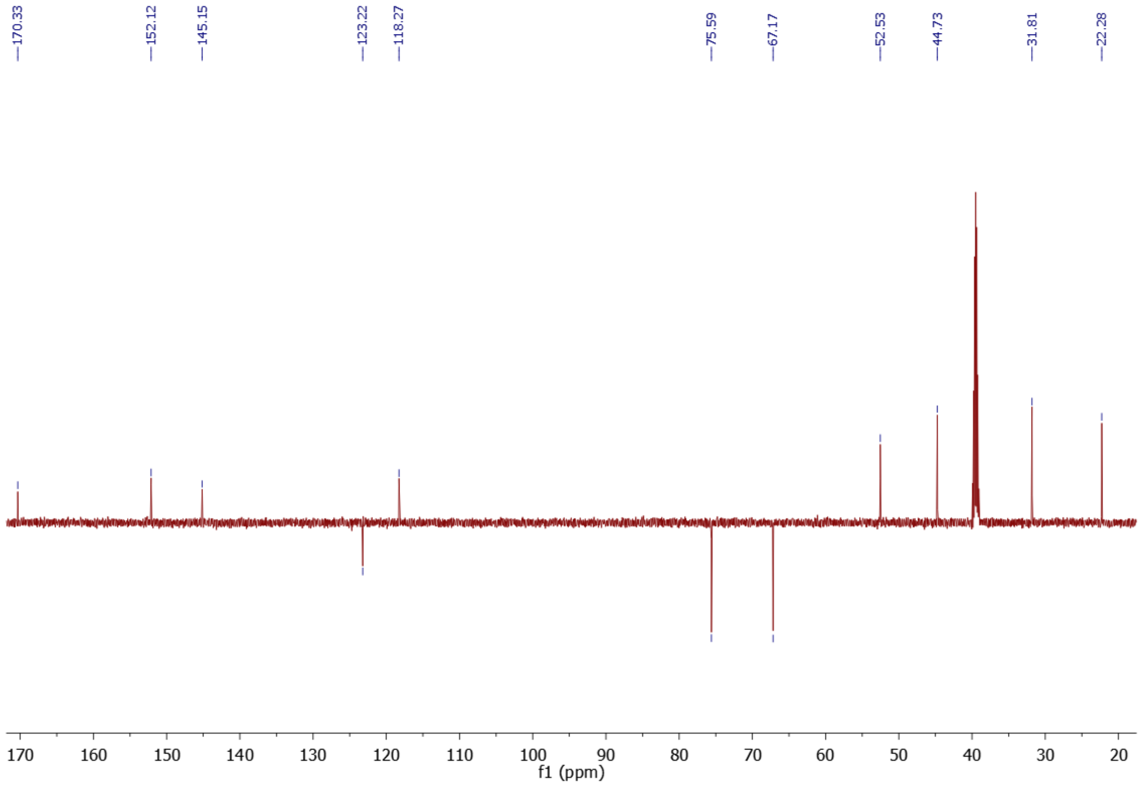

Figure S14. a) ^{1}H NMR and b) ^{13}C NMR of compd. **4q**.

a)

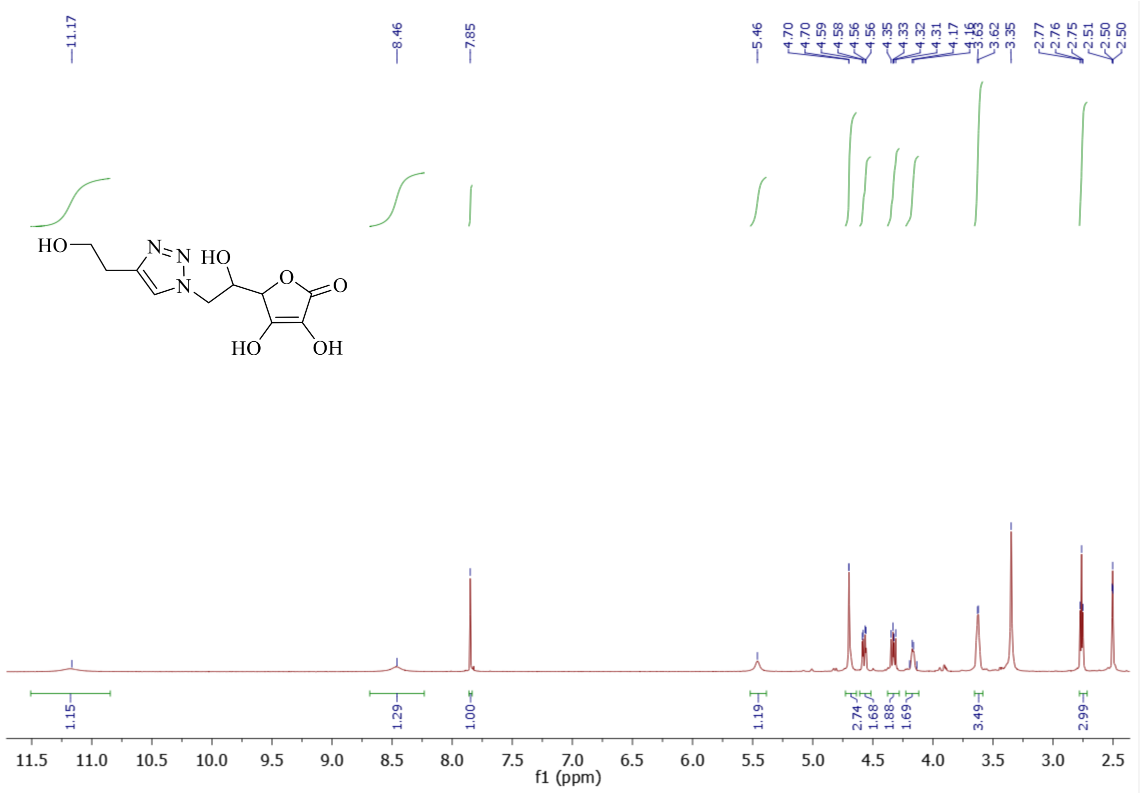

b)

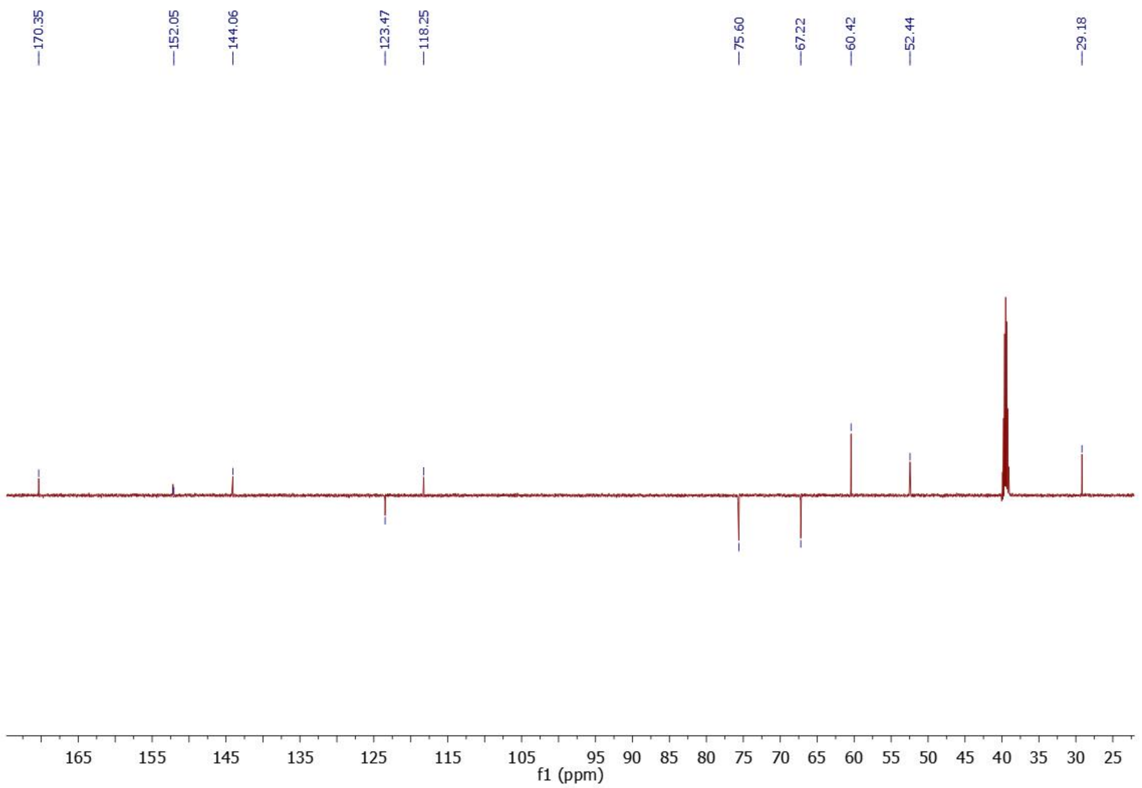

Figure S15. a) ^{1}H NMR and b) ^{13}C NMR of compd. **4r**.

a)

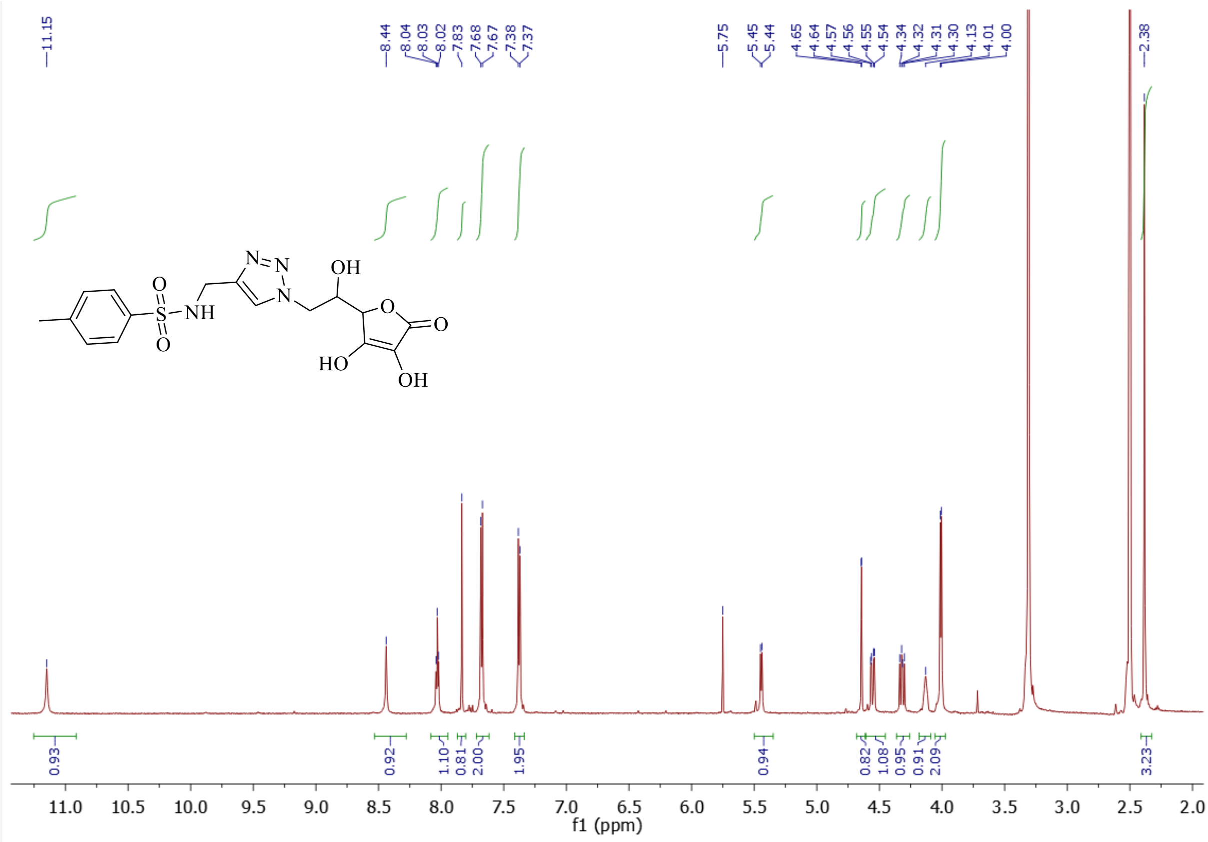

b)

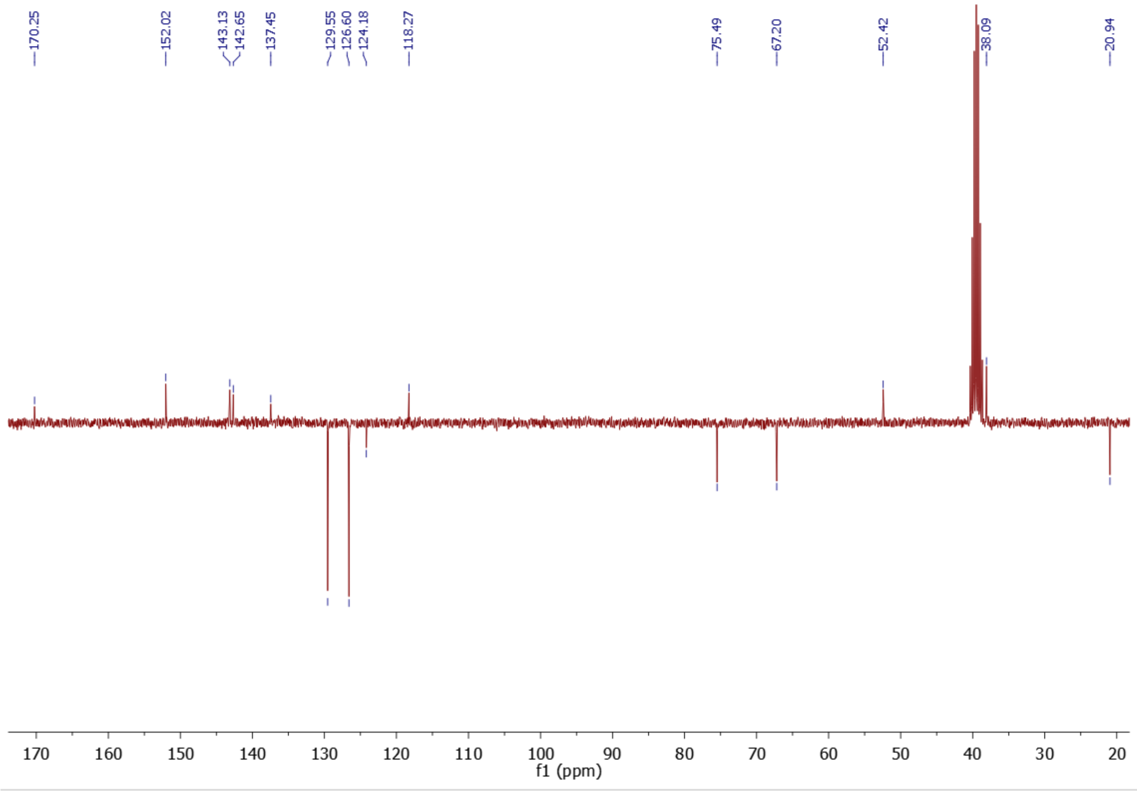

Figure S16. a) ^{1}H NMR and b) ^{13}C NMR of compd. **4s**.

a)

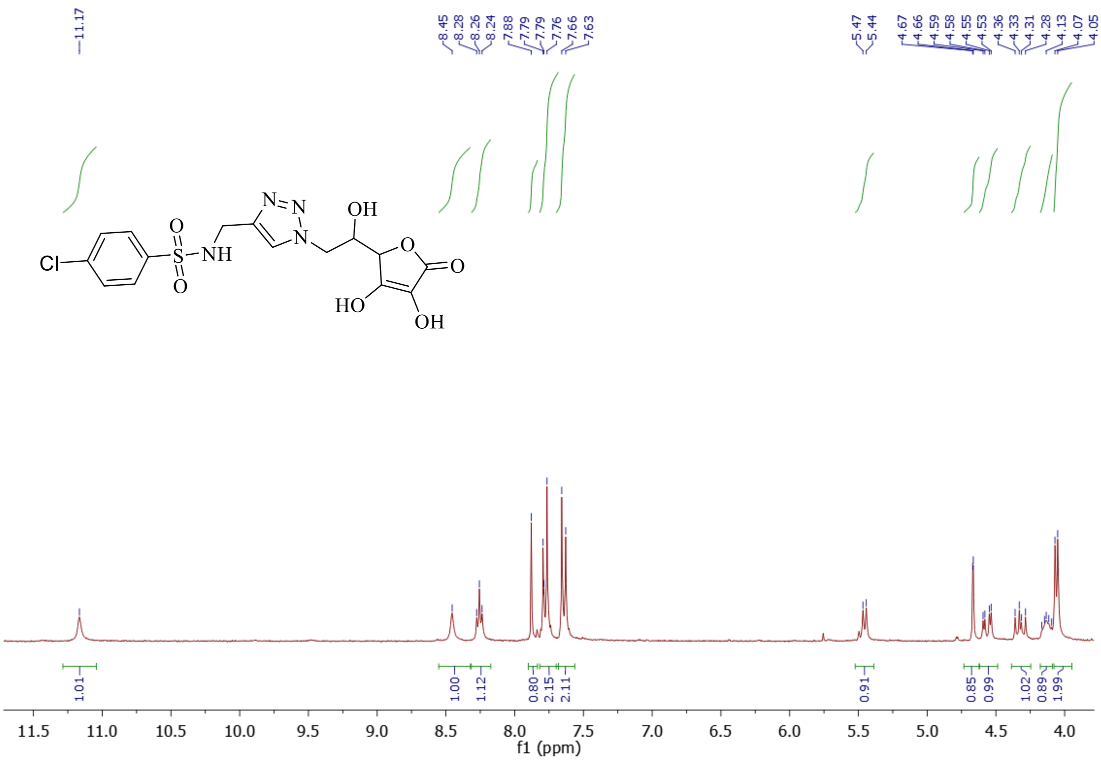

b)

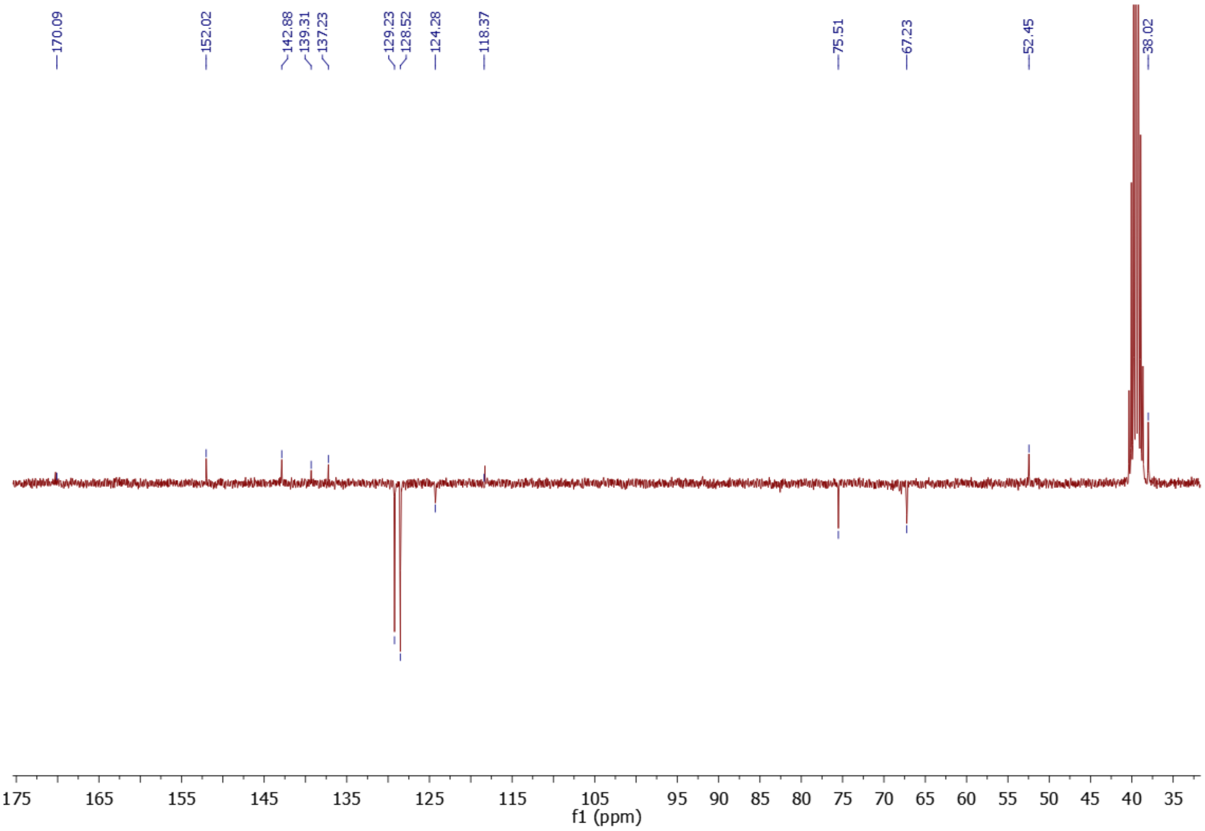

Figure S17. a) ^{1}H NMR and b) ^{13}C NMR of compd. **4t**.

a)

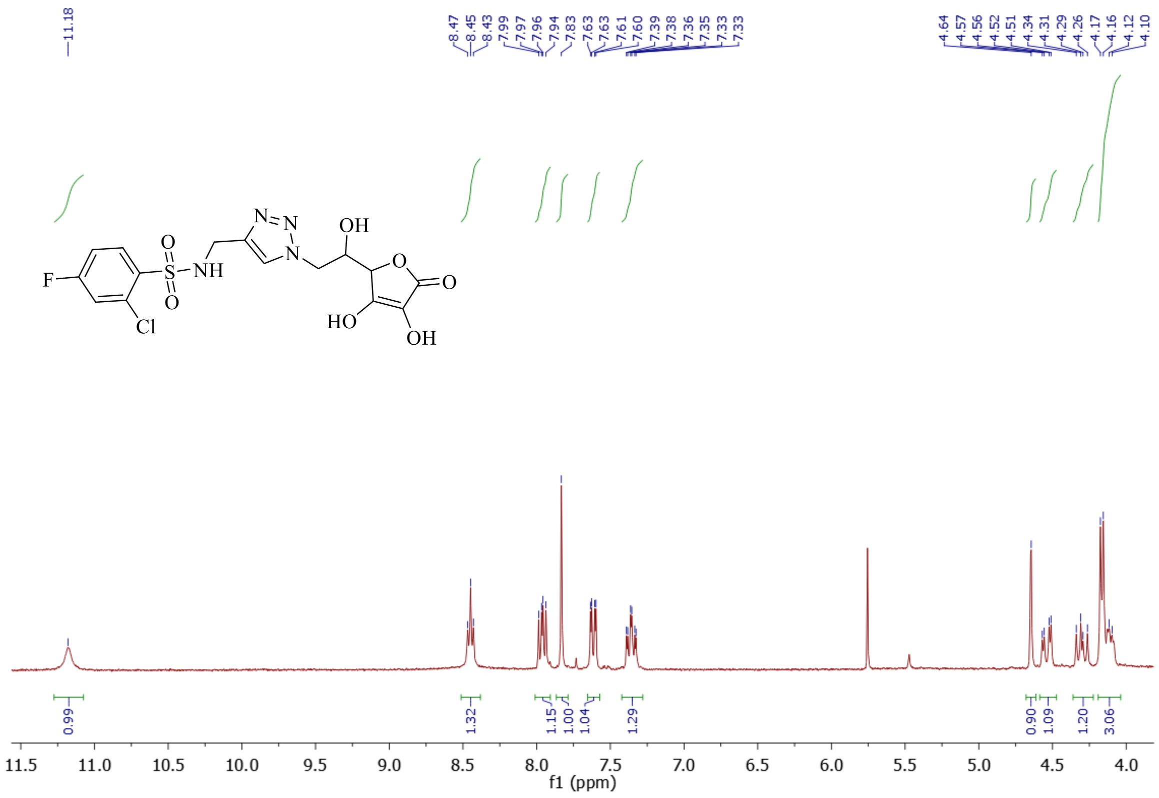

b)

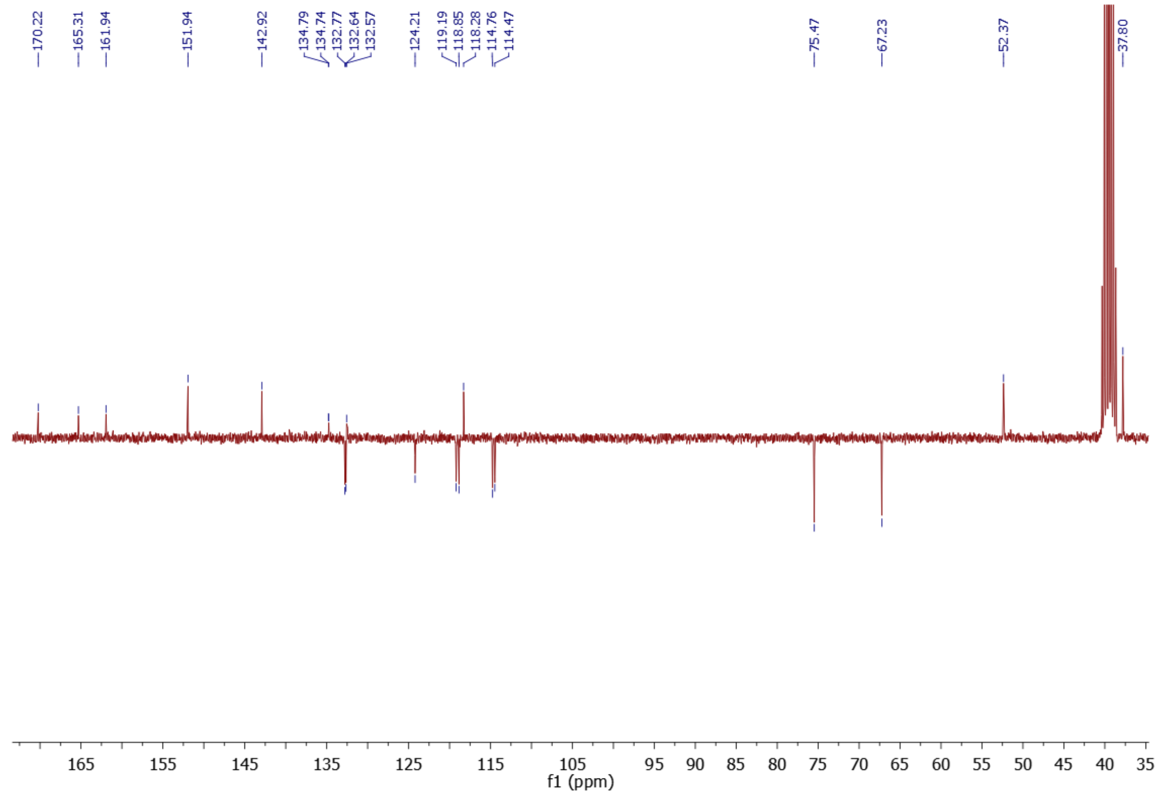

Figure S18. a) ^{1}H NMR and b) ^{13}C NMR of compd. **4v**.

a)

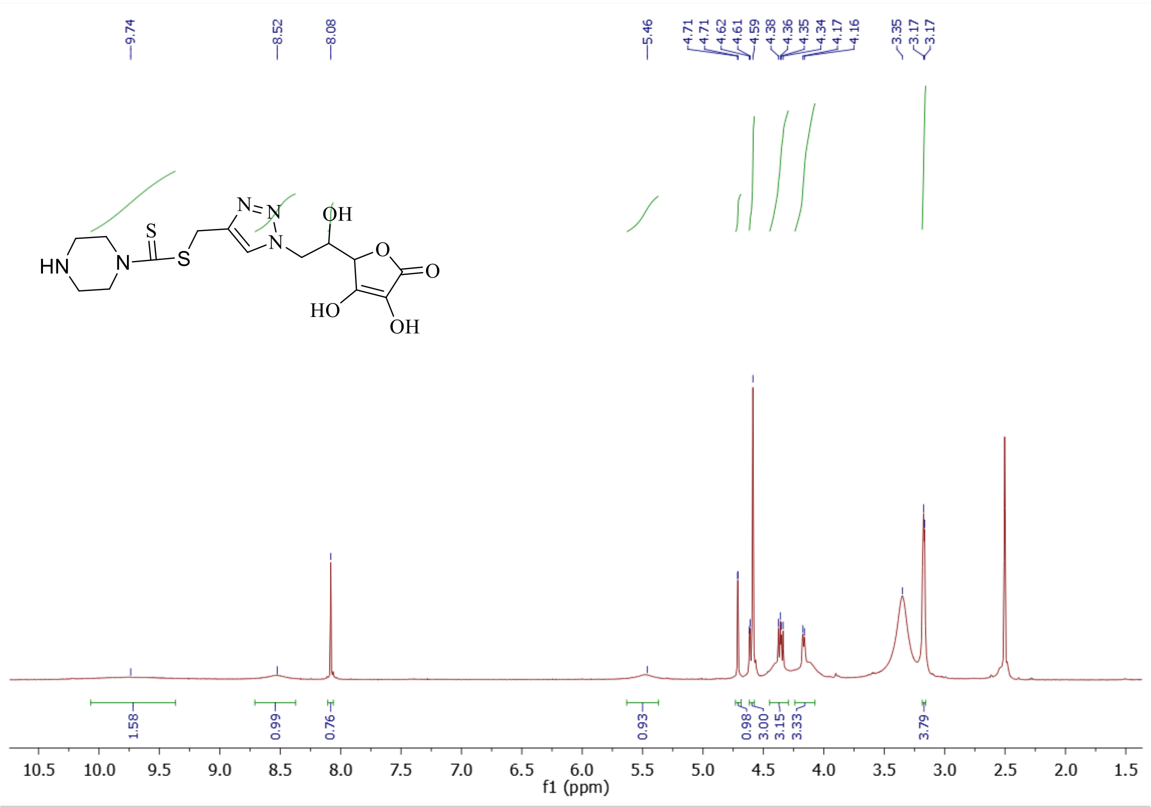

b)

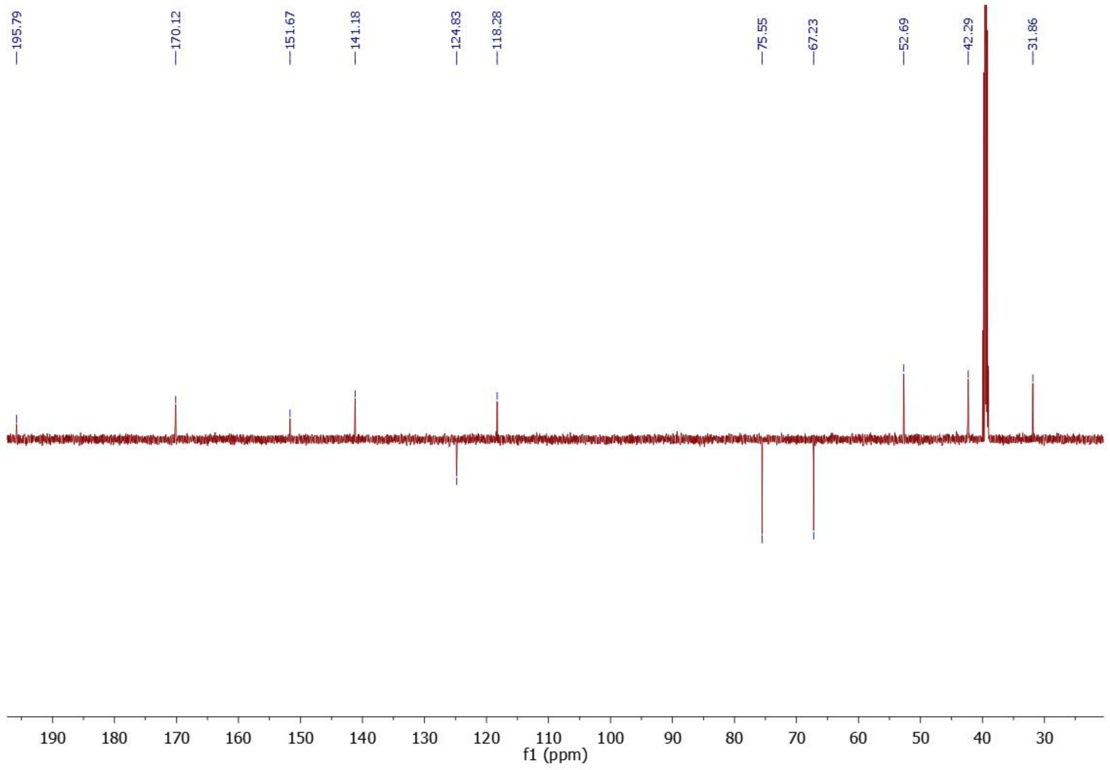

Figure S19. a) ^{1}H NMR and b) ^{13}C NMR of compd. **7c**.

a)

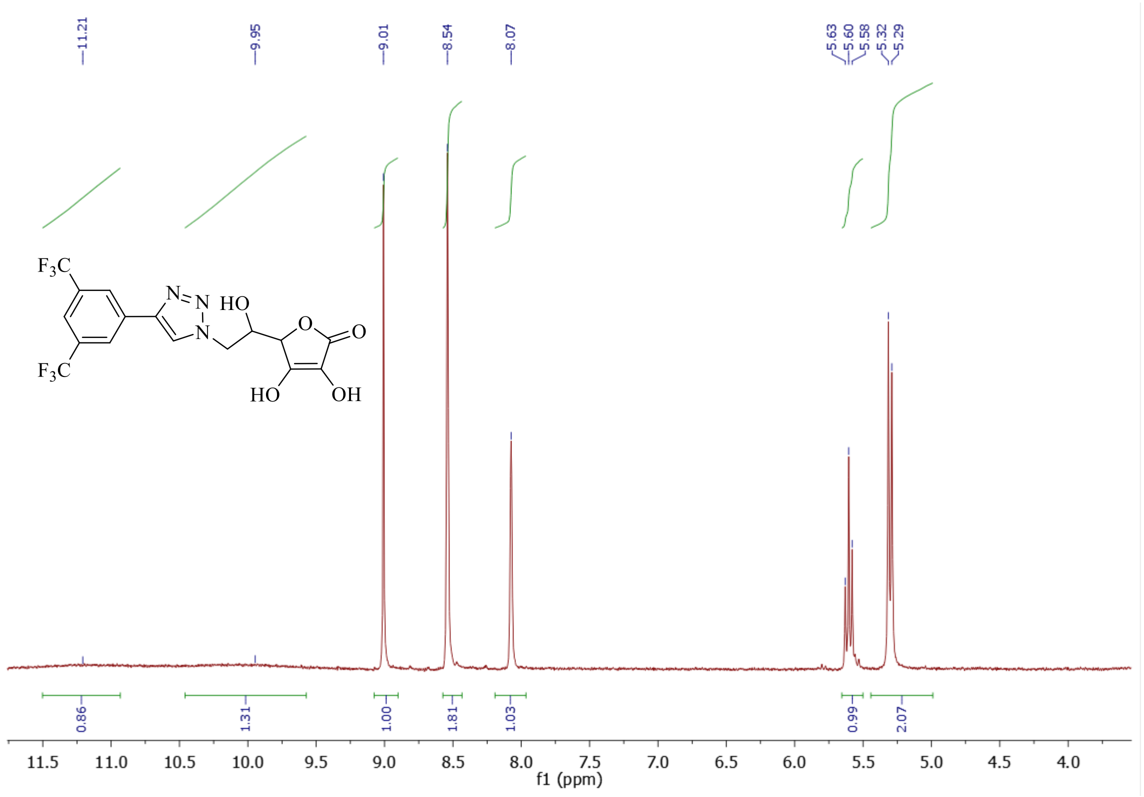

b)

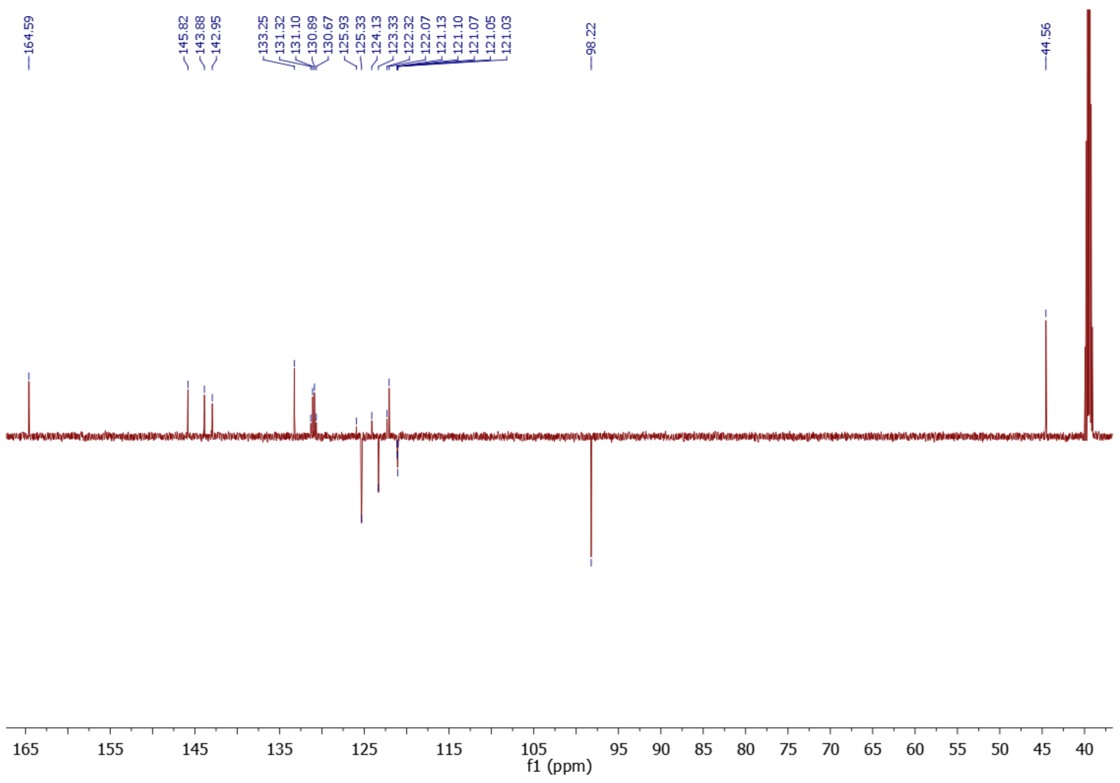

Figure S20. a) ^{1}H NMR and b) ^{13}C NMR of compd. **7d**.

a)

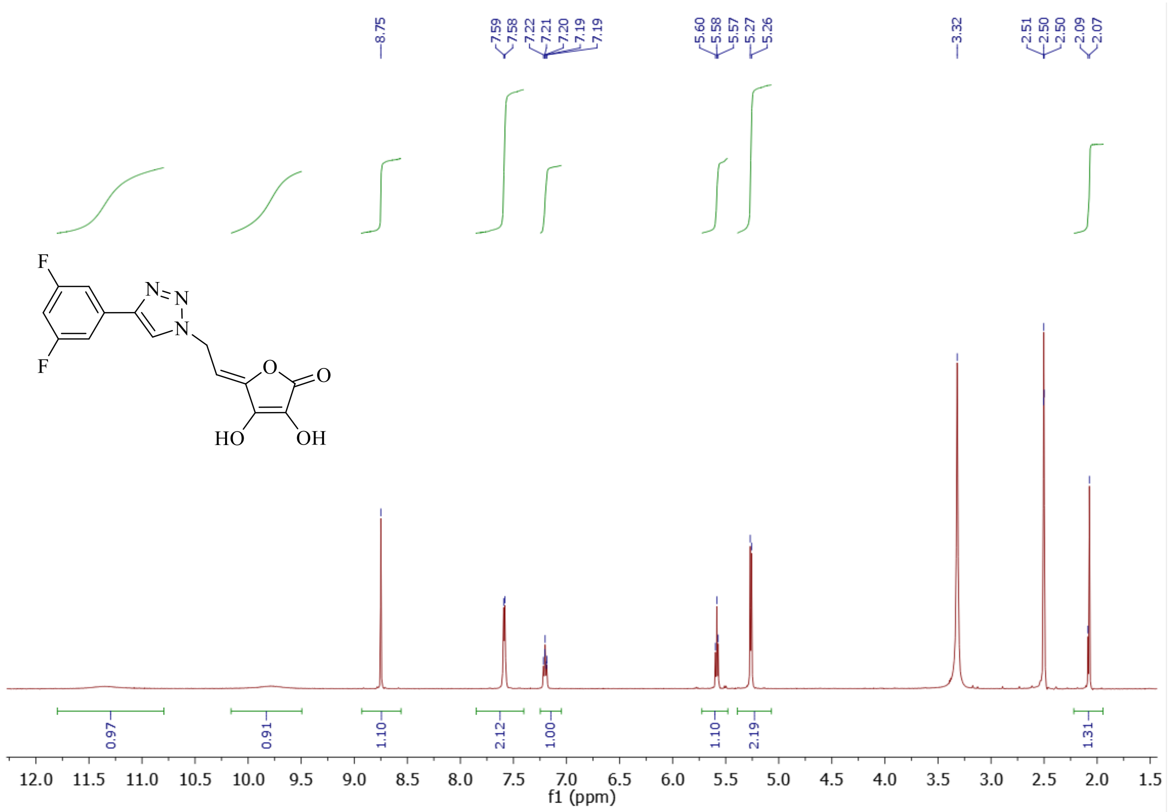

b)

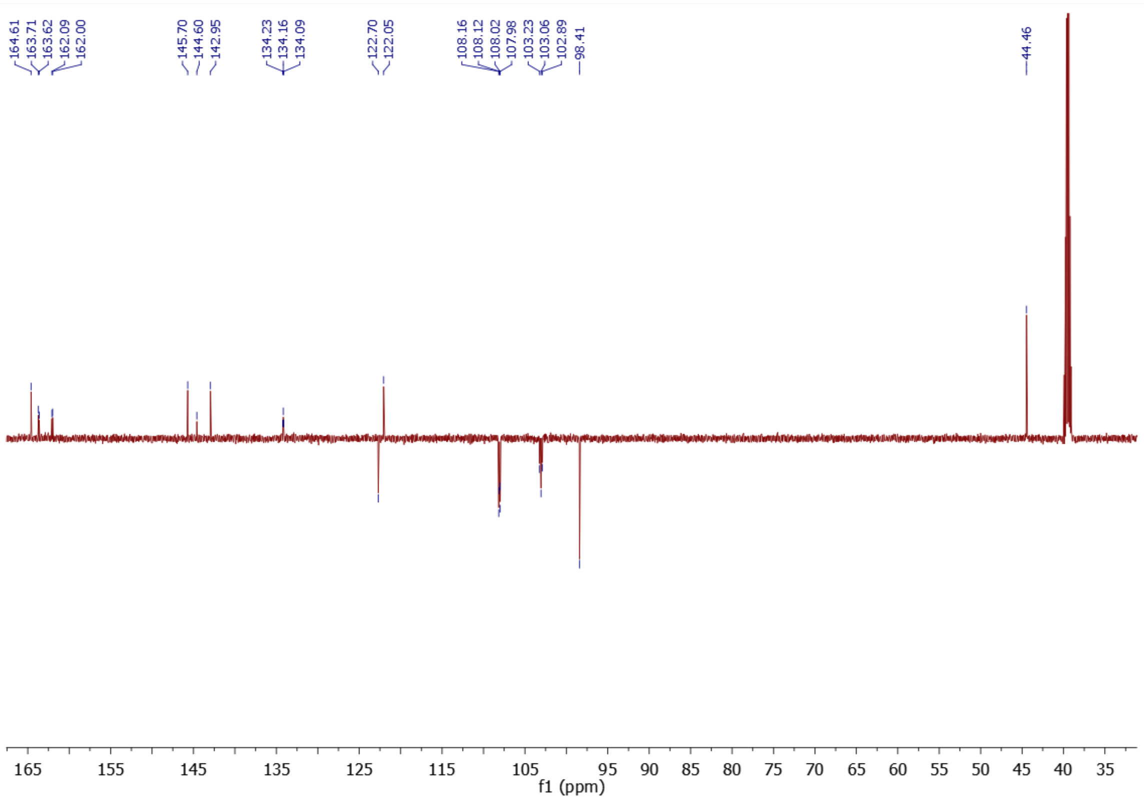

Figure S21. a) ^{1}H NMR and b) ^{13}C NMR of compd. **7e**.

a)

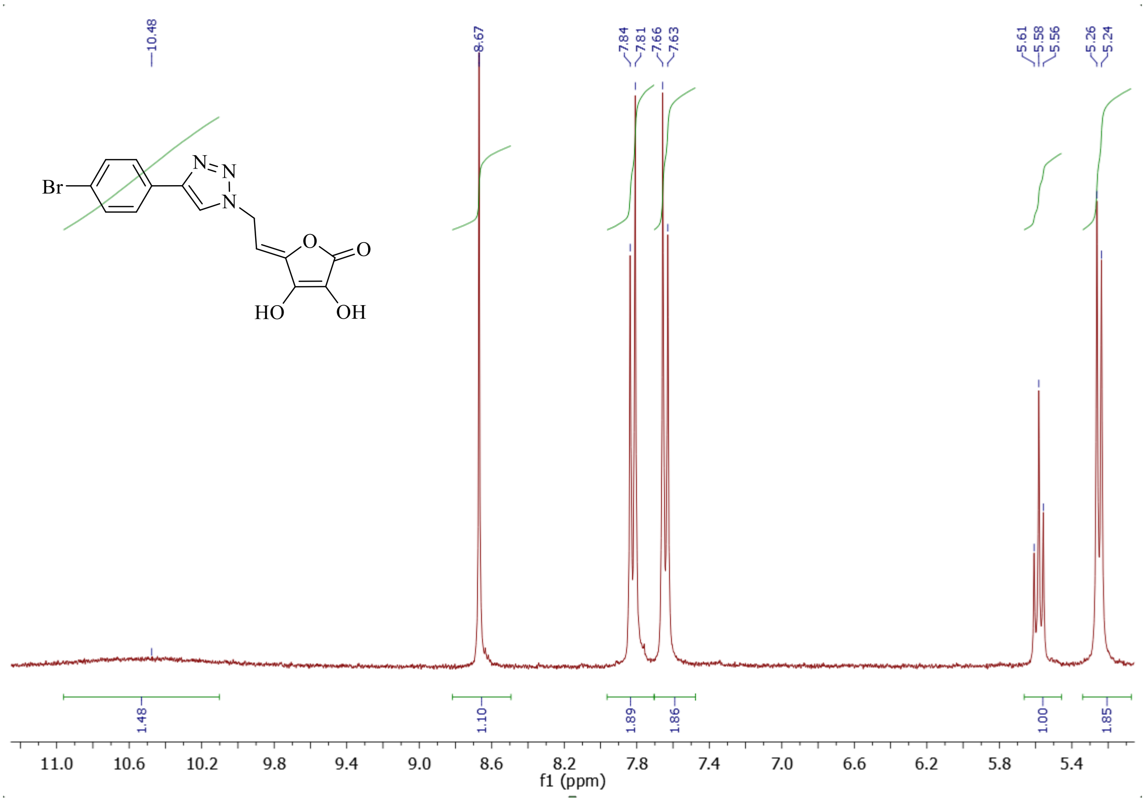

b)

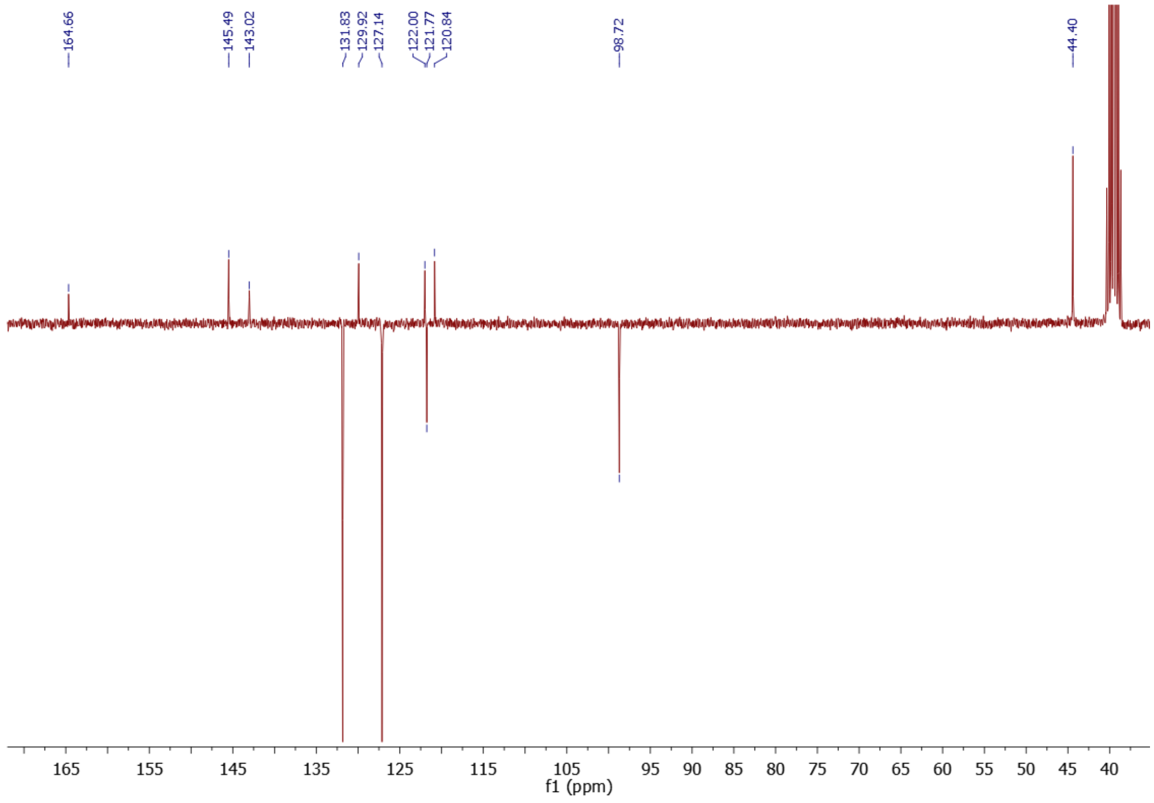

Figure S22. a) ^{1}H NMR and b) ^{13}C NMR of compd. **7f**.

a)

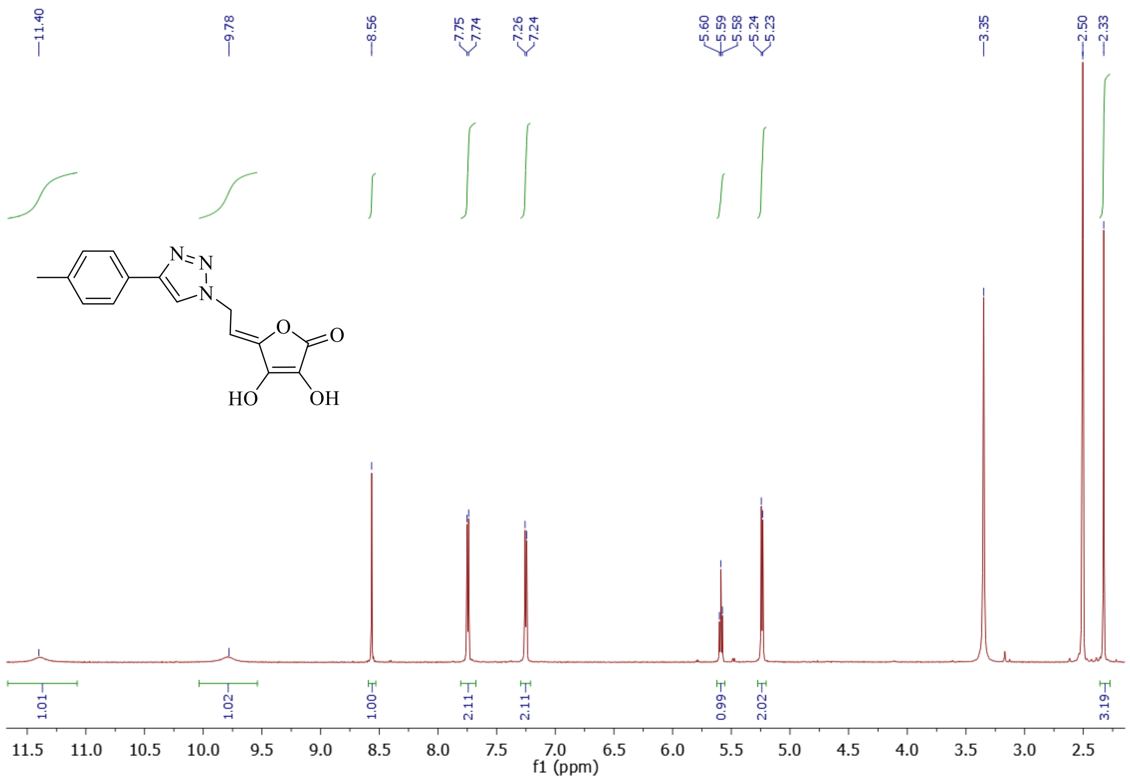

b)

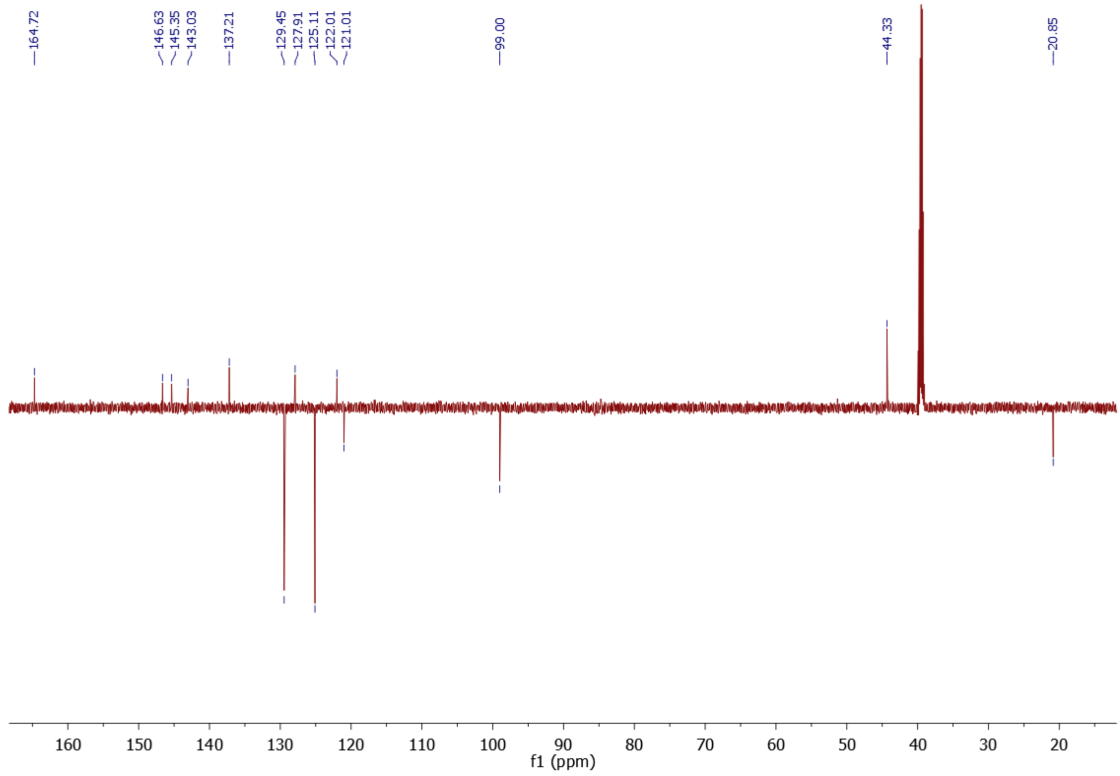

Figure S23. a) ^{1}H NMR and b) ^{13}C NMR of compd. **7g**.

a)

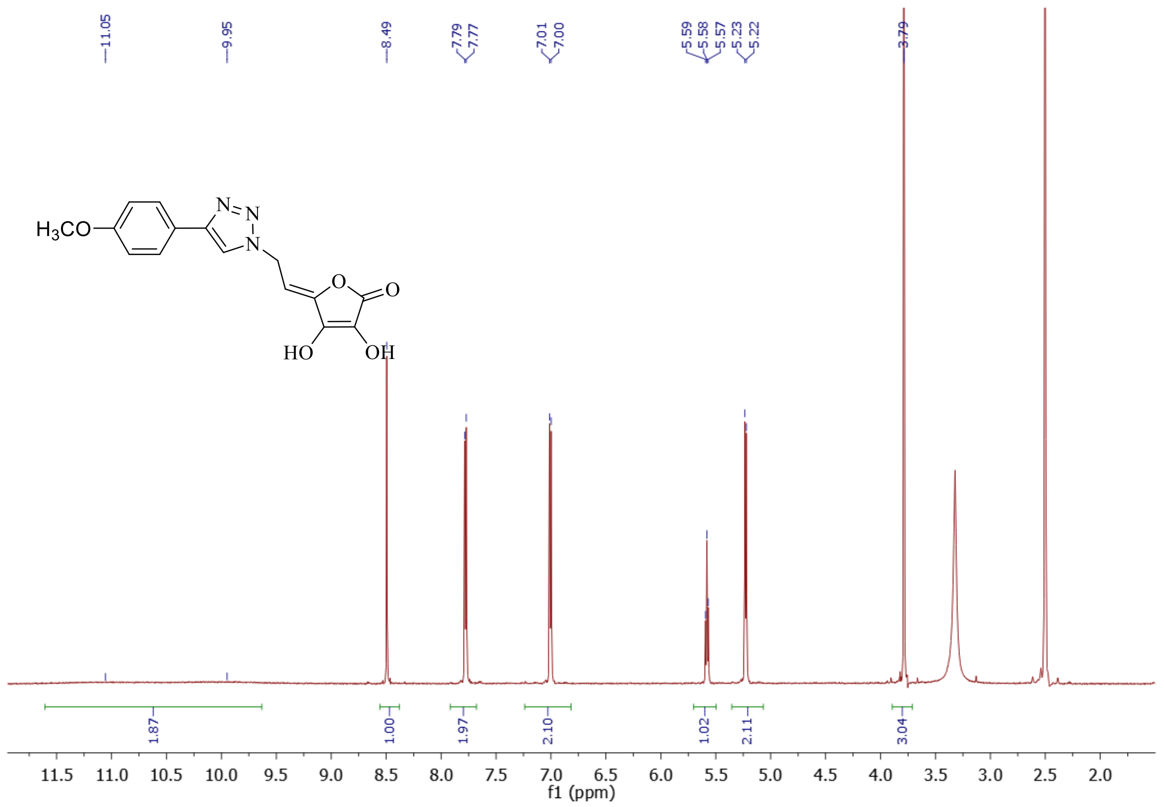

b)

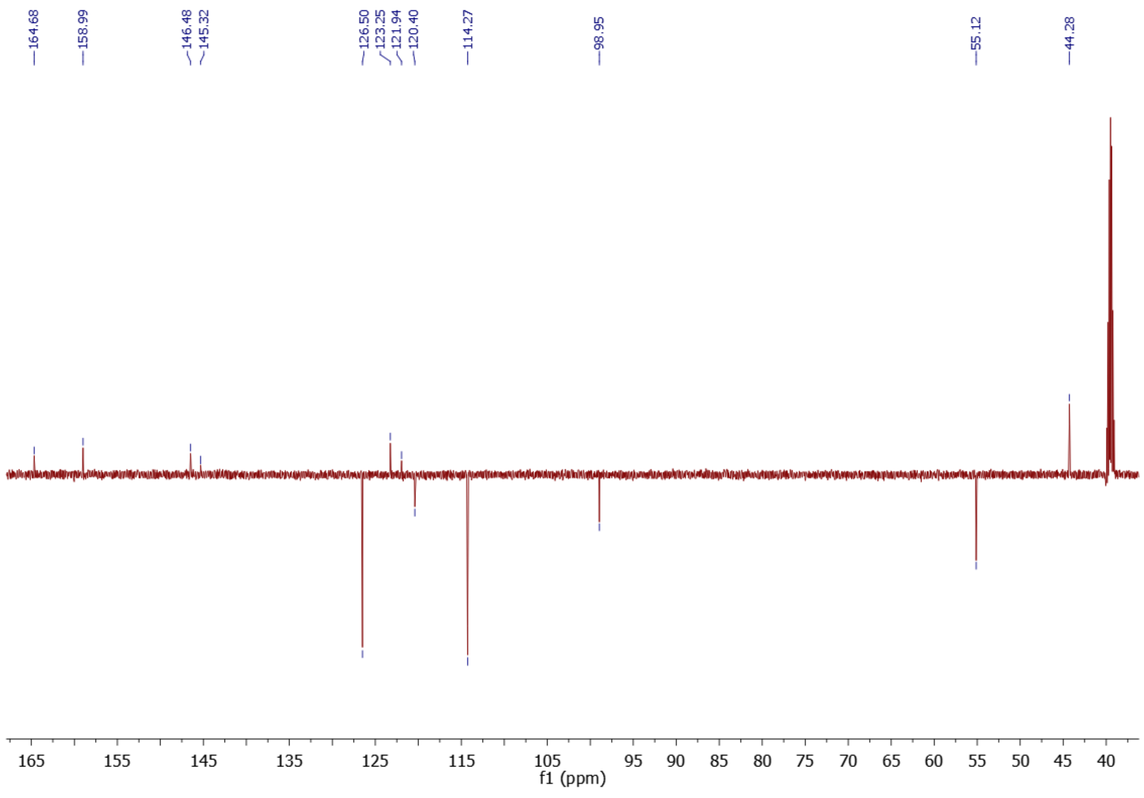

Figure S24. a) ^{1}H NMR and b) ^{13}C NMR of compd. **7i**.

a)

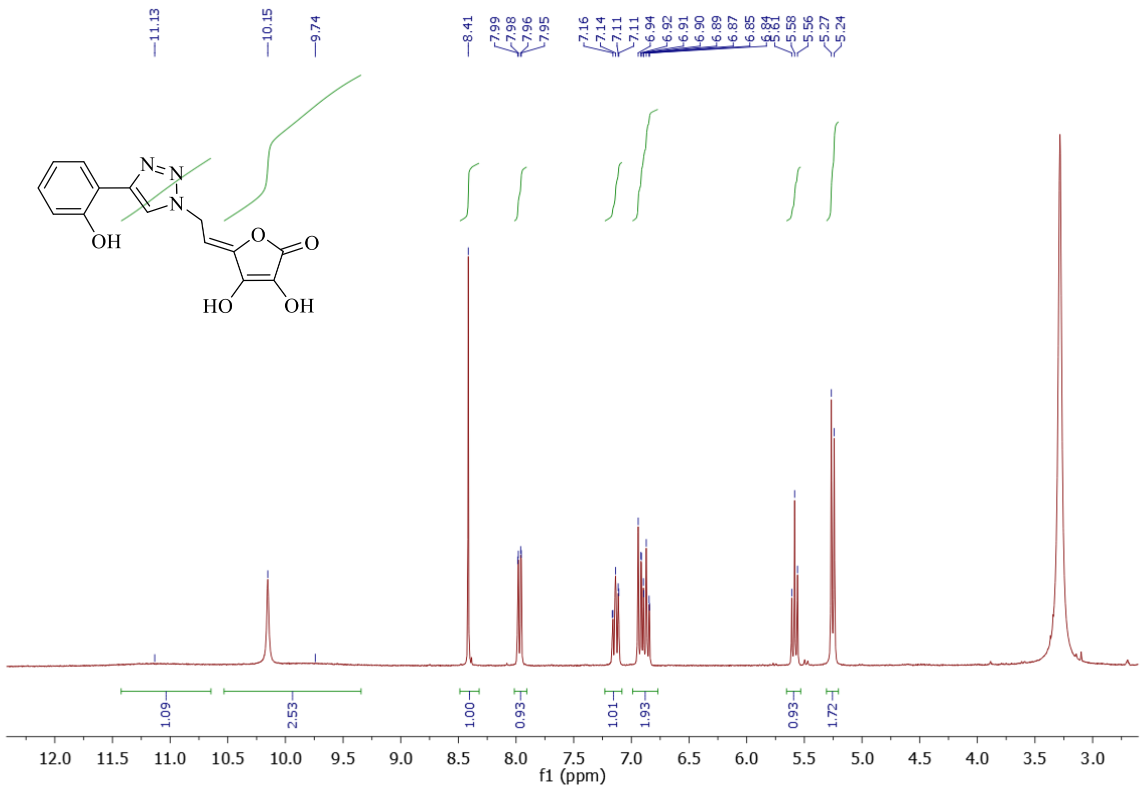

b)

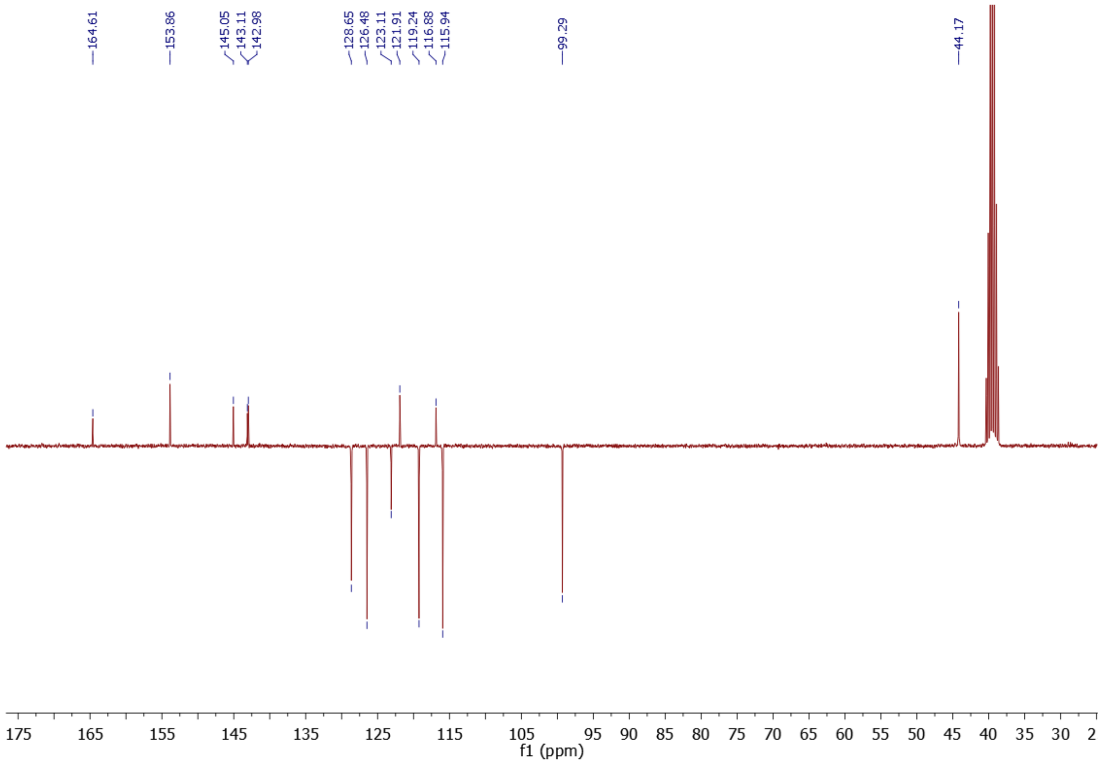

Figure S25. a) ^{1}H NMR and b) ^{13}C NMR of compd. **7j**.

a)

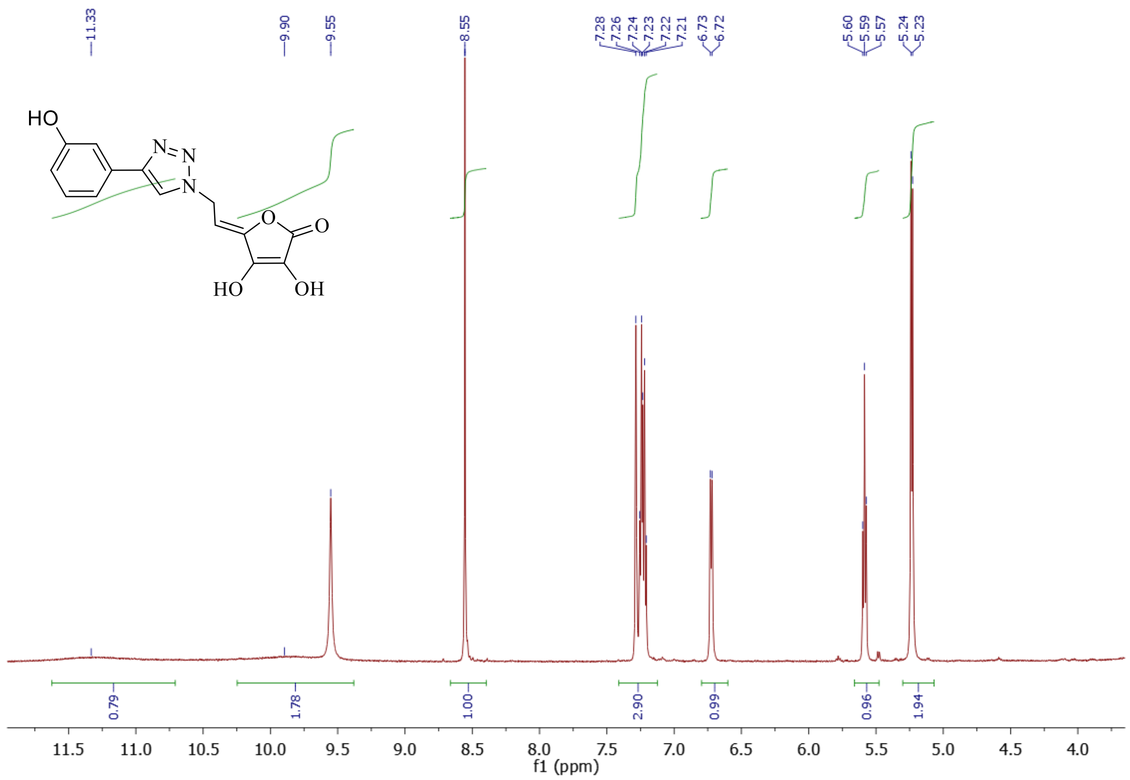

b)

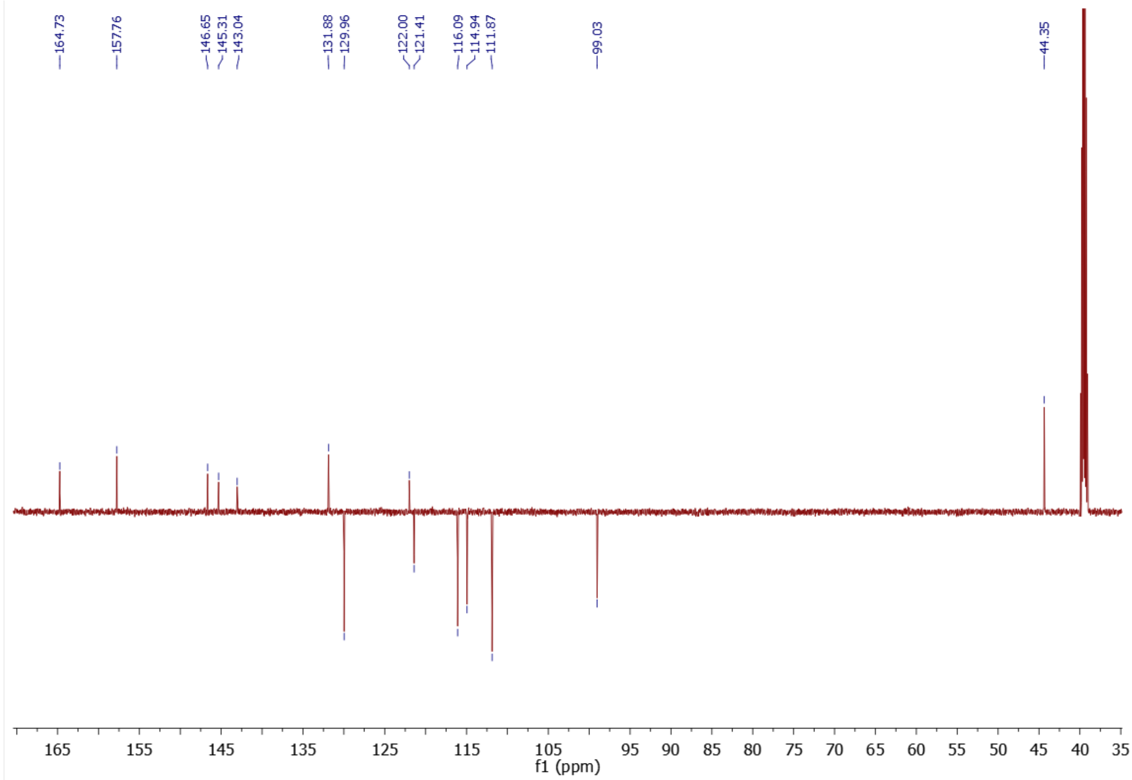

Figure S26. a) ^{1}H NMR and b) ^{13}C NMR of compd. **7k**.

a)

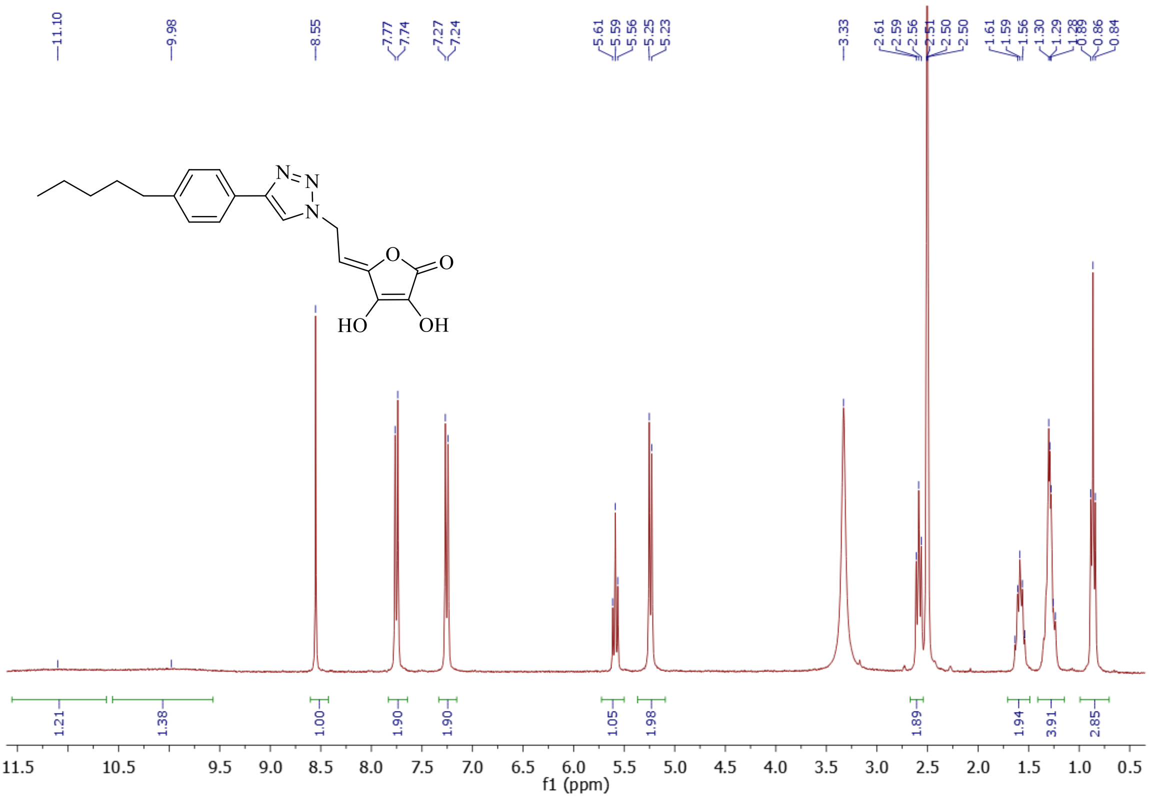

b)

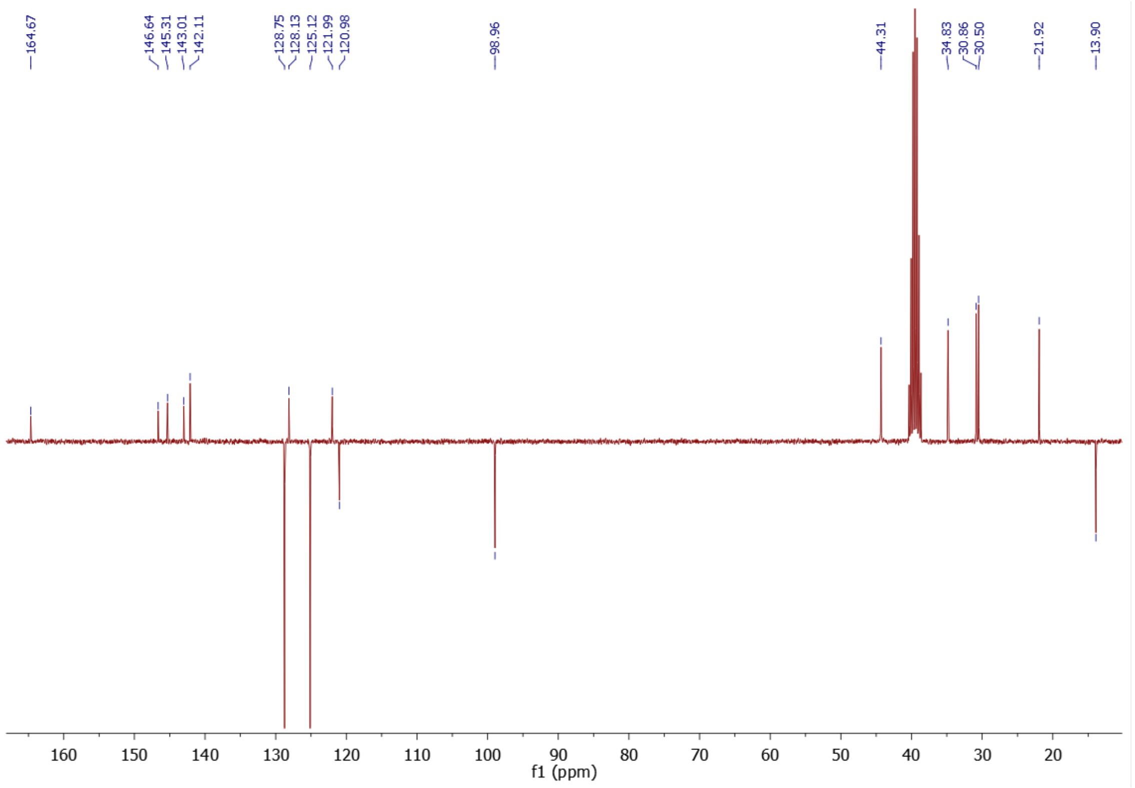

Figure S27. a) ^{1}H NMR and b) ^{13}C NMR of compd. **7l**.

a)

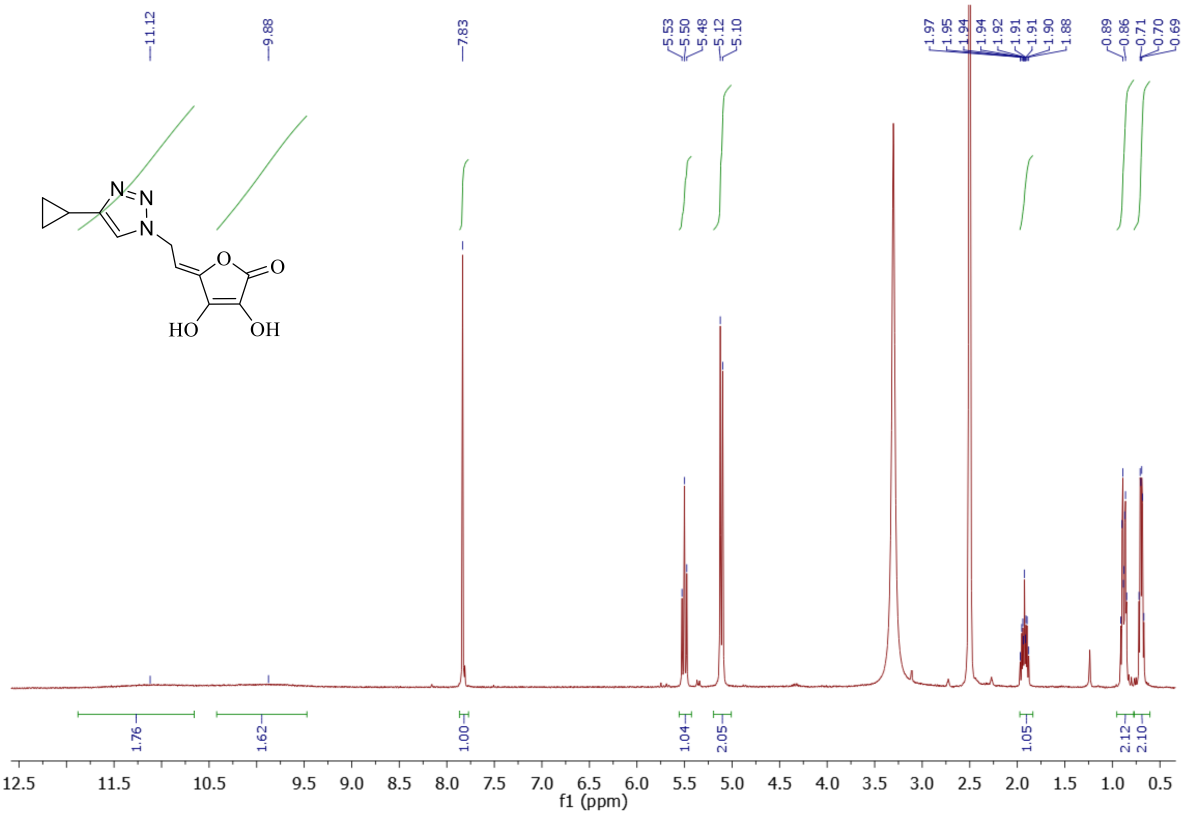

b)

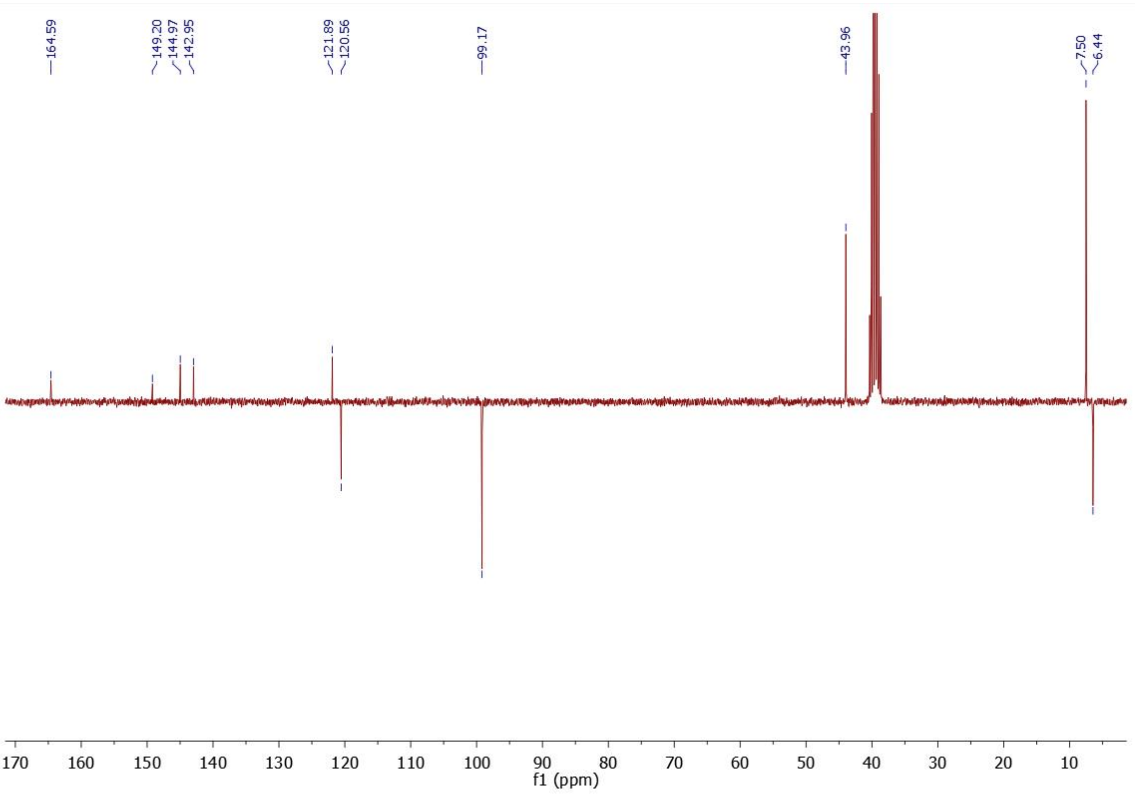

Figure S28. a) ^{1}H NMR and b) ^{13}C NMR of compd. **7m**.

a)

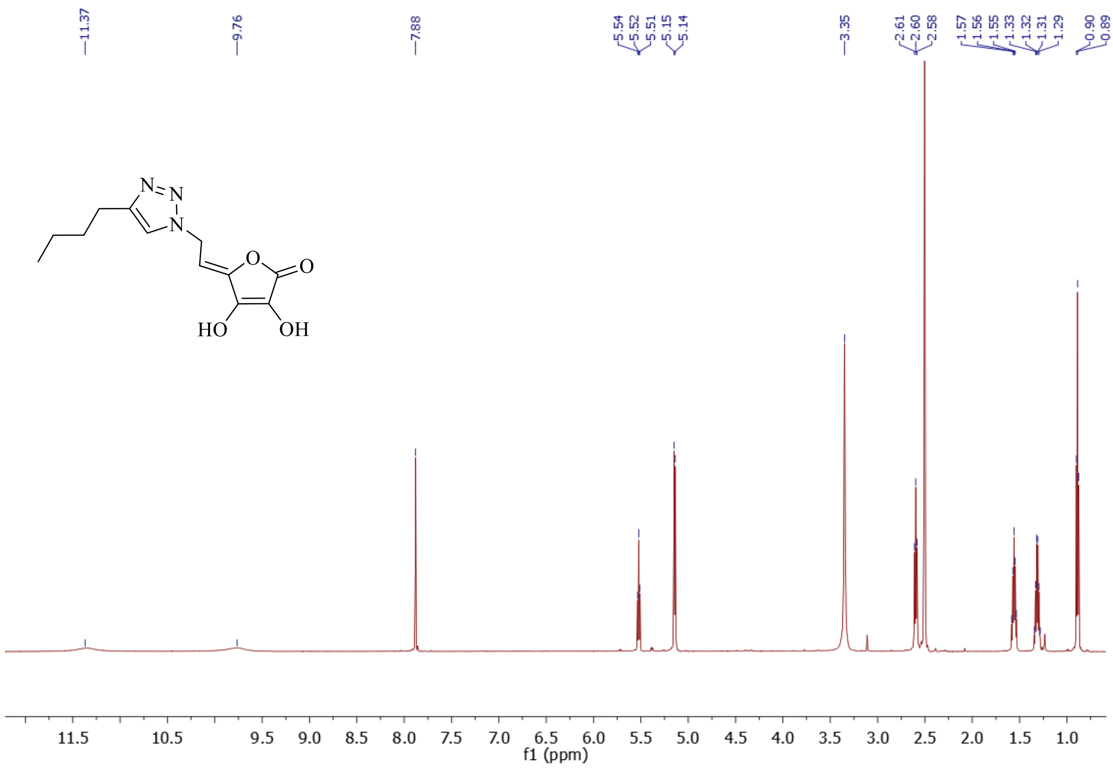

b)

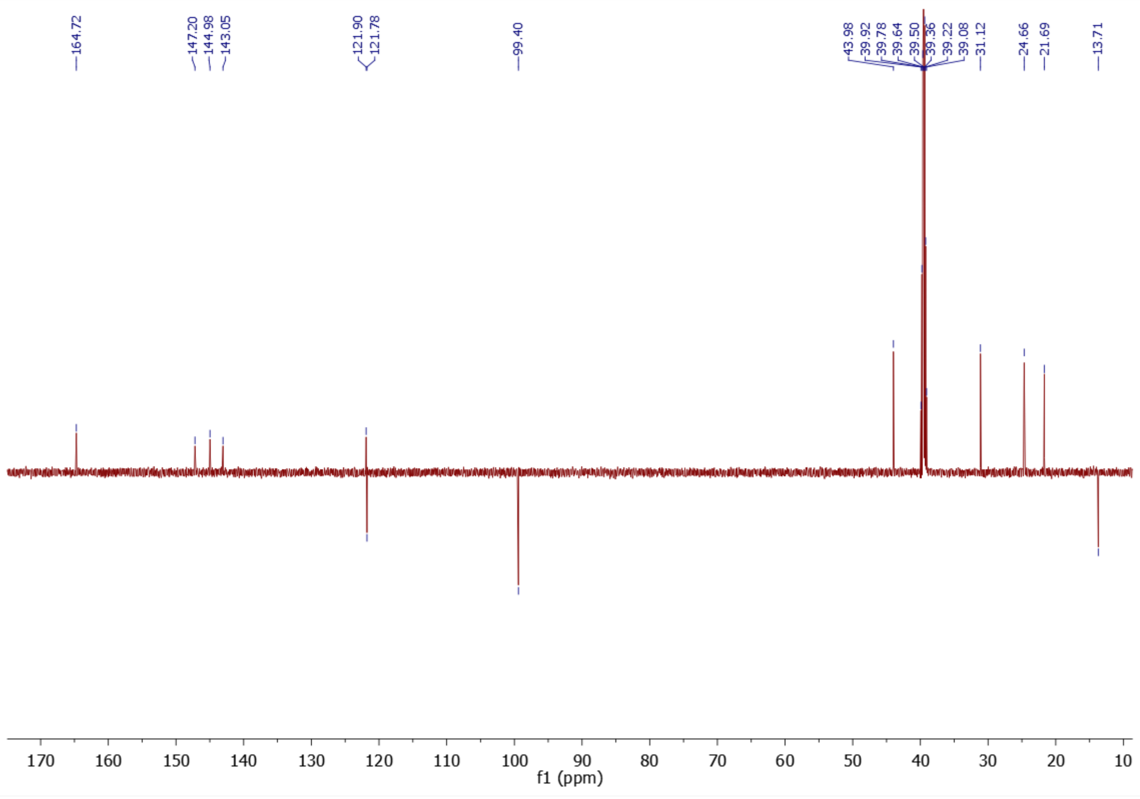

Figure S29. a) ^{1}H NMR and b) ^{13}C NMR of compd. **7n**.

a)

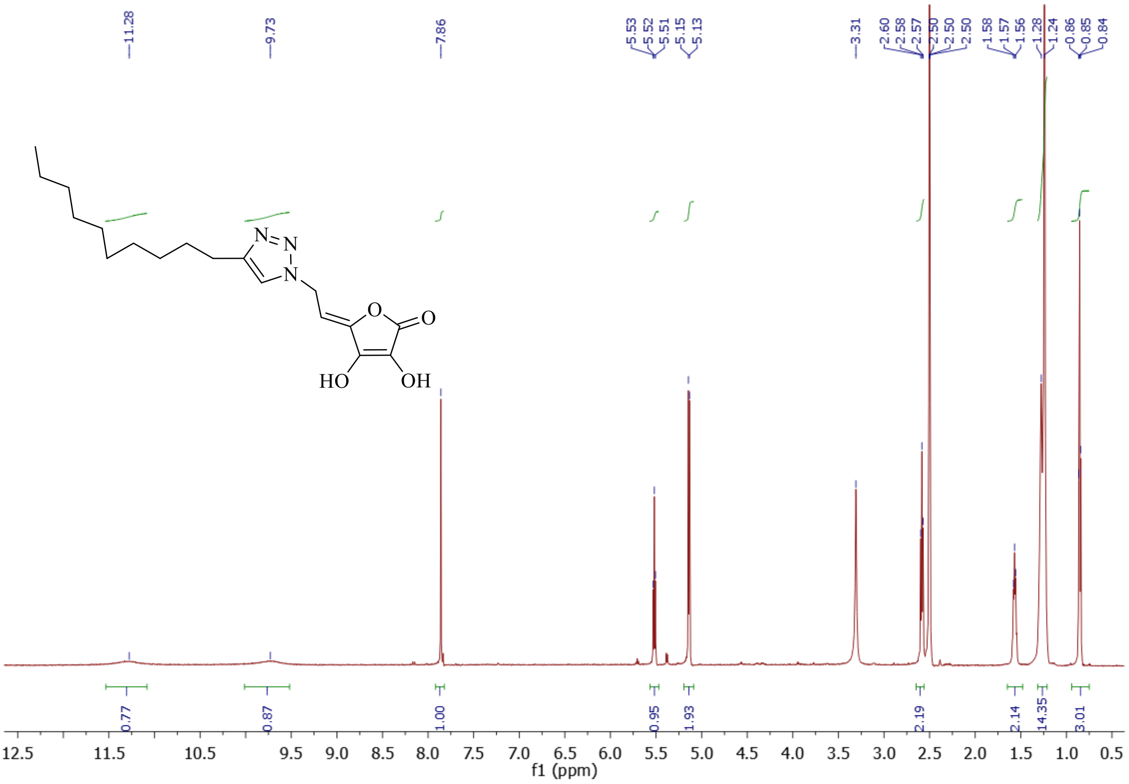

b)

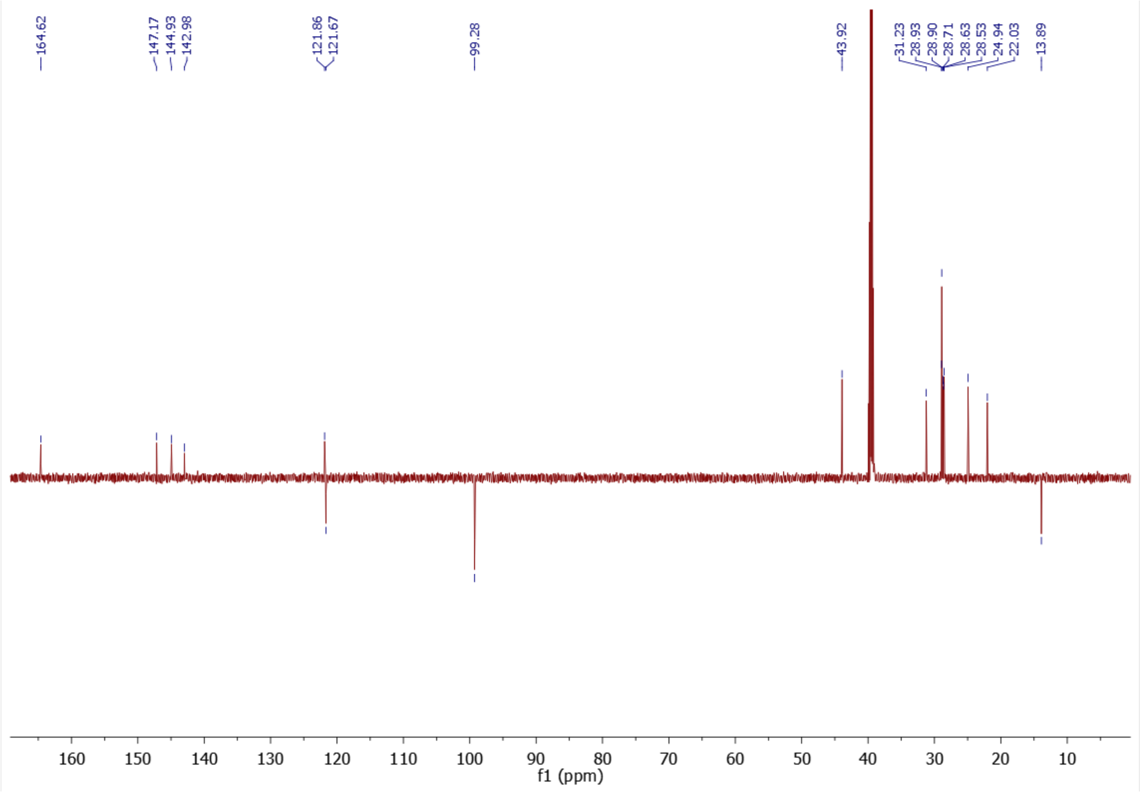

Figure S30. a) ^{1}H NMR and b) ^{13}C NMR of compd. **7o**.

a)

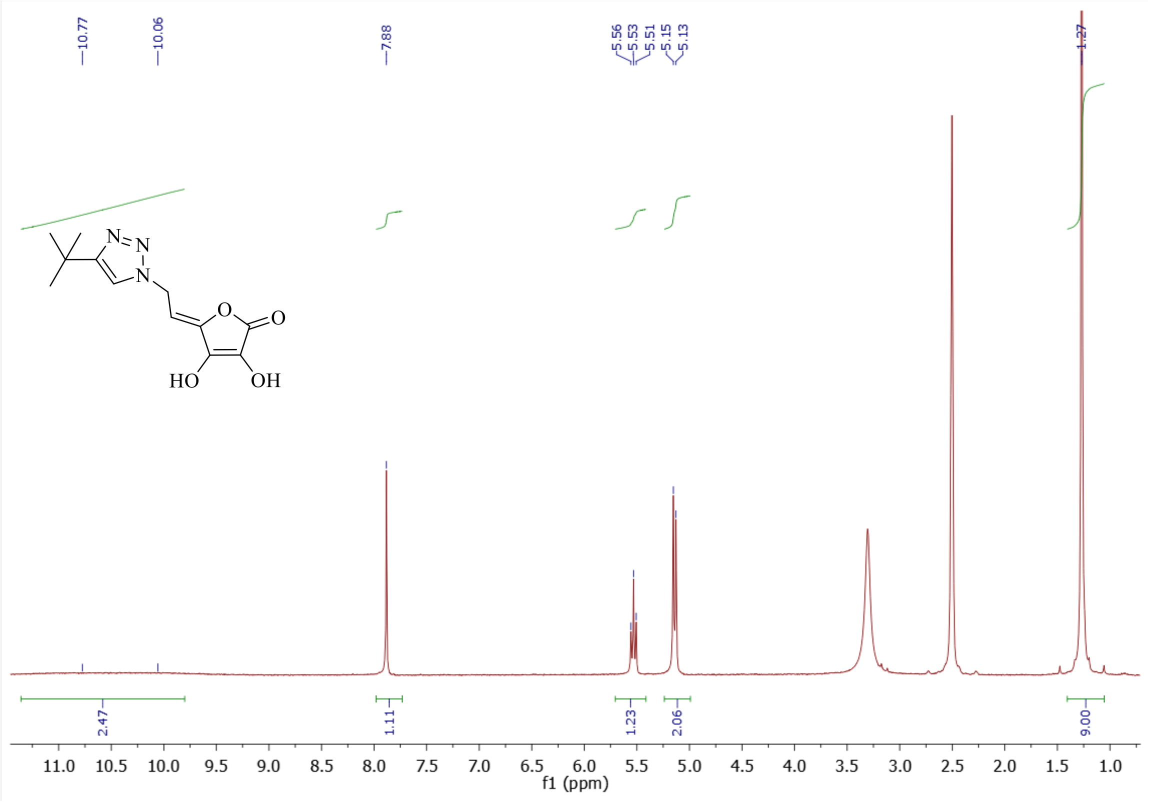

b)

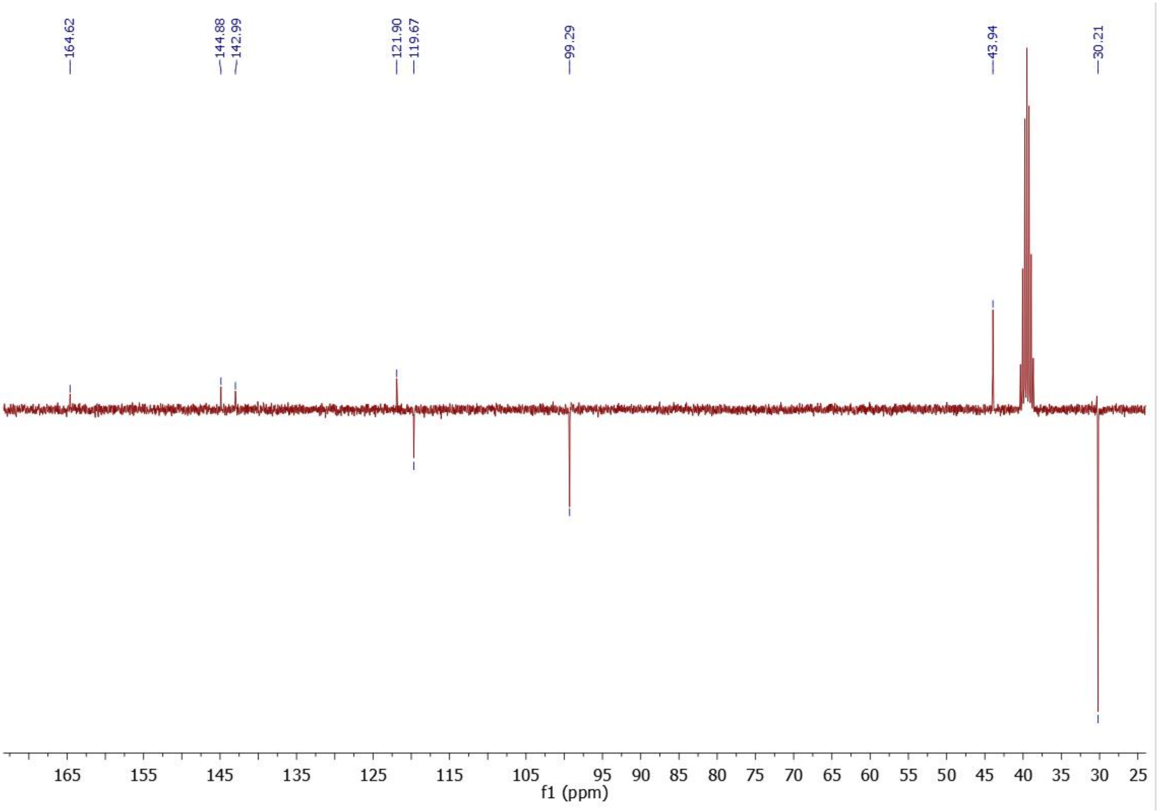

Figure S31. a) ^{1}H NMR and b) ^{13}C NMR of compd. **7p**.

a)

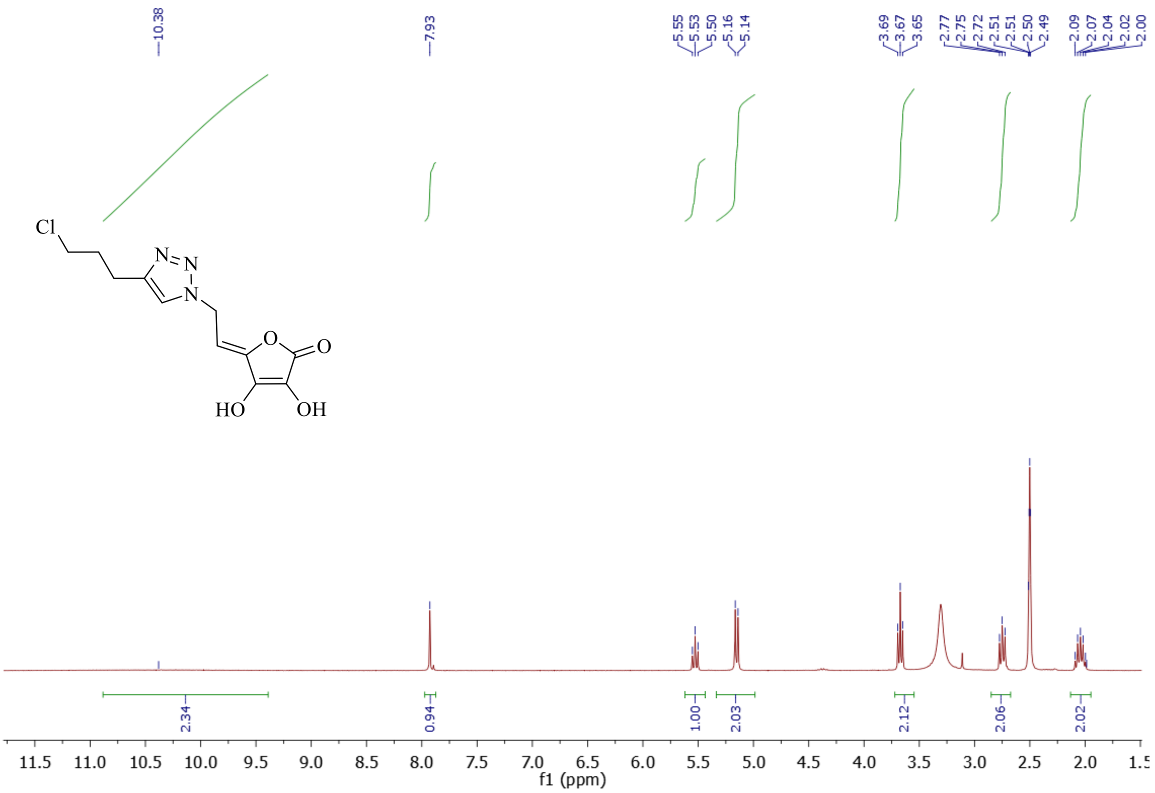

b)

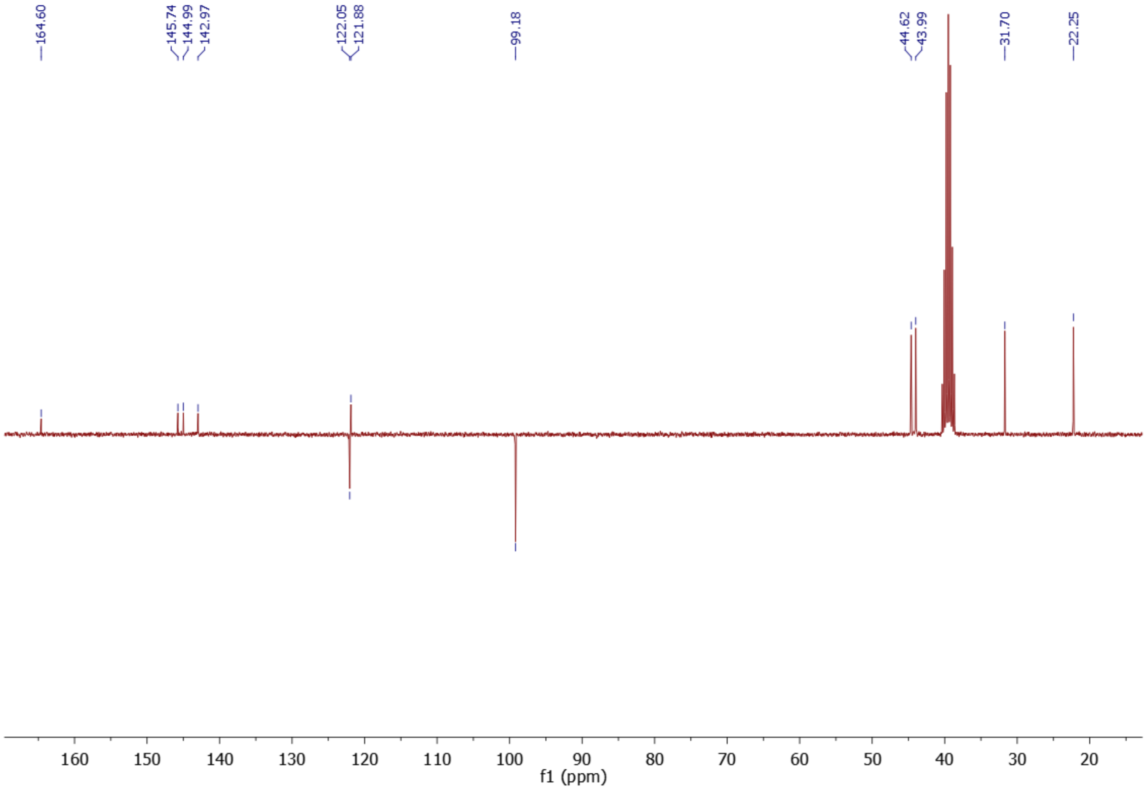

Figure S32 a) ^{1}H NMR and b) ^{13}C NMR of compd. **7q**.

a)

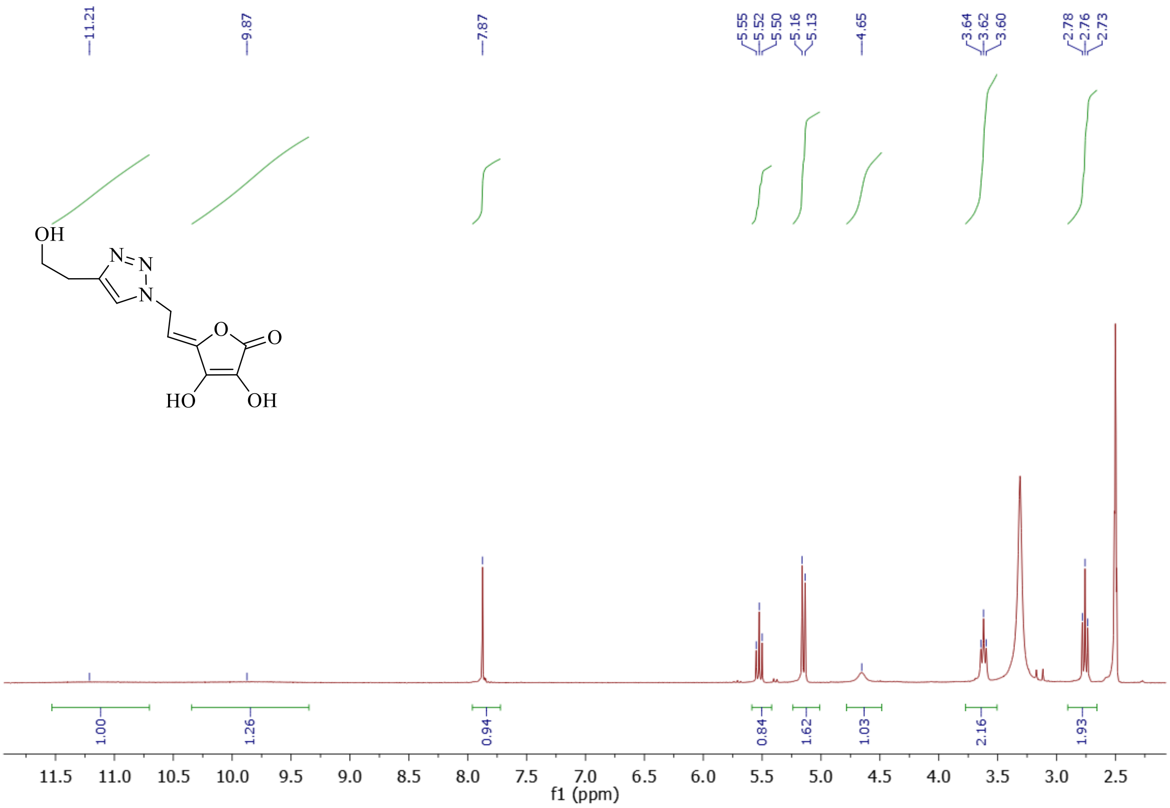

b)

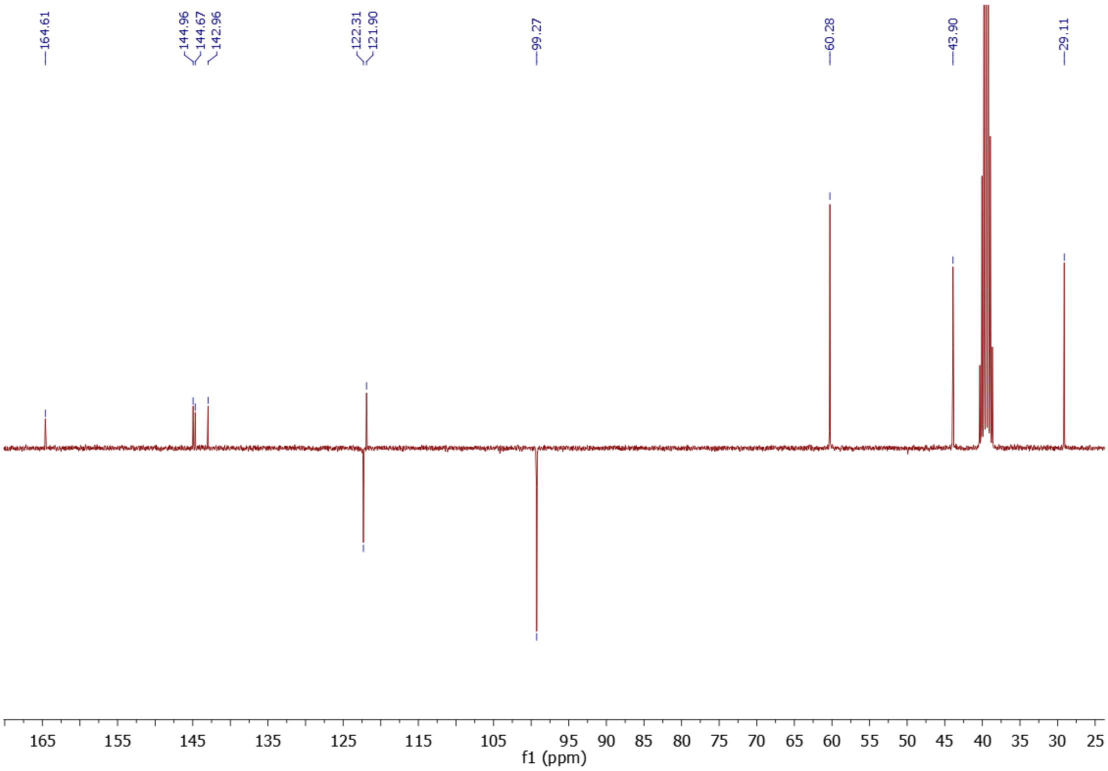

Supplement: Supplementary file 1 [file ijms-20-04735-s001.pdf]
